# Approximation of SDEs: a stochastic sewing approach

**DOI:** 10.1007/s00440-021-01080-2

**Published:** 2021-07-30

**Authors:** Oleg Butkovsky, Konstantinos Dareiotis, Máté Gerencsér

**Affiliations:** 1 Weierstrass Institute, Mohrenstraße 39, 10117 Berlin, Germany; 2 University of Leeds, Woodhouse, Leeds, LS2 9JT UK; 3 TU Vienna, Wiedner Hauptstrasse 8-10, 1040 Wien, Austria

**Keywords:** Stochastic differential equations, Regularization by noise, Irregular drift, Strong rate of convergence, Fractional Brownian motion, 60H50, 60H10, 65C30

## Abstract

We give a new take on the error analysis of approximations of stochastic differential equations (SDEs), utilizing and developing the stochastic sewing lemma of Lê (Electron J Probab 25:55, 2020. 10.1214/20-EJP442). This approach allows one to exploit regularization by noise effects in obtaining convergence rates. In our first application we show convergence (to our knowledge for the first time) of the Euler–Maruyama scheme for SDEs driven by fractional Brownian motions with non-regular drift. When the Hurst parameter is $H\in (0,1)$ and the drift is $\mathcal {C}^\alpha$, $\alpha \in [0,1]$ and $\alpha >1-1/(2H)$, we show the strong $L_p$ and almost sure rates of convergence to be $((1/2+\alpha H)\wedge 1) -\varepsilon$, for any $\varepsilon >0$. Our conditions on the regularity of the drift are optimal in the sense that they coincide with the conditions needed for the strong uniqueness of solutions from Catellier and Gubinelli (Stoch Process Appl 126(8):2323–2366, 2016. 10.1016/j.spa.2016.02.002). In a second application we consider the approximation of SDEs driven by multiplicative standard Brownian noise where we derive the almost optimal rate of convergence $1/2-\varepsilon$ of the Euler–Maruyama scheme for $\mathcal {C}^\alpha$ drift, for any $\varepsilon ,\alpha >0$.

## Introduction

Since the 1970s, it has been observed that the addition of a random forcing into an ill-posed deterministic system could make it well-posed. Such phenomenon is called *regularization by noise*. One of the prime examples concerns differential equations of the form

1.1 $$\begin{aligned} dX_t = b(X_t)\, dt, \end{aligned}$$

where *b* is a bounded vector field. While Eq. (1.1) might have infinitely many solutions when *b* fails to be Lipschitz continuous and might possess no solution when *b* fails to be continuous, Zvonkin [39] and Veretennikov [38] (see also the paper of Davie [9]) showed that the stochastic differential equation (SDE)

1.2 $$\begin{aligned} dX_t = b(X_t)\,dt + dB_t \end{aligned}$$

driven by a Brownian motion *B*, has a unique strong solution when *b* is merely bounded measurable. This result was extended to the case of the fractional Brownian noise in [4, 8, 27, 32, 33]. These papers study the equation

1.3 $$\begin{aligned} dX_t=b(X_t)\,dt+\,dB^H_t,\qquad X_0=x_0 \end{aligned}$$

where $B^H$ is a *d*-dimensional fractional Brownian motion with Hurst parameter $H\in (0,1)$. It is known [8, Theorem 1.9] that this equation has a unique strong solution if *b* belongs to the Hölder–Besov space $\mathcal {C}^\alpha$ and $\alpha >1-1/(2H)$. Thus, the presence of the noise not only produces solutions in situations where there was none but also singles out a unique physical solution in situations where there were multiple. However, to the best of our knowledge, no construction of this solution through discrete approximations has been known (unless $H=1/2$). In this article, we develop a new approach which allows to construct this solution and even obtain rate of convergence of the discrete approximations. Before the formal setup of Sect. 2, let us informally overview the results.

First, let us recall that in the standard Brownian case ($H=1/2$) the seminal work of Gyöngy and Krylov [18] established the convergence in probability of the Euler–Maruyama scheme

1.4 $$\begin{aligned} dX^n_t=b(X^n_{\kappa _n(t)})\,dt+\,dB^H_t,\qquad X_0^n=x_0^n,\quad t\geqslant 0 \end{aligned}$$

to the solution of (1.3). Here *b* is a bounded measurable function and

1.5 $$\begin{aligned} \kappa _n(t):=\lfloor nt\rfloor /n, \quad n\in \mathbb {N}. \end{aligned}$$

In the present paper, we significantly extend these results by (a) establishing the convergence of the Euler–Maruyama scheme for all $H\in (0,1)$; (b) showing that the convergence takes place in a stronger ($L_p(\Omega )$ and almost sure) sense; (c) obtaining the explicit rate of convergence. More precisely, in Theorem 2.1 we show that if *b* is bounded and Hölder-continuous with exponent $\alpha >1-1/(2H)$, then the Euler–Maruyama scheme converges with rate $((1/2+\alpha H)\wedge 1)-\varepsilon$ for any $\varepsilon >0$. Thus, the approximation results are obtained under the minimal assumption on the drift *b* that is needed for strong uniqueness of solutions [8, 32] and for the well-posedness of scheme (1.4). Let us also point out that in particular, for $H<1/2$, one does not need to require any continuity from *b* to obtain a convergence rate $1/2-\varepsilon$. Concerning approximations of SDEs driven by fractional Brownian motions with regular coefficients, we refer the reader to the recent works [15, 22] and references therein. Concerning the implementation of such schemes and in particular the simulation of increments of fractional Brownian motions we refer to [37, Section 6] and its references.

Our second application is to study equations with multiplicative noise in the standard Brownian case:

1.6 $$\begin{aligned} dX_t=b(X_t)\,dt+\sigma (X_t)\,dB_t,\qquad X_0=x_0,\quad t\geqslant 0 \end{aligned}$$

and their discretisations

1.7 $$\begin{aligned} dX^{n}_t=b(X^{n}_{\kappa _n(t)})\,dt+\sigma (X^{n}_{\kappa _n(t)})\,dB_t,\quad X_0^{n}=x_0^n, \quad t\geqslant 0. \end{aligned}$$

Here *b*, $\sigma$ are measurable functions, *B* is a *d*-dimensional Brownian motion, and $\kappa _n$ is defined in (1.5). To ensure well-posedness, a nondegeneracy assumption on $\sigma$ has to be assumed. In the standard Brownian case the rate of convergence for irregular *b* has been recently actively studied, see among many others [2, 28–30, 36] and their references. However, the obtained rate deteriorates as *b* becomes more irregular: in the setting of (1.6)-(1.7), the best known rate is only proven to be (at least) $\alpha /2$ for $b\in \mathcal {C}^\alpha$, $\alpha >0$ in [2].

It was first shown in [10] that, at least for additive noise, the strong rate does *not* vanish as the regularity $\alpha$ approaches 0, and one in fact recovers the rate $1/2-\varepsilon$ for arbitrary $\varepsilon >0$, for all $\alpha >0$. In the present paper we establish the same for multiplicative noise, in which case the rate 1/2 is well-known to be optimal. Our proof offers several other improvements to earlier results: all moments of the error can be treated in the same way, the scalar and multidimensional cases are also not distinguished, and the main error bound (2.9) is uniform in time, showing that $X_\cdot$ and $X^{n}_\cdot$ are close as paths. The topology (in time) where the error is measured is in fact even stronger, see Remark 2.3.

To obtain these results we develop a new strategy which utilizes the stochastic sewing lemma (SSL) of Lê [27] as well as some other specially developed tools. We believe that these tools might be also of independent interest; let us briefly describe them here.

First, we obtain a new stochastic sewing–type lemma, see Theorem 3.3. It provides bounds on the $L_p$-norm of the increments of a process, with the correct dependence on *p*. This improves the corresponding bounds from SSL of Lê (although, under more restrictive conditions). This improved bound is used for proving stretched exponential moment bounds that play a key role in the convergence analysis of the Euler–Maruyama scheme for (1.3), see Sect. 4.3. In particular, using this new sewing-type lemma, we are able to extend the key bound of Davie [9, Proposition 2.1] (this bound was pivotal in his paper for establishing uniqueness of solutions to (1.2) when the driving noise is the standard Brownian motion) to the case of the fractional Brownian noise, see Lemma 4.3.

Second, in Sect. 5 we derive density estimates of (a drift-free version of) the solution of (1.7) via Malliavin calculus. Classical results in this direction include that of Gyöngy and Krylov [18], and of Bally and Talay [5, 6]: the former gives sharp short time asymptotics but no smoothness of the density, and the latter vice versa (see Remark 5.1 below). Since our approach requires both properties at the same time, we give a self-contained proof of such an estimate (5.2).

Finally let us mention that, as in [10, 11, 34], efficient quadrature bounds play a crucial role in the analysis. These are interesting approximation problems in their own right, see, e.g., [25] and the references therein. Such questions in the non-Markovian setting of fractional Brownian motion have only been addressed recently in [1]. However, there are a few key differences to our quadrature bounds from Lemma 4.1. First, we derive bounds in $L_p(\Omega )$ for all *p*, which by Proposition 2.9 also imply the corresponding almost sure rate (as opposed to $L_2(\Omega )$ rates only in [1]). Second, unlike the standard fractional Brownian motions considered here, [1] requires starting them at time 0 from a random variable with a density, which provides a strong smoothing effect. Third, when approximating the functional of the form

$$\begin{aligned} \Gamma _t:=\int _0^tf(B^H_s)\,ds, \end{aligned}$$

also called ‘occupation time functional’, by the natural discretisation

$$\begin{aligned} \Gamma ^n_t=\int _0^tf(B^H_{\kappa _n(s)})\,ds, \end{aligned}$$

our results not only imply pointwise error estimates on $|\Gamma _T-\Gamma ^n_T|$, but also on the error of the whole path $\Vert \Gamma _{\cdot }-\Gamma ^n_\cdot \Vert _{\mathcal {C}^\beta }$ measured in a Hölder norm $\mathcal {C}^\beta$ with some $\beta >1/2$. This is an immediate consequence of the bounds (4.1) in combination with Kolmogorov’s continuity theorem.

The rest of the article is structured as follows. Our main results are presented in Sect. 2. In Sect. 3 we outline the main strategy and collect some necessary auxiliary results, including the new sewing lemma–type bound Theorem 3.3. Section 4 is devoted to the error analysis in the additive fractional noise case. In Sect. 5 we prove an auxiliary bound on the probability distribution of the Euler–Maruyama approximation of certain sufficiently nice SDEs. The proofs of the convergence in the multiplicative standard Brownian noise case are given in Sect. 6.

## Main results

We begin by introducing the basic notation. Consider a probability space $(\Omega , \mathcal {F}, \mathbb {P})$ carrying a *d*-dimensional two-sided Brownian motion $(W_t)_{t \in \mathbb {R}}$. Let $\mathbb {F}=(\mathcal {F}_t)_{t \in \mathbb {R}}$ be the filtration generated by the increments of *W*. The conditional expectation given $\mathcal {F}_s$ is denoted by $\;\;{\mathbb {E}}\;^s$. For $H \in (0,1)$ we define the fractional Brownian motion with Hurst parameter *H* by the Mandelbrot-van Ness representation [35, Proposition 5.1.2]

2.1 $$\begin{aligned} B^H_t := \int _{-\infty }^0 \bigl (|t-s|^{H-1/2}- |s|^{H-1/2}\bigr ) \, dW_s + \int _0^t |t-s|^{H-1/2} \, dW_s. \end{aligned}$$

Recall that the components of $B^H$ are independent and each component is a Gaussian process with zero mean and covariance

2.2 $$\begin{aligned} C(s,t):=\frac{c_H}{2}(s^{2H}+t^{2H}-|t-s|^{2H}),\quad s,t\geqslant 0, \end{aligned}$$

where $c_H$ is a certain positive constant, see [35, (5.1)].

For $\alpha \in (0,1]$ and a function $f:Q\rightarrow V$, where $Q\subset \mathbb {R}^k$ and $(V,|\cdot |)$ is a normed space, we set

$$\begin{aligned}{}[f]_{\mathcal {C}^\alpha (Q,V)}:=\sup _{x\ne y\in Q}\frac{|f(x)-f(y)|}{|x-y|^\alpha }. \end{aligned}$$

For $\alpha \in (0,\infty )$ we denote by $\mathcal {C}^\alpha (Q,V)$ the space of all functions $f:Q\rightarrow V$ having derivatives $\partial ^\ell f$ for all multi-indices $\ell \in ( \mathbb {Z}_+)^k$ with $|\ell |<\alpha$ such that

$$\begin{aligned} \Vert f\Vert _{\mathcal {C}^\alpha (Q,V)}:=\sum _{|\ell |< \alpha } \sup _{x\in Q}|\partial ^\ell f(x)|+ \sum _{\alpha -1< |\ell |< \alpha }[\partial ^\ell f]_{\mathcal {C}^{\alpha -|\ell |}(Q,V)}< \infty . \end{aligned}$$

If $\ell =(0,\ldots ,0)$, then as usual, we use the convention $\partial ^\ell f=f$. In particular, the $\mathcal {C}^\alpha$ norm always includes the supremum of the function. We also set $\mathcal {C}^0(Q,V)$ to be the space of bounded measurable functions with the supremum norm. We emphasize that in our notation elements of $\mathcal {C}^0$ need *not* be continuous! If $\alpha <0$, then by $\mathcal {C}^\alpha (\mathbb {R}^d,\mathbb {R})$ we denote the space of all distributions $f \in \mathcal {D}'( \mathbb {R}^d)$, such that

$$\begin{aligned} \Vert f \Vert _{\mathcal {C}^\alpha } := \sup _{\varepsilon \in (0,1]} \varepsilon ^{-\alpha /2} \Vert \mathcal {P}_\varepsilon f\Vert _{\mathcal {C}^0(\mathbb {R}^d,\mathbb {R})}< \infty , \end{aligned}$$

where $\mathcal {P}_\varepsilon f$ is the convolution of *f* with the *d*-dimensional Gaussian heat kernel at time $\varepsilon$.

In some cases we use shorthands: if $Q=\mathbb {R}^d$, or $V=\mathbb {R}^d$ or $V=\mathbb {R}^{d\times d}$, they are omitted from the notation. For instance, the reader understands that requiring the diffusion coefficient $\sigma$ of (1.6) to be of class $\mathcal {C}^\alpha$ is to require it to have finite $\Vert \cdot \Vert _{\mathcal {C}^\alpha (\mathbb {R}^d,\mathbb {R}^{d\times d})}$ norm. If $V=L_p(\Omega )$ for some $p\geqslant 2$, we write

2.3 $$\begin{aligned}{}[] f []_{\mathscr {C}^\alpha _p,Q}:=\Vert f\Vert _{\mathcal {C}^\alpha (Q,L_p(\Omega ))}. \end{aligned}$$

*Convention on constants*: throughout the paper *N* denotes a positive constant whose value may change from line to line; its dependence is always specified in the corresponding statement.

### Additive fractional noise

Our first main result establishes the convergence of the numerical scheme (1.4) to the solution of Eq. (1.3). Fix $H\in (0,1)$. It is known ( [8, Theorem 1.9]) that if the drift $b\in \mathcal {C}^\alpha$ with $\alpha \in [0,1]$ satisfying $\alpha >1-1/(2H)$, then for any fixed $x_0\in \mathbb {R}^d$, Eq. (1.3) admits a unique strong solution, which we denote by *X*. For any $n\in \mathbb {N}$ we take $x_0^n\in \mathbb {R}^d$ and denote the solution of (1.4) by $X^n$. For a given $\alpha \in [0,1]$ and $H\in (0,1)$, we set

2.4 $$\begin{aligned} \gamma =\gamma (\alpha ,H):=(1/2+\alpha H)\wedge 1. \end{aligned}$$

Now we are ready to present our first main result. Its proof is placed in Sect. 4, a brief outline of it is provided in Sect. 3.1.

#### Theorem 2.1

Let $$\alpha \in [0,1]$$ satisfy

2.5 $$\begin{aligned} \alpha >1-1/(2H). \end{aligned}$$

Suppose $b\in \mathcal {C}^\alpha$, let $\varepsilon ,\delta >0$ and $p\geqslant 2$. Then there exists a constant $\tau =\tau (\alpha ,H,\varepsilon )>1/2$ such that for all $n\in \mathbb {N}$ the following bound holds

2.6 $$\begin{aligned} \Vert X-X^n\Vert _{\mathcal {C}^\tau ([0,1],L_p(\Omega ))}\leqslant N n^{\delta }|x_0-x^n_0| + N n^{-\gamma +\varepsilon +\delta } \end{aligned}$$

with some constant $N=N(p,d,\alpha ,H,\varepsilon ,\delta ,\Vert b\Vert _{\mathcal {C}^\alpha })$.

#### Remark 2.2

An interesting question left open is whether one can reach $\alpha =0$ in the $H=1/2$ case. In dimension 1, this is positively answered [10] using PDE methods, but the sewing approach at the moment does not seem to handle such endpoint situations. For $H\ne 1/2$ even weak existence or uniqueness is not known for the endpoint $\alpha =1-1/(2H)$.

#### Remark 2.3

From (2.6), Kolmogorov’s continuity theorem, and Jensen’s inequality, one gets the bound

2.7 $$\begin{aligned} \big \Vert \Vert X-X^n\Vert _{\mathcal {C}^{\tau -\varepsilon '}([0,1],\mathbb {R}^d)}\big \Vert _{L_p(\Omega )}\leqslant N n^\delta |x_0-x^n_0| + N n^{-\gamma +\varepsilon +\delta }. \end{aligned}$$

for any $\varepsilon '>0$ (with *N* also depending on $\varepsilon '$). In the literature it is more common to derive error estimates in supremum norm, which of course follows:

$$\begin{aligned} \big \Vert \sup _{t\in [0,1]}|X_t-X^n_t|\big \Vert _{L_p(\Omega )}\leqslant N n^\delta |x_0-x^n_0| + N n^{-\gamma +\varepsilon +\delta }, \end{aligned}$$

but (2.7) is quite a bit stronger.

#### Remark 2.4

A trivial lower bound on the rate of convergence of the solutions is the rate of convergence of the initial conditions. In (1.4) we lose $\delta$ compared to this rate, but $\delta >0$ can be chosen arbitrarily small. This becomes even less of an issue if one simply chooses $x_0^n=x_0$.

#### Remark 2.5

The fact that the error is well-controlled even between the gridpoints is related to the choice of how we extend $X^n$ to continuous time from the points $X_0^n,X_{1/n}^n,\ldots$. For other type of extensions and their limitations we refer the reader to [31].

#### Corollary 2.6

Assume $\alpha \in [0,1]$ satisfies (2.5) and suppose $b\in \mathcal {C}^\alpha$. Take $x_0=x_0^n$ for all $n\in \mathbb {N}$. Then for a sufficiently small $\theta >0$ and any $\varepsilon >0$ there exists an almost surely finite random variable $\eta$ such that for all $n\in \mathbb {N}$, $\omega \in \Omega$ the following bound holds

$$\begin{aligned} \sup _{t\in [0,1]}|X_t-X^n_t|\leqslant \Vert X-X^n\Vert _{\mathcal {C}^{1/2+\theta }([0,1],\mathbb {R}^d)} \leqslant \eta n^{-\gamma +\varepsilon }, \end{aligned}$$

where $\gamma$ was defined in (2.4).

#### Proof

An immediate consequence of (2.7), Proposition 2.9 below, and the fact that $\tau >1/2$. $\square$

### Multiplicative Brownian noise

In the multiplicative case we work under the ellipticity and regularity conditions

2.8 $$\begin{aligned} \sigma \in \mathcal {C}^2,\qquad \qquad \sigma \sigma ^T\succeq \lambda I, \end{aligned}$$

in the sense of positive definite matrices, with some $\lambda >0$. This, together with $b\in \mathcal {C}^0$, guarantees the strong well-posedness of equations (1.6) and (1.7) [38, Theorem 1], whose solutions we denote by *X* and $X^{n}$, respectively. The second main result then reads as follows, its proof is the content of Sect. 6.

#### Theorem 2.7

Let $\alpha \in (0,1]$. Suppose $b\in \mathcal {C}^\alpha$, let $\varepsilon >0$, $\tau \in [0,1/2)$, and $p\geqslant 2$. Suppose $\sigma$ satisfies (2.8). Then for all $n\in \mathbb {N}$ the following bound holds

2.9 $$\begin{aligned} \Vert X-X^n\Vert _{\mathcal {C}^\tau ([0,1],L_p(\Omega ))}\leqslant N|x_0-x_0^n| + N n^{-1/2+\varepsilon } \end{aligned}$$

with some $$N=N(p,d,\alpha ,\varepsilon ,\tau ,\lambda ,\Vert b\Vert _{\mathcal {C}^\alpha }, \Vert \sigma \Vert _{\mathcal {C}^2})$$.

#### Corollary 2.8

Let $\alpha \in (0,1]$, assume $x_0=x_0^n$ for all $n\in \mathbb {N}$, suppose $b\in \mathcal {C}^\alpha$, and suppose $\sigma$ satisfies (2.8). Let $\varepsilon >0$, $\tau \in [0,1/2)$. Then there exists an almost surely finite random variable $\eta$ such that for all $n\in \mathbb {N}$, $\omega \in \Omega$ the following bound holds

$$\begin{aligned} \sup _{t\in [0,1]}|X_t-X^n_t|\leqslant \Vert X-X^n\Vert _{\mathcal {C}^{\tau }([0,1],\mathbb {R}^d)} \leqslant \eta n^{-1/2+\varepsilon }. \end{aligned}$$

#### Proof

An immediate consequence of (2.9), Kolmogorov’s continuity theorem, and Proposition 2.9 below. $\square$

Let us conclude by invoking a simple fact used in the proof of Corollaries 2.6 and 2.8, which goes back to at least [20, proof of Theorem 2.3] (see also [13, Lemma 2]).

#### Proposition 2.9

Let $\rho >0$ and let $(Z_n)_{n\in \mathbb {N}}$ be a sequence of random variables such that for all $p>0$ and all $n\in \mathbb {N}$ one has the bound

$$\begin{aligned} \Vert Z_n\Vert _{L_p(\Omega )}\leqslant N n^{-\rho } \end{aligned}$$

for some $N=N(p)$. Then for all $\varepsilon >0$ there exists an almost surely random variable $\eta$ such that for all $n\in \mathbb {N}$, $\omega \in \Omega$

$$\begin{aligned} |Z_n|\leqslant \eta n^{-\rho +\varepsilon }. \end{aligned}$$

#### Proof

Notice that for any $q>0$

$$\begin{aligned} \sum _{n\in \mathbb {N}}\mathbb {P}(|Z_n|>n^{-\rho +\varepsilon })\leqslant \sum _{n\in \mathbb {N}}\frac{\;\;{\mathbb {E}}\;|Z_n|^q}{n^{q(-\rho +\varepsilon )}}\leqslant \sum _{n\in \mathbb {N}} N n^{-q\varepsilon }. \end{aligned}$$

Choosing $q=2/\varepsilon$, the above sum is finite, so by the Borel-Cantelli lemma there exists an almost surely finite $\mathbb {N}$-valued random variable $n_0$ such that $|Z_n|\leqslant n^{-\rho +\varepsilon }$ for all $n>n_0$. This yields the claim by setting

$$\begin{aligned} \eta :=1\vee \max _{n\leqslant n_0}(|Z_n|n^{\rho -\varepsilon }). \end{aligned}$$

$$\square $$

## Preliminaries

### The outline of the strategy

The purpose of this section is to outline the main steps in a simple example. Hopefully this gives a clear picture of the strategy to the reader, which otherwise may be blurred by the some complications arising in the proofs of Theorems 2.1 and 2.7.

The ‘simple example’ will be the setting of (1.3) and (1.4) with $H=1/2$ and $f\in \mathcal {C}^\alpha$ for some $\alpha >0$. We furthermore assume $x_0=x_0^n$ and that the time horizon is given by $[0,T_0]$ instead of [0, 1], with some small $1\geqslant T_0>0$ to be chosen later. Finally, we will only aim to prove (2.6) with $\tau =1/2$.

*Step 1 (“Quadrature bounds”)*. Our first goal is to bound the quantity

3.1 $$\begin{aligned} \mathcal {A}_{T_0}:=\int _0^{T_0} b(B_r)-b(B_{\kappa _n(r)})\,dr. \end{aligned}$$

From the Hölder continuity of *b*, one would have the trivial bound of order $n^{-\alpha /2}$ in any $L_p(\Omega )$ norm, but in fact one can do much better, as follows. Fix $\varepsilon \in (0,1/2)$ and define (recall that by $\;\;{\mathbb {E}}\;^s$ we denote the conditional expectation given $\mathcal {F}_s$)

$$\begin{aligned} A_{s,t}=\;\;{\mathbb {E}}\;^s(\mathcal {A}_t-\mathcal {A}_s)=\;\;{\mathbb {E}}\;^s \int _s^t b(B_r)-b(B_{\kappa _n(r)})\,dr. \end{aligned}$$

The stochastic sewing lemma, Proposition 3.2 below, allows one to bound $\mathcal {A}$ through bounds on *A*. Given the preceding field $A_{s,t}$, provided that the conditions (3.8) and (3.9) are satisfied, it is easy to check that the unique adapted process $\mathcal {A}$ constructed in Proposition 3.2 coincides with the one in (3.1). Indeed, the process in (3.1) satisfies (3.10) and (3.11) with $\varepsilon _1=\varepsilon$, $\varepsilon _2=1$, $K_1=\Vert b\Vert _{\mathcal {C}^0}$ and $K_2=0$. Therefore it remains to find $C_1$ and $C_2$. In fact, it is immediate that one can choose $C_2=0$, since $\;\;{\mathbb {E}}\;^s\delta A_{s,u,t}=\;\;{\mathbb {E}}\;^s(A_{s,t}-A_{s,u}-A_{u,t})=0$.

We now claim that one can take $C_1=Nn^{-1/2-\alpha /2+\varepsilon }$ in (3.8). Since $\Vert b(B_r)-b(B_{\kappa _n(r)})\Vert _{L_p(\Omega )}\leqslant \Vert b\Vert _{\mathcal {C}^\alpha }n^{-\alpha /2}$, if $|t-s|\leqslant 2 n^{-1}$, then one easily gets by the conditional Jensen’s inequality

3.2 $$\begin{aligned} \Vert A_{s,t}\Vert _{L_p(\Omega )}\leqslant N |s-t|n^{-\alpha /2}\leqslant N |s-t|^{1/2+\varepsilon }n^{-1/2-\alpha /2+\varepsilon }. \end{aligned}$$

If $|t-s|>2n^{-1}$, let $s'=\kappa _n(s)+2n^{-1}$ be the second gridpoint to the right of *s*. In particular, $r\geqslant s'$ implies $\kappa _n(r)\geqslant s$. Let us furthermore notice that for any $u\geqslant v$ and any bounded measurable function *f*, one has $\;\;{\mathbb {E}}\;^v f(B_u)=\mathcal {P}_{u-v}f(B_v)$, where $\mathcal {P}$ is the standard heat kernel (see (3.22) below for a precise definition). One can then write

3.3 $$\begin{aligned} \Vert A_{s,t}\Vert _{L_p(\Omega )}\leqslant & {} \int _s^{s'}\Vert b(B_r)-b(B_{\kappa _n(r)})\Vert _{L_p(\Omega )}\,dr+\big \Vert \int _{s'}^t\;\;{\mathbb {E}}\;^s b(B_r)-\;\;{\mathbb {E}}\;^s b(B_{\kappa _n(r)})\,dr\big \Vert _{L_p(\Omega )} \nonumber \\\leqslant & {} N n^{-1-\alpha /2}+\int _{s'}^t\Vert (\mathcal {P}_{r-s}-\mathcal {P}_{\kappa _n(r)-s})b\Vert _{\mathcal {C}^0}\,dr \nonumber \\\leqslant & {} N n^{-1-\alpha /2}+N\int _{s'}^t(r-s')^{-1/2+\varepsilon }n^{-1/2-\alpha /2+\varepsilon }\,dr \nonumber \\\leqslant & {} N|t-s|^{1/2+\varepsilon }n^{-1/2-\alpha /2+\varepsilon } \end{aligned}$$

where in the third line we used a well-known estimate for heat kernels, see Proposition 3.7 (ii) with exponents $\beta =0$, $\delta =1/2+\alpha /2-\varepsilon$, and time points $\kappa _n(r)-s$ in place of *s*, $r-s$ in place of *t*. We also used that for $r\geqslant s'$, one has $\kappa _n(r)-s\geqslant r-s'$. By (3.2) and (3.3) we indeed get (3.8) with $C_1=N n^{-1/2-\alpha /2+\varepsilon }$. Applying the stochastic sewing lemma, (3.12) yields

$$\begin{aligned} \Vert \mathcal {A}_t-\mathcal {A}_s\Vert _{L_p(\Omega )}=\big \Vert \int _s^t b(B_r)-b(B_{\kappa _n(r)})\,dr\big \Vert _{L_p(\Omega )}\leqslant N|t-s|^{1/2+\varepsilon }n^{-1/2-\alpha /2+\varepsilon } \end{aligned}$$

for all $0\leqslant s\leqslant t\leqslant T_0$. Here the constant *N* depends on $p,\varepsilon ,\alpha ,d,\Vert b\Vert _{C^\alpha }$, but *not* on $T_0$.

*Step 1.5 (Girsanov transform)*. An easy application of Girsanov’s theorem yields

3.4 $$\begin{aligned} \big \Vert \int _s^t b(X_r^n)-b(X^n_{\kappa _n(r)})\,dr\big \Vert _{L_p(\Omega )}\leqslant N|t-s|^{1/2+\varepsilon }n^{-1/2-\alpha /2+\varepsilon }. \end{aligned}$$

In general (for example, for fractional Brownian motions) the Girsanov transformation can become involved, but for our present example this is completely straightforward.

*Step 2 (“regularization bound”)*. Next, we estimate the quantity

$$\begin{aligned} \mathcal {A}_{T_0}=\int _0^{T_0}b(B_r+\psi _r)-b(B_r+\varphi _r)\,dt \end{aligned}$$

for some adapted processes $\psi ,\varphi$ whose Lipschitz norm is bounded by some constant *K*. As suggested by the above notation, we use the stochastic sewing lemma again, with $A_{s,t}$ defined as

$$\begin{aligned} A_{s,t}=\;\;{\mathbb {E}}\;^s\int _s^t b(B_r+\psi _s)-b(B_r+\varphi _s)\,dr. \end{aligned}$$

We do not give the details of the calculations at this point. It is an instructive exercise to the interested reader to verify that (3.8) and (3.9) are satisfied with $\varepsilon _1=\alpha /2$, $C_1=N[] \psi -\varphi []_{\mathscr {C}^0_p,[0,T_0]}$ and $\varepsilon _2=\alpha /2$, $C_2=N[] \psi -\varphi []_{\mathscr {C}^{1/2}_p,[0,T_0]}$. Here *N* depends on $p,\alpha ,d,K,\Vert b\Vert _{\mathcal {C}^\alpha }$, but *not* on $T_0$. The bound (3.10) is straightforward, with $K_1=\Vert b\Vert _{\mathcal {C}^0}$. Concerning (3.11), one can write

$$\begin{aligned}&|\;\;{\mathbb {E}}\;^s(\mathcal {A}_t-\mathcal {A}_s-A_{s,t})|\leqslant \;\;{\mathbb {E}}\;^s\int _s^t\big |b(B_r+\psi _r)\\&\quad -b(B_r+\psi _s)\big |+\big |b(B_r+\varphi _r)-b(B_r+\varphi _s)\big |\,dr, \end{aligned}$$

and so $K_2=2K\Vert b\Vert _{\mathcal {C}^\alpha }$ does the job. Therefore, by (3.12), we get

$$\begin{aligned} \Vert \mathcal {A}_t-\mathcal {A}_s\Vert _{L_p(\Omega )}= & {} \big \Vert \int _s^t b(B_r+\psi _r)-b(B_r+\varphi _r)\,dr\big \Vert _{L_p(\Omega )} \\\leqslant & {} N |t-s|^{1/2+\alpha /2}[] \psi -\varphi []_{\mathscr {C}^0_p,[0,T_0]}\\&+N |t-s|^{1+\alpha /2}[] \psi -\varphi []_{\mathscr {C}^{1/2}_p,[0,T_0]}. \end{aligned}$$

We will only apply the following simple corollary of this bound: if $\psi _0=\varphi _0$, then

3.5 $$\begin{aligned} \big \Vert \int _s^t b(B_r+\psi _s)-b(B_r+\varphi _s)\,dr\big \Vert _{L_p(\Omega )}\leqslant N |t-s|^{1/2+\alpha /2}[] \psi -\varphi []_{\mathscr {C}^{1/2}_p,[0,T_0]}.\nonumber \\ \end{aligned}$$

*Step 3 (“Buckling”)* Let $\psi$ and $\psi ^n$ be the drift component of *X* and $X^n$, respectively:

$$\begin{aligned} \psi _t=x_0+\int _0^t b(X_r)\,dr,\qquad \psi ^n_t=x_0+\int _0^t b(X^n_{\kappa _n(r)})\,dr. \end{aligned}$$

We apply (3.4) and (3.5) with $\varphi =\psi ^n$, to get

$$\begin{aligned} \Vert (\psi -\psi ^n)_t-(\psi -\psi ^n)_s\Vert _{L_p(\Omega )}\leqslant & {} Nn^{-1/2-\alpha /2+\varepsilon }|t-s|^{1/2+\varepsilon } \\&+ N |t-s|^{1/2+\alpha /2}[] \psi -\psi ^n []_{\mathscr {C}^{1/2}_p,[0,T_0]}. \end{aligned}$$

Dividing by $|t-s|^{1/2}$ and take supremum over $0\leqslant s\leqslant t\leqslant T_0$, one gets

$$\begin{aligned}{}[] \psi -\psi ^n []_{\mathscr {C}^{1/2}_p,[0,T_0]}\leqslant Nn^{-1/2-\alpha /2+\varepsilon } +NT_0^{\alpha /2} [] \psi -\psi ^n []_{\mathscr {C}^{1/2}_p,[0,T_0]}. \end{aligned}$$

Since so far *N* does not depend on $T_0$, one can choose $T_0$ sufficiently small so that $NT_0^{\alpha /2}\leqslant 1/2$. This yields the desired bound

$$\begin{aligned}{}[] X-X^n []_{\mathscr {C}^{1/2}_p,[0,T_0]}=[] \psi -\psi ^n []_{\mathscr {C}^{1/2}_p,[0,T_0]} \leqslant Nn^{-1/2-\alpha /2+\varepsilon }. \end{aligned}$$

$$\square $$

Let us point out that the rate of convergence is determined by only the first step. Also, the second step is similar in spirit to the ‘averaging bounds’ appearing in sewing-based uniqueness proofs for SDEs (see e.g. [8, 27]).

In the proof of Theorem 2.1, the more difficult part will be the regularization bound. Applying only the stochastic sewing lemma of Lê apparently does not lead to an optimal result for $H>1/2$. Therefore at some point one has to move from almost sure bounds (which are similar to [8]) to $L_p$ bounds. This requires an extension of the Davie’s moment bound [9, Proposition 2.1] to the case of the fractional Brownian motion. This is done in Lemma 4.3 using the new stochastic sewing lemma (Theorem 3.3).

In contrast, for Theorem 2.7 establishing the quadrature bound will be more difficult. In the above arguments, the heat kernel bounds have to be replaced by estimates on the transition densities of the Euler–Maruyama scheme. These bounds are established via Malliavin calculus, this is the content of Sect. 5.

### Sewing lemmas

As mentioned above, the proof strategy relies on the sewing and stochastic sewing lemmas. For the convenience of the reader, we recall them here. The first two lemmas are well-known, the third one is new.

We define for $0\leqslant S\leqslant T\leqslant 1$ the set $[S,T]_\leqslant :=\{(s,t):\,S\leqslant s\leqslant t\leqslant T\}$. If $A_{\cdot ,\cdot }$ is a function $[S,T]_\leqslant \rightarrow \mathbb {R}^d$, then for $s\leqslant u\leqslant t$ we put $\delta A_{s,u,t}:=A_{s,t}-A_{s,u}-A_{u,t}$. The first statement is the sewing lemma of Gubinelli.

#### Proposition 3.1

[14, Lemma 2.1], [19, Proposition 1] Let $0\leqslant S\leqslant T\leqslant 1$ and let $A_{\cdot ,\cdot }$ be a continuous function from $[S,T]_\leqslant$ to $\mathbb {R}^d$. Suppose that for some $\varepsilon >0$ and $C>0$ the bound

3.6 $$\begin{aligned} |\delta A_{s,u,t}| \leqslant C |t-s|^{1+\varepsilon } \end{aligned}$$

holds for all $S\leqslant s\leqslant u\leqslant t\leqslant T$. Then there exists a unique function $\mathcal {A}:[S,T]\rightarrow \mathbb {R}^d$ such that $\mathcal {A}_S=0$ and the following bound holds for some constant $K>0$:

3.7 $$\begin{aligned} |\mathcal {A}_t -\mathcal {A}_s-A_{s,t}|\leqslant K |t-s|^{1+\varepsilon }, \quad (s,t)\in [S,T]_\leqslant . \end{aligned}$$

Moreover, there exists a constant $K_0$ depending only on $\varepsilon$, *d* such that $\mathcal {A}$ in fact satisfies the above bound with $K\leqslant K_0 C$.

The next statement is the stochastic extension of the above result obtained by Lê. Recall that for any $s\geqslant 0$ we are using the convention $\;\;{\mathbb {E}}\;^s[...]:=\;\;{\mathbb {E}}\;[...|\mathcal {F}_s]$.

#### Proposition 3.2

[27, Theorem 2.4]. Let $p\geqslant 2$, $0\leqslant S\leqslant T\leqslant 1$ and let $A_{\cdot ,\cdot }$ be a function $[S,T]_\leqslant \rightarrow L_p(\Omega ,\mathbb {R}^d)$ such that for any $(s,t)\in [S,T]_\leqslant$ the random vector $A_{s,t}$ is $\mathcal {F}_t$-measurable. Suppose that for some $\varepsilon _1,\varepsilon _2>0$ and $C_1,C_2$ the bounds

3.8 $$\begin{aligned} \Vert A_{s,t}\Vert _{L_p(\Omega )}\leqslant & {} C_1|t-s|^{1/2+\varepsilon _1}, \end{aligned}$$

3.9 $$\begin{aligned} \Vert \;\;{\mathbb {E}}\;^s\delta A_{s,u,t}\Vert _{L_p(\Omega )}\leqslant & {} C_2 |t-s|^{1+\varepsilon _2} \end{aligned}$$

hold for all $S\leqslant s\leqslant u\leqslant t\leqslant T$. Then there exists a unique (up to modification) $\mathbb {F}$-adapted process $\mathcal {A}:[S,T]\rightarrow L_p(\Omega ,\mathbb {R}^d)$ such that $\mathcal {A}_S=0$ and the following bounds hold for some constants $K_1,K_2>0$:

3.10 $$\begin{aligned} \Vert \mathcal {A}_t -\mathcal {A}_s-A_{s,t}\Vert _{L_p(\Omega )}&\leqslant K_1 |t-s|^{1/2+\varepsilon _1}+K_2 |t-s|^{1+\varepsilon _2},\quad (s,t)\in [S,T]_\leqslant , \end{aligned}$$

3.11 $$\begin{aligned} \Vert \;\;{\mathbb {E}}\;^s\big (\mathcal {A}_t -\mathcal {A}_s-A_{s,t}\big )\Vert _{L_p(\Omega )}&\leqslant K_2|t-s|^{1+\varepsilon _2},\quad (s,t)\in [S,T]_\leqslant . \end{aligned}$$

Moreover, there exists a constant *K* depending only on $\varepsilon _1,\varepsilon _2$, *d* such that $\mathcal {A}$ satisfies the bound

3.12 $$\begin{aligned} \Vert \mathcal {A}_t-\mathcal {A}_s\Vert _{L_p(\Omega )} \leqslant KpC_1 |t-s|^{1/2+\varepsilon _1}+KpC_2 |t-s|^{1+\varepsilon _2},\quad (s,t)\in [S,T].\nonumber \\ \end{aligned}$$

The final statement of this section is new. It provides bounds on $\Vert \mathcal {A}_s-\mathcal {A}_t\Vert _{L_p(\Omega )}$ with the correct dependence on *p*: namely these bounds are of order $\sqrt{p}$, rather than *p* as in (3.12). This will be crucial for the proof of Theorem 2.1; in particular, this would allow to extend the corresponding Davie bound [9, Proposition 2.1] to the case of fractional Brownian motion. The price to pay though is that the assumptions of this theorem are more restrictive than the corresponding assumptions of [27, Theorem 2.4].

#### Theorem 3.3

Fix $0\leqslant S\leqslant T\leqslant 1$. Let $(\mathcal {A}_t)_{t\in [S,T]}$ be an $\mathbb {F}$–adapted process with values in $\mathbb {R}^d$. For $(s,t)\in [S,T]_\leqslant$ we will write $\mathcal {A}_{s, t}:=\mathcal {A}_t-\mathcal {A}_s$. Let $p\geqslant 2$. Suppose that for some $m\geqslant 2$, $\varepsilon _1>0$, $\varepsilon _2\geqslant 0$, $\varepsilon _3\geqslant 0$, and $C_1,C_2, C_3>0$ the bounds

3.13 $$\begin{aligned}&\Vert \mathcal {A}_{s,t}\Vert _{L_{p\vee m}(\Omega )}\leqslant C_1 |t-s|^{1/2+\varepsilon _1}\end{aligned}$$

3.14 $$\begin{aligned}&\Vert \;\;{\mathbb {E}}\;^s\mathcal {A}_{u,t}-\;\;{\mathbb {E}}\;^u\mathcal {A}_{u,t}\Vert _{L_m(\Omega )}\leqslant C_1 |u-s|^{1/m+\varepsilon _1}\end{aligned}$$

3.15 $$\begin{aligned}&\Vert \;\;{\mathbb {E}}\;^s\mathcal {A}_{s,t}\Vert _{L_p(\Omega )}\leqslant C_2 |t-s|^{\varepsilon _2}\end{aligned}$$

3.16 $$\begin{aligned}&\bigl \Vert \;\;{\mathbb {E}}\;^s[(\;\;{\mathbb {E}}\;^s\mathcal {A}_{u,t}-\;\;{\mathbb {E}}\;^u\mathcal {A}_{u,t})^2]\bigr \Vert _{L_{p/2}(\Omega )}\leqslant C_3 |u-s||t-s|^{\varepsilon _3} \end{aligned}$$

hold for all $S\leqslant s\leqslant u\leqslant t\leqslant T$. Then there exist a universal constant $K=K(d,\varepsilon _2,\varepsilon _3)>0$ which does not depend on *p*, $C_j$, such that

3.17 $$\begin{aligned} \Vert \mathcal {A}_{t}-\mathcal {A}_s\Vert _{L_p(\Omega )}\leqslant C_2K|t-s|^{\varepsilon _2}+K\sqrt{p}\,C_3^{1/2}|t-s|^{1/2+\varepsilon _3/2}. \end{aligned}$$

#### Remark 3.4

Note that the right–hand side of bound (3.17) does not depend on $C_1$.

#### Remark 3.5

Let us recall that the proof of stochastic sewing lemma in [27] requires to apply the BDG inequality infinitely many times but each time to a discrete-time martingale, thus yielding a constant *p* in the right–hand side of bound (3.12). In our proof we apply the BDG inequality only once, but to a continuous time martingale. This allows to get a better constant (namely $\sqrt{p}$ instead of *p*), since the constant in the BDG inequality for the continuous-time martingales is better than in the BDG inequality for general martingales.

#### Proof of Theorem 3.3

This proof is inspired by the ideas of [3, proof of Proposition 3.2] and [8, proof of Theorem 4.3]. For the sake of brevity, in this proof we will write $L_p$ for $L_p(\Omega )$. Fix $s,t\in [S,T]_{\leqslant }$ and for $i\in \{1,\ldots ,d\}$ consider a martingale $M^{i}=(M^i_r)_{r\in [s,t]}$, where

$$\begin{aligned} M^i_r:=\;\;{\mathbb {E}}\;^r[\mathcal {A}^i_{s,t}],\quad r\in [s,t]. \end{aligned}$$

We will frequently use the following inequality. For $s\leqslant u\leqslant v\leqslant t$ one has

3.18 $$\begin{aligned} |M^i_u-M^i_v|\leqslant |\mathcal {A}^i_{u,v}|+|\;\;{\mathbb {E}}\;^u \mathcal {A}^i_{u,v}|+|\;\;{\mathbb {E}}\;^u\mathcal {A}^i_{v,t}-\;\;{\mathbb {E}}\;^v\mathcal {A}^i_{v,t}|. \end{aligned}$$

We begin by observing that

3.19 $$\begin{aligned} \Vert \mathcal {A}_{s,t}\Vert _{L_p(\Omega )}&\leqslant \sum _{i=1}^d\Vert \mathcal {A}^{i}_{s,t}\Vert _{L_p(\Omega )}=\sum _{i=1}^d\Vert M^{i}_{t}\Vert _{L_p(\Omega )}\nonumber \\&\leqslant \sum _{i=1}^d\Vert M^{i}_{s}\Vert _{L_p(\Omega )}+\sum _{i=1}^d\Vert M^{i}_{t}-M^{i}_{s}\Vert _{L_p(\Omega )}\nonumber \\&=:\sum _{i=1}^dI_1^{i}+\sum _{i=1}^dI_2^{i}. \end{aligned}$$

The first term in (3.19) is easy to bound. By assumption (3.15) we have

3.20 $$\begin{aligned} I_1^{i}=\Vert \;\;{\mathbb {E}}\;^{s} \mathcal {A}_{s,t}^{i}\Vert _{L_p(\Omega )}\leqslant C_2 |t-s|^{\varepsilon _2}. \end{aligned}$$

To estimate $I_2^i$ we first observe that for each $i=1,\dots ,d$ the martingale $M^{i}$ is continuous. Indeed, for any $s\leqslant u\leqslant v\leqslant t$ we have using (3.18), (3.13), and (3.14)

$$\begin{aligned} \Vert M^{i}_u-M^{i}_v\Vert _{L_m}&\leqslant 2\Vert \mathcal {A}^{i}_{u,v}\Vert _{L_m}+\Vert \;\;{\mathbb {E}}\;^u\mathcal {A}^{i}_{v,t}-\;\;{\mathbb {E}}\;^v\mathcal {A}^{i}_{v,t}\Vert _{L_m}\\&\leqslant 3C_1|u-v|^{1/m+\varepsilon _1}. \end{aligned}$$

Therefore, the Kolmogorov continuity theorem implies that the martingale $M^{i}$ is continuous. Hence, its quadratic variation $[M^{i}]$ equals its predictable quadratic variation $\langle M^{i}\rangle$ [24, Theorem I.4.52]. Thus, applying a version of the Burkholder–Davis–Gundy inequality with a precise bound on the constant [7, Proposition 4.2], we get that there exists a universal constant $N>0$ such that

3.21 $$\begin{aligned} \Vert M^{i}_t-M^{i}_s\Vert _{L_p(\Omega )}\leqslant N\sqrt{p}\,\Vert \langle M^{i}\rangle _t\Vert _{L_{p/2}}^{1/2}. \end{aligned}$$

For $n\in \mathbb {N}$, $j\in \{1,\ldots ,n\}$ put $t^n_j:=s+(t-s)j/n$. Then, it follows from [23, Theorem 2] that $\sum _{j=0}^{n-1}\;\;{\mathbb {E}}\;^{t_j^n}[(M^i_{t^n_{j+1}}-M^i_{t^n_j})^2]$ converges to $\langle M^{i}\rangle _t$ in $L_1(\Omega )$. In particular, a subsequence indexed over $n_k$ converges almost surely. Therefore, applying Fatou’s lemma, Minkowski’s inequality, (3.18) and using the assumptions of the theorem, we deduce

$$\begin{aligned} \Vert \langle M^{i}\rangle _t\Vert _{L_{p/2}}&=\Bigl \Vert \lim _{k\rightarrow \infty }\sum _{j=0}^{n_k-1}\;\;{\mathbb {E}}\;^{t_j^{n_k}}(M^{i}_{t^{n_k}_{j+1}}-M^{i}_{t^{n_k}_j})^2\Bigr \Vert _{L_{p/2}} \\&\leqslant \liminf _{k \rightarrow \infty }\sum _{j=0}^{n_k-1}\bigl \Vert \;\;{\mathbb {E}}\;^{t_j^{n_k}}(M^{i}_{t^{n_k}_{j+1}}-M^{i}_{t^{n_k}_j})^2\bigr \Vert _{L_{p/2}}\\&\leqslant 3\lim _{k\rightarrow \infty }\sum _{j=0}^{n_k-1}\bigl (2\Vert \mathcal {A}^{i}_{t^{n_k}_{j},t^{n_k}_{j+1}}\Vert _{L_p(\Omega )}^2+\Vert \;\;{\mathbb {E}}\;^{t_j^{n_k}}(\;\;{\mathbb {E}}\;^{t_j^{n_k}}\mathcal {A}^{i}_{t^{n_k}_{j+1},t}-\;\;{\mathbb {E}}\;^{t_{j+1}^{n_k}}\mathcal {A}^{i}_{t^{n_k}_{j+1},t})^2\bigr \Vert _{L_{p/2}}\bigr )\\&\leqslant \lim _{k\rightarrow \infty }6C_1^2T^{1+2\varepsilon _1}n_k^{-2\varepsilon _1}+3\lim _{k\rightarrow \infty }C_3|t-s|^{1+\varepsilon _3}n_k^{-1-\varepsilon _3}\sum _{j=0}^{n_k-1}(n_k-j)^{\varepsilon _3}\\&\leqslant N C_3|t-s|^{1+\varepsilon _3}. \end{aligned}$$

Substituting this into (3.21) and combining this with (3.19) and (3.20), we obtain (3.17). $\square$

### Some useful estimates

In this section we establish a number of useful technical bounds related to Gaussian kernels. Their proofs are mostly standard, however we were not able to find them in the literature. Therefore for the sake of completeness, we provide the proofs of these results in the “Appendix A”.

Fix an arbitrary $H\in (0,1)$. Define

$$\begin{aligned} c(s,t):=\sqrt{(2H)^{-1}}|t-s|^{H},\quad 0\leqslant s\leqslant t\leqslant 1. \end{aligned}$$

Let $p_t$, $t>0$, be the density of a *d*-dimensional vector with independent Gaussian components each of mean zero and variance *t*:

3.22 $$\begin{aligned} p_t(x)=\frac{1}{(2\pi t)^{d/2}}\exp \Bigl (-\frac{|x|^2}{2t}\Bigr ),\quad x\in \mathbb {R}^d. \end{aligned}$$

For a measurable function $f:\mathbb {R}^d\rightarrow \mathbb {R}$ we write $\mathcal {P}_t f:=p_t*f$, and occasionally we denote by $p_0$ the Dirac delta function.

Our first statement provides a number of technical bounds related to the fractional Brownian motion. Its proof is placed in the “Appendix A”.

#### Proposition 3.6

Let $p\geqslant 1$. The process $B^H$ has the following properties:

(i): $\Vert B^H_t-B^H_s\Vert _{L_p(\Omega )}= N |t-s|^H$, for all $0\leqslant s\leqslant t\leqslant 1$, with $N=N(p,d,H)$;
(ii): for all $0\leqslant s\leqslant u\leqslant t\leqslant 1$, $i=1,\ldots ,d$, the random variable $\;\;{\mathbb {E}}\;^sB_t^{H,i}-\;\;{\mathbb {E}}\;^uB_t^{H,i}$ is independent of $\mathcal {F}^s$; furthermore, this random variable is Gaussian with mean 0 and variance
  3.23 $$\begin{aligned} \;\;{\mathbb {E}}\;(\;\;{\mathbb {E}}\;^sB_t^{H,i}-\;\;{\mathbb {E}}\;^uB_t^{H,i})^2= c^2(s,t)-c^2(u,t)=:v(s,u,t); \end{aligned}$$
(iii): $\;\;{\mathbb {E}}\;^s f(B^H_t)=\mathcal {P}_{c^2(s,t)}f(\;\;{\mathbb {E}}\;^sB^H_t)$, for all $0\leqslant s\leqslant t\leqslant 1$;
(iv): $|c^2(s,t)-c^2(s,u)|\leqslant N|t-u||t-s|^{2H-1}$, for all $0\leqslant s\leqslant u\leqslant t$ such that $|t-u|\leqslant |u-s|$, with $N=N(H)$;
(v): $\Vert \;\;{\mathbb {E}}\;^sB^H_t-\;\;{\mathbb {E}}\;^sB^H_u\Vert _{L_p(\Omega )}\leqslant N|t-u||t-s|^{H-1}$, for all $0\leqslant s\leqslant u\leqslant t$ such that $|t-u|\leqslant |u-s|$, with $N=N(p,d,H)$;

The next statement gives the heat kernel bounds which are necessary for the proofs of the main results. Its proof is also placed in the “Appendix A”. Recall the definition of the function *v* in (3.23).

#### Proposition 3.7

Let $f\in \mathcal {C}^\alpha$, $\alpha \leqslant 1$ and $\beta \in [0,1]$. The following hold:

(i): There exists $N=N(d, \alpha , \beta )$ such that
  $$\begin{aligned} \Vert \mathcal {P}_tf\Vert _{\mathcal {C}^\beta (\mathbb {R}^d)}\leqslant N t^{\frac{(\alpha -\beta )\wedge 0}{2}} \Vert f\Vert _{\mathcal {C}^\alpha (\mathbb {R}^d)}, \end{aligned}$$
  for all $$t\in (0,1]$$.
(ii): For all $\delta \in (0,1]$ with $\delta \geqslant \frac{\alpha }{2}-\frac{\beta }{2}$, there exists $N=N(d, \alpha , \beta , \delta )$ such that
  $$\begin{aligned} \Vert \mathcal {P}_tf-\mathcal {P}_sf\Vert _{\mathcal {C}^\beta (\mathbb {R}^d)}\leqslant N \Vert f\Vert _{\mathcal {C}^{\alpha }(\mathbb {R}^d)} s^{\frac{\alpha }{2}-\frac{\beta }{2}-\delta }(t-s)^{\delta }, \end{aligned}$$
  for all $$0\leqslant s\leqslant t \leqslant 1$$.
(iii): For all $H\in (0,1)$, there exists $N=N(d,\alpha ,\beta , H)$ such that
  $$\begin{aligned} \Vert \mathcal {P}_{c^2(s,t)}f-\mathcal {P}_{c^2(u,t)}f\Vert _{\mathcal {C}^\beta (\mathbb {R}^d)}\leqslant N\Vert f\Vert _{\mathcal {C}^\alpha (\mathbb {R}^d)}(u-s)^{\frac{1}{2}}(t-u)^{(H(\alpha -\beta )-\frac{1}{2})\wedge 0}, \end{aligned}$$
  for all $$0<s\leqslant u \leqslant t\leqslant 1$$.
(iv): For all $H\in (0,1)$, $p\geqslant 2$, there exists $N=N(d,\alpha ,H,p)$ such that
  $$\begin{aligned} \Vert \mathcal {P}_{c^2(u,t)}f(x)-\mathcal {P}_{c^2(u,t)}f(x+\xi )\Vert _{L_p(\Omega )} \leqslant N\Vert f\Vert _{\mathcal {C}^\alpha } (u-s)^{\frac{1}{2}}(t-u)^{(H\alpha -\frac{1}{2})\wedge 0}; \end{aligned}$$
  for all $x\in \mathbb {R}^d$, $0<s\leqslant u\leqslant t\leqslant 1$ and all random vectors $\xi$ whose components are independent, $\mathcal {N}(0,v(s,u,t))$ random variables.

Our next statement relates to the properties of Hölder norms. Its proof can be found in “Appendix A”.

#### Proposition 3.8

Let $\alpha \in \mathbb {R}$, $f\in \mathcal {C}^\alpha (\mathbb {R}^d,\mathbb {R}^k)$, $\delta \in [0,1]$. Then there exists $N=N(\alpha ,\delta ,d , k)$ such that for any $x\in \mathbb {R}^d$

$$\begin{aligned} \Vert f(x+\cdot )-f(\cdot )\Vert _{\mathcal {C}^{\alpha -\delta }}\leqslant N|x|^\delta \Vert f\Vert _{\mathcal {C}^\alpha }. \end{aligned}$$

Finally, we will also need the following integral bounds. They follow immediately from a direct calculation.

#### Proposition 3.9

(i): Let $a,b>-1$, $t>0$. Then for some $N=N(a,b)$ one has
  3.24 $$\begin{aligned} \int _0^t(t-r)^ar^b\,dr=N t^{a+b+1}. \end{aligned}$$
(ii): Let $a>-2$, $b<1$, $t>0$. Then for some $N=N(a,b)$ one has
  3.25 $$\begin{aligned} \Big |\int _0^t(t-r)^{a}(t^{b}r^{-b}-1)\,dr\Big |=N t^{a+1}. \end{aligned}$$

### Girsanov theorem for fractional Brownian motion

One of the tools which are important for the proof of Theorem 2.1 is the Girsanov theorem for fractional Brownian motion [12, Theorem 4.9], [32, Theorem 2]. We will frequently use the following technical corollary of this theorem. For the convenience of the reader we put its proof into “Appendix B”.

#### Proposition 3.10

Let $u:\Omega \times [0,1]\rightarrow \mathbb {R}^d$ be an $\mathbb {F}$–adapted process such that with a constant $M>0$ we have

3.26 $$\begin{aligned} \Vert u \Vert _{L_\infty (0,1)} \leqslant M, \end{aligned}$$

almost surely. Further, assume that one of the following holds:

(i): $$H\leqslant 1/2$$;

or

(ii): $H>1/2$ and there exists a random variable $\xi$ such that
  3.27 $$\begin{aligned} \int _0^1\Bigl (\int _0^t\frac{(t/s)^{H-1/2}|u_t-u_s|}{(t-s)^{H+1/2}}\,ds\Bigr )^2\,dt\leqslant \xi \end{aligned}$$
  and $\;\;{\mathbb {E}}\;\exp (\lambda \xi )<\infty$ for any $\lambda >0$.

Then there exists a probability measure ${\widetilde{\mathbb {P}}}$ which is equivalent to $\mathbb {P}$ such that the process ${\widetilde{B}}^H:=B^H+\int _0^\cdot u_s\,ds$ is a fractional Brownain motion with Hurst parameter *H* under ${\widetilde{\mathbb {P}}}$. Furthermore for any $\lambda >0$ we have

3.28 $$\begin{aligned} \;\;{\mathbb {E}}\;\Bigl (\frac{d \mathbb {P}}{d{\widetilde{\mathbb {P}}}}\Bigr )^\lambda \leqslant {\left\{ \begin{array}{ll} \exp (\lambda ^2 NM^2)\qquad \qquad \qquad \qquad \text {if }H\in (0,1/2]\\ \exp (\lambda ^2 NM^2)\;\;{\mathbb {E}}\;[\exp (\lambda N\xi )]\qquad \text {if }H\in (1/2,1) \end{array}\right. } <\infty , \end{aligned}$$

where $$N=N(H)$$.

In order to simplify the calculation of the integral in (3.27), we provide the following technical but useful lemma. Since the proof is purely technical, we put its proof in the “Appendix B”.

#### Lemma 3.11

Let $H\in (1/2,1)$ and let $\rho \in (H-1/2,1]$. Then there exists a constant $N=N(H,\rho )$, such that for any function $f\in \mathcal {C}^\rho ([0,1],\mathbb {R}^d)$ and any $n\in \mathbb {N}$ one has

3.29 $$\begin{aligned}&\int _0^1\Bigl (\int _0^t\frac{(t/s)^{H-1/2} |f_{\kappa _n(t)}-f_{\kappa _n(s)}|}{(t-s)^{H+1/2}}\,ds\Bigr )^2\,dt\leqslant N[f]_{\mathcal {C}^\rho }^2. \end{aligned}$$

3.30 $$\begin{aligned}&\int _0^1\Bigl (\int _0^t\frac{(t/s)^{H-1/2} |f_{t}-f_{s}|}{(t-s)^{H+1/2}}\,ds\Bigr )^2\,dt\leqslant N[f]_{\mathcal {C}^\rho }^2. \end{aligned}$$

## Additive fractional noise

In this section we provide the proof of Theorem 2.1. We follow the strategy outlined on Sect. 3.1: In Sects. 4.1 and 4.2 we prove the quadrature bound and the regularization bound, respectively. Based on these bounds, the proof of the theorem is placed in Sect. 4.3.

### Quadrature estimates

The goal of this subsection is to prove the quadrature bound (4.7). The proof consists of two steps. First, in Lemma 4.1 we prove this bound for the case of fractional Brownian motion; then we extend this result to the process *X* by applying the Girsanov theorem.

Recall the definition of functions $\kappa _n$ in (1.5) and $\gamma$ in (2.4).

#### Lemma 4.1

Let $H\in (0,1)$, $\alpha \in [0,1]$, $p>0$, and take $\varepsilon \in (0,1/2]$. Then for all $f\in \mathcal {C}^\alpha$, $0\leqslant s\leqslant t\leqslant 1$, $n\in \mathbb {N}$, one has the bound

4.1 $$\begin{aligned} \Bigl \Vert \int _s^t (f(B^H_r)-f(B^H_{\kappa _n(r)}))\, dr\Bigr \Vert _{L_p(\Omega )} \leqslant N\Vert f\Vert _{\mathcal {C}^\alpha } n^{-\gamma (\alpha , H)+\varepsilon }|t-s|^{1/2+\varepsilon } , \end{aligned}$$

with some $$N=N(p, d,\alpha ,\varepsilon , H)$$.

#### Proof

It suffices to prove the bound for $p\geqslant 2$. Define for $0\leqslant s\leqslant t\leqslant 1$

$$\begin{aligned} A_{s,t}:=\;\;{\mathbb {E}}\;^s \int _s^t (f(B^H_r)-f(B^H_{\kappa _n(r)}))\, dr. \end{aligned}$$

Then, clearly, for any $0\leqslant s\leqslant u\leqslant t\leqslant 1$

$$\begin{aligned} \delta A_{s,u,t}:&=A_{s,t}-A_{s,u}-A_{u,t}\\&=\;\;{\mathbb {E}}\;^s \int _u^t (f(B^H_r)-f(B^H_{\kappa _n(r)}))\, dr-\;\;{\mathbb {E}}\;^u \int _u^t(f(B^H_r)-f(B^H_{\kappa _n(r)}))\, dr. \end{aligned}$$

Let us check that all the conditions of the stochastic sewing lemma (Proposition 3.2) are satisfied. Note that

$$\begin{aligned} \;\;{\mathbb {E}}\;^s \delta A_{s,u,t}=0, \end{aligned}$$

and so condition (3.9) trivially holds, with $C_2=0$. To establish (3.8), let $s \in [k/n, (k+1)/n)$ for some $k \in \{0,\ldots ,n-1\}$. Suppose first that $t \in [(k+4)/n, 1]$. We write

4.2 $$\begin{aligned} |A_{s,t}|\leqslant \Big (\int _s^{(k+4)/n} +\int _{(k+4)/n}^t\Big ) |\;\;{\mathbb {E}}\;^s \big ( f(B^H_r)-f(B^H_{\kappa _n(r)})\big )|\, dr=:I_1+I_2. \end{aligned}$$

The bound for $I_1$ is straightforward: by conditional Jensen’s inequality, the definition of $\mathcal {C}^\alpha$ norm, and Proposition  3.6 (i) we have

4.3 $$\begin{aligned} \Vert I_1\Vert _{L_p(\Omega )}&\leqslant \int _s^{(k+4)/n} \Vert f(B^H_r)-f(B^H_{\kappa _n(r)}) \Vert _{L_p(\Omega )} \, dr \nonumber \\&\leqslant N \Vert f\Vert _{\mathcal {C}^\alpha }n^{-1-\alpha H} \leqslant N \Vert f\Vert _{\mathcal {C}^\alpha } n^{-\gamma +\varepsilon }|t-s|^{1/2+\varepsilon }, \end{aligned}$$

where the last inequality follows from the fact that $n^{-1}\leqslant |t-s|$.

Now let us estimate $I_2$. Using Proposition 3.6 (iii), we derive

4.4 $$\begin{aligned} I_2\leqslant&\int _{(k+4)/n}^t|\mathcal {P}_{c^2(s,r)}f(\;\;{\mathbb {E}}\;^sB^H_r)-\mathcal {P}_{c^2(s,\kappa _n(r))}f(\;\;{\mathbb {E}}\;^sB^H_r)|\,dr \nonumber \\&\quad +\int _{(k+4)/n}^t |\mathcal {P}_{c^2(s,\kappa _n(r))}f(\;\;{\mathbb {E}}\;^sB^H_r)-\mathcal {P}_{c^2(s,\kappa _n(r))}f(\;\;{\mathbb {E}}\;^sB^H_{\kappa _n(r)})|\,dr\nonumber \\ =:&I_{21}+I_{22}. \end{aligned}$$

To bound $I_{21}$, we apply Proposition 3.7 (ii) with $\beta =0$, $\delta =1$ and Proposition 3.6 (iv). We get

4.5 $$\begin{aligned} \Vert I_{21}\Vert _{L_p(\Omega )}&\leqslant N\Vert f\Vert _{\mathcal {C}^\alpha } \int _{(k+4)/n}^t\big (c^2(s,r)-c^2(s,\kappa _n(r))\big )c^{\alpha -2}(s,\kappa _n(r))\,dr \nonumber \\&\leqslant N\Vert f\Vert _{\mathcal {C}^\alpha }\int _{(k+4)/n}^t n^{-1}|r-s|^{2H-1}|r-s|^{H(\alpha -2)}\,dr \nonumber \\&\leqslant N\Vert f\Vert _{\mathcal {C}^\alpha }n^{-1}\int _s^t |r-s|^{-1+\alpha H}\,dr \nonumber \\&\leqslant N\Vert f\Vert _{\mathcal {C}^\alpha } n^{-1}|t-s|^{\alpha H}. \end{aligned}$$

To deal with $I_{22}$, we use Proposition 3.7 (i) with $\beta =1$ and Proposition 3.6 (v). We deduce

4.6 $$\begin{aligned} \Vert I_{22}\Vert _{L_p(\Omega )}&\leqslant N\Vert f\Vert _{\mathcal {C}^\alpha }\int _{(k+4)/n}^t \Vert \;\;{\mathbb {E}}\;^sB^H_r-\;\;{\mathbb {E}}\;^sB^H_{\kappa _n(r)}\Vert _{L_p(\Omega )}c^{\alpha -1}(s,\kappa _n(r))\,dr \nonumber \\&\leqslant N\Vert f\Vert _{\mathcal {C}^\alpha }\int _{(k+4)/n}^t n^{-1}|r-s|^{H-1}|r-s|^{-H(1-\alpha )}\,dr \nonumber \\&\leqslant N\Vert f\Vert _{\mathcal {C}^\alpha }n^{-1}|t-s|^{\alpha H}, \end{aligned}$$

where in the second inequality we have also used that $\kappa _n(r)-s\geqslant (r-s)/2$. Combining (4.5) and (4.6), and taking again into account that $n^{-1}\leqslant |t-s|$, we get

$$\begin{aligned} \Vert I_2\Vert _{L_p(\Omega )} \leqslant N\Vert f\Vert _{\mathcal {C}^\alpha } n^{-\gamma +\varepsilon }|t-s|^{1/2+\varepsilon }. \end{aligned}$$

Recalling (4.3), we finally conclude

$$\begin{aligned} \Vert A_{s,t}\Vert _{L_p(\Omega )}\leqslant N \Vert f\Vert _{\mathcal {C}^\alpha } n^{-\gamma +\varepsilon }|t-s|^{1/2+\varepsilon }. \end{aligned}$$

It remains to show the same bound for $t \in (s, (k+4)/n]$. However this is almost straightforward. We write

$$\begin{aligned} \Vert A_{s,t}\Vert _{L_p(\Omega )}&\leqslant \int _s^t \Vert f(B^H_r)-f(B^H_{\kappa _n(r)}) \Vert _{L_p(\Omega )} \, dr \\&\leqslant N \Vert f\Vert _{\mathcal {C}^\alpha } n^{-\alpha H}|t-s| \leqslant N \Vert f\Vert _{\mathcal {C}^\alpha } n^{-\gamma +\varepsilon }|t-s|^{1/2+\varepsilon }, \end{aligned}$$

where the last inequality uses that in this case $|t-s|\leqslant 4 n^{-1}$. Thus, (3.8) holds, with $C_1:=N \Vert f\Vert _{\mathcal {C}^\alpha } n^{-\gamma +\varepsilon }$, $\varepsilon _1:=\varepsilon$.

Thus all the conditions of the stochastic sewing lemma are satisfied. The process

$$\begin{aligned} {\tilde{\mathcal {A}}}_t:=\int _0^t (f(B^H_r)-f(B^H_{\kappa _n(r)}))\,dr \end{aligned}$$

is also $\mathbb {F}$-adapted, satisfies (3.11) trivially (the left-hand side is 0), and

$$\begin{aligned} \Vert {\tilde{{\mathcal {A}}}}_t-{\tilde{{\mathcal {A}}}}_s-A_{s,t}\Vert _{L_p(\Omega )} \leqslant \Vert f\Vert _{\mathcal {C}^0} |t-s|\leqslant N |t-s|^{1/2+\varepsilon }, \end{aligned}$$

which shows that it also satisfies (3.10). Therefore by uniqueness $\mathcal {A}_t={\tilde{\mathcal {A}}}_t$. The bound (3.12) then yields precisely (4.1). $\square$

#### Lemma 4.2

Let $H\in (0,1)$, $\alpha \in [0,1]$ such that $\alpha >1-1/(2H)$, $p>0$, $\varepsilon \in (0,1/2]$. Let $b\in \mathcal {C}^\alpha$ and $X^n$ be the solution of (1.4). Then for all $f\in \mathcal {C}^\alpha$, $0\leqslant s\leqslant t\leqslant 1$, $n\in \mathbb {N}$, one has the bound

4.7 $$\begin{aligned} \Bigl \Vert \int _s^t (f(X_r^n)-f(X^n_{\kappa _n(r)}))\, dr\Bigr \Vert _{L_p(\Omega )} \leqslant N\Vert f\Vert _{\mathcal {C}^\alpha } |t-s|^{1/2+\varepsilon } n^{-\gamma +\varepsilon } \end{aligned}$$

with some $$N=N(\Vert b\Vert _{\mathcal {C}^\alpha },p, d,\alpha ,\varepsilon , H)$$.

#### Proof

Without loss of generality, we assume $\alpha <1$. Let

$$\begin{aligned} \psi ^n(t):=\int _0^t b(X^n_{\kappa _n(t)})\,dt. \end{aligned}$$

Let us apply the Girsanov theorem (Theorem 3.10) to the function $u(t)=b(X^n_{\kappa _n(t)})$. First let us check that all the conditions of this theorem hold.

First, we obviously have $|u(t)|\leqslant \Vert b\Vert _{\mathcal {C}^0}$, and thus (3.26) holds with $M= \Vert b\Vert _{\mathcal {C}^0}$.

Second, let us check condition (3.27) in the case $H>1/2$. Fix $\lambda >0$ and small $\delta >0$ such that $\alpha (H-\delta )>H-1/2$; such $\delta$ exists thanks to the assumption $\alpha >1-1/(2H)$. We apply Lemma 3.11 for the function $f:=b(X^n)$ and $\rho :=\alpha (H-\delta )$. We have

$$\begin{aligned}&\int _0^1\Bigl (\int _0^t\frac{(t/s)^{H-1/2}|b(X^n_{\kappa _n(t)})-b(X^n_{\kappa _n(s)})|}{(t-s)^{H+1/2}}\,ds\Bigr )^2\,dt\\&\quad \leqslant N[b(X^n)]_{\mathcal {C}^{\alpha (H-\delta )}}^2\\&\quad =N\Vert b\Vert _{\mathcal {C}^{\alpha }}^2[X^n]_{\mathcal {C}^{H-\delta }}^{2\alpha }\\&\quad \leqslant N \Vert b\Vert _{\mathcal {C}^{\alpha }}^2 (\Vert b\Vert _{\mathcal {C}^{0}}^{2\alpha }+ [B^H]_{\mathcal {C}^{H-\delta }}^{2\alpha })=:\xi \end{aligned}$$

Therefore,

4.8 $$\begin{aligned} \;\;{\mathbb {E}}\;e^{\lambda \xi }\leqslant N(\Vert b\Vert _{\mathcal {C}^{\alpha }},\alpha ,\delta ,H,\lambda )<\infty , \end{aligned}$$

where we used the fact that the Hölder constant $[B^H]_{\mathcal {C}^{H-\delta }}$ satisfies $\;\;{\mathbb {E}}\;\exp (\lambda [B^H]_{\mathcal {C}^{H-\delta }}^{2\alpha })\leqslant N$ for any $\lambda \geqslant 0$. Thus, condition (3.27) is satisfied. Hence all the conditions of Theorem 3.10 hold. Thus, there exists a probability measure ${\widetilde{\mathbb {P}}}$ equivalent to $\mathbb {P}$ such that the process ${\widetilde{B}}^H:=B^H+\psi ^n$ is a fractional *H*-Brownian motion on [0, 1] under ${\widetilde{\mathbb {P}}}$.

Now we can derive the desired bound (4.7). We have

4.9 $$\begin{aligned}&\;\;{\mathbb {E}}\;^{\mathbb {P}} \Bigl | \int _s^t \left( f(X^n_r)- f(X^n_{\kappa _n(r)}) \right) \, dr \Bigr |^p\nonumber \\&\qquad =\;\;{\mathbb {E}}\;^{{\widetilde{\mathbb {P}}}} \Bigl [\Bigl | \int _s^t \left( f(X^n_r)- f(X^n_{\kappa _n(r)}) \right) \, dr \Bigr |^p\frac{d\mathbb {P}}{d{\widetilde{\mathbb {P}}}}\Bigr ]\nonumber \\&\qquad \leqslant \Bigl (\;\;{\mathbb {E}}\;^{{\widetilde{\mathbb {P}}}} \Bigl | \int _s^t \left( f(X^n_r)- f(X^n_{\kappa _n(r)}) \right) \, dr \Bigr |^{2p}\Bigr )^{1/2}\Bigl (\;\;{\mathbb {E}}\;^{{\widetilde{\mathbb {P}}}}\Bigl [\frac{d\mathbb {P}}{d{\widetilde{\mathbb {P}}}}\Bigr ]^2\Bigr )^{1/2} \nonumber \\&\qquad =\Bigl (\;\;{\mathbb {E}}\;^{{\widetilde{\mathbb {P}}}} \Bigl | \int _s^t \left( f(\widetilde{B}^H_r+x_0^n)- f(\widetilde{B}^H_{\kappa _n(r)}+x_0^n) \right) \, dr \Bigr |^{2p}\Bigr )^{1/2}\Bigl (\;\;{\mathbb {E}}\;^{\mathbb {P}}\frac{d\mathbb {P}}{d{\widetilde{\mathbb {P}}}}\Bigr )^{1/2} \nonumber \\&\qquad =\Bigl (\;\;{\mathbb {E}}\;^{\mathbb {P}} \Bigl | \int _s^t \left( f( B^H_r+x_0^n)- f( B^H_{\kappa _n(r)}+x_0^n) \right) \, dr \Bigr |^{2p}\Bigr )^{1/2}\Bigl (\;\;{\mathbb {E}}\;^{\mathbb {P}}\frac{d\mathbb {P}}{d{\widetilde{\mathbb {P}}}}\Bigr )^{1/2}. \end{aligned}$$

Taking into account (4.8), we deduce by Theorem 3.10 that

$$\begin{aligned} \;\;{\mathbb {E}}\;^{\mathbb {P}}\frac{d\mathbb {P}}{d{\widetilde{\mathbb {P}}}}\leqslant N(\Vert b\Vert _{\mathcal {C}^{\alpha }},\alpha ,\delta ,H,\lambda ). \end{aligned}$$

Hence, using (4.1), we can continue (4.9) in the following way:

$$\begin{aligned} \;\;{\mathbb {E}}\;^{\mathbb {P}} \Bigl | \int _s^t \left( f(X^n_r)- f(X^n_{\kappa _n(r)}) \right) \, dr \Bigr |^p\leqslant N\Vert f\Vert _{\mathcal {C}^\alpha }^p n^{-p(\gamma (\alpha , H)+\varepsilon )}|t-s|^{p(1/2+\varepsilon )}, \end{aligned}$$

which implies the statement of the theorem. $\square$

### A regularization lemma

The goal of this subsection is to establish the regularization bound (4.26). Its proof consists of a number of steps. First, in Lemma 4.3 we derive an extension of the corresponding bound of Davie [9, Proposition 2.1] for the fractional Brownian motion case. It is important that the right–hand side of this bound depends on *p* as $\sqrt{p}$ (rather than *p*); this will be crucial later in the proof of Lemma 4.6 and Theorem 2.1. Then in Lemma 4.6 we obtain the pathwise version of this lemma and extend it to a wider class of processes (fractional Brownian motion with drift instead of a fractional Brownian motion). Finally, in Lemma 4.7 we obtain the desired regularization bound.

#### Lemma 4.3

Let $H\in (0,1)$, $\alpha \in (-1/(2H),0]$. Let $f\in \mathcal {C}^\infty$. Then there exists a constant $N=N(d,\alpha ,H)$ such that for any $p\geqslant 2$, $s,t\in [0,1]$ we have

4.10 $$\begin{aligned} \Bigl \Vert \int _s^t f(B_r^H)\,dr\Bigr \Vert _{L_p(\Omega )}\leqslant N\sqrt{p}\Vert f\Vert _{\mathcal {C}^\alpha } (t-s)^{H\alpha +1}. \end{aligned}$$

#### Remark 4.4

Note that the right–hand side of bound (4.10) depends only on the norm of *f* in $\mathcal {C}^\alpha$ and does not depend on the norm of *f* in other Hölder spaces.

#### Proof of Lemma 4.3

Fix $p\geqslant 2$. We will apply Theorem 3.3 to the process

$$\begin{aligned} \mathcal {A}_{t}:=\int _0^t f(B_r^H)\,dr, \quad t\in [0,1]. \end{aligned}$$

As usual, we write $\mathcal {A}_{s,t}:=\mathcal {A}_t-\mathcal {A}_s$. Let us check that all the conditions of that theorem hold with $m=4$

It is very easy to see that

$$\begin{aligned} \Vert \mathcal {A}_{s,t}\Vert _{L_{p\vee 4}(\Omega )}\leqslant \Vert f\Vert _{\mathcal {C}^0}|t-s|. \end{aligned}$$

Thus (3.13) holds. By Proposition 3.6 (iii) and Proposition 3.7 (i) we have for some $N_1=N_1(d,\alpha ,H)$ (recall that by assumptions $\alpha \leqslant 0$)

4.11 $$\begin{aligned} |\;\;{\mathbb {E}}\;^s \mathcal {A}_{s,t}|\leqslant \int _s^t |P_{c^2(s,r)}f(\;\;{\mathbb {E}}\;^s B_r^H)|dr\leqslant N_1\Vert f\Vert _{\mathcal {C}^\alpha }(t-s)^{H\alpha +1}. \end{aligned}$$

Hence

$$\begin{aligned} \Vert \;\;{\mathbb {E}}\;^s \mathcal {A}_{s,t}\Vert _{L_p(\Omega )}\leqslant N_1\Vert f\Vert _{\mathcal {C}^\alpha } (t-s)^{H\alpha +1} \end{aligned}$$

and condition (3.15) is met. We want to stress here that the constant $N_1$ here *does not* depend on *p* (this happens thanks to the a.s. bound (4.11); it will be crucial later in the proof)

Thus, it remains to check conditions (3.14) and (3.16). Fix $0\leqslant s\leqslant u\leqslant t\leqslant 1$. Using Proposition 3.6 (iii), we get

4.12 $$\begin{aligned} {\;\;{\mathbb {E}}\;^{s}}\mathcal {A}_{u,t}-{\;\;{\mathbb {E}}\;^{u}}\mathcal {A}_{u,t}&=\int _u^t \bigl (P_{c^2(s,r)}f(\;\;{\mathbb {E}}\;^s B_r^H)- P_{c^2(u,r)}f(\;\;{\mathbb {E}}\;^u B_r^H)\bigr )\,dr\nonumber \\&=\int _u^t \bigl (P_{c^2(s,r)}f(\;\;{\mathbb {E}}\;^s B_r^H)- P_{c^2(s,r)}f(\;\;{\mathbb {E}}\;^u B_r^H)\bigr )\,dr\nonumber \\&\quad +\int _u^t \bigl (P_{c^2(s,r)}f(\;\;{\mathbb {E}}\;^u B_r^H)- P_{c^2(u,r)}f(\;\;{\mathbb {E}}\;^u B_r^H)\bigr )\,dr\nonumber \\&=: I_1+I_2. \end{aligned}$$

Note that by Proposition 3.6 (ii), the random vector $\;\;{\mathbb {E}}\;^u B_r^H-\;\;{\mathbb {E}}\;^s B_r^H$ is independent of $\mathcal {F}^s$. Taking this into account and applying the conditional Minkowski inequality, we get

4.13 $$\begin{aligned} \Bigl (\;\;{\mathbb {E}}\;^s |I_1|^4\Bigr )^{\frac{1}{4}}&\leqslant \int _u^t \Bigl (\;\;{\mathbb {E}}\;^s\bigl [P_{c^2(s,r)}f(\;\;{\mathbb {E}}\;^s B_r^H)- P_{c^2(s,r)}f(\;\;{\mathbb {E}}\;^u B_r^H)\bigr ]^4\Bigr )^{\frac{1}{4}}\,dr\nonumber \\&\leqslant \int _u^t g_r(\;\;{\mathbb {E}}\;^s B_r^H)\,dr, \end{aligned}$$

where for $x\in \mathbb {R}^d$, $r\in [u,t]$ we denoted

$$\begin{aligned} g_r(x):=\Vert P_{c^2(s,r)}f(x)-P_{c^2(s,r)}f(x+\;\;{\mathbb {E}}\;^u B_r^H-\;\;{\mathbb {E}}\;^s B_r^H)\Vert _{L_4(\Omega )}. \end{aligned}$$

By Proposition 3.6 (ii), the random vector $\;\;{\mathbb {E}}\;^u B_r^H-\;\;{\mathbb {E}}\;^s B_r^H$ is Gaussian and consists of *d* independent components with each component of mean 0 and variance *v*(*s*, *u*, *t*) (recall its definition in (3.23)). Hence Proposition 3.7 (iv) yields now for some $N_2=N_2(d,\alpha ,H)$ and all $x\in \mathbb {R}^d$, $r\in [u,t]$

$$\begin{aligned} g_r(x)\leqslant N_2\Vert f\Vert _{\mathcal {C}^\alpha }(u-s)^{\frac{1}{2}}(r-u)^{H\alpha -\frac{1}{2}}. \end{aligned}$$

Substituting this into (4.13), we finally get

4.14 $$\begin{aligned} \Bigl (\;\;{\mathbb {E}}\;^s |I_1|^4\Bigr )^{\frac{1}{4}}\leqslant & {} N_2\Vert f\Vert _{\mathcal {C}^\alpha }(u-s)^{\frac{1}{2}}\int _u^t (r-u)^{H\alpha -\frac{1}{2}}\, dr\nonumber \\\leqslant & {} N_3\Vert f\Vert _{\mathcal {C}^\alpha }(u-s)^{\frac{1}{2}} (t-u)^{H\alpha +\frac{1}{2}}, \end{aligned}$$

for some $N_3=N_3(d,\alpha ,H)$ where we used that, by assumptions, $H\alpha -1/2>-1$.

Similarly, using Proposition 3.7 (iii) with $\beta =0$, we get for some $N_4=N_4(d,\alpha ,H)$

4.15 $$\begin{aligned} |I_2|\leqslant N\Vert f\Vert _{\mathcal {C}^\alpha }(u-s)^{\frac{1}{2}}\int _u^t (r-u)^{H\alpha -\frac{1}{2}}\,dr\leqslant N_4\Vert f\Vert _{\mathcal {C}^\alpha }(u-s)^{\frac{1}{2}} (t-u)^{H\alpha +\frac{1}{2}},\nonumber \\ \end{aligned}$$

where again we used that, by assumptions, $H\alpha -1/2>-1$. We stress that both $N_3$, $N_4$ *do not* depend on *p*.

Now to verify (3.14), we note that by (4.12), (4.14),(4.15), we have

4.16 $$\begin{aligned} \Vert \;\;{\mathbb {E}}\;^s\mathcal {A}_{u,t}-\;\;{\mathbb {E}}\;^u\mathcal {A}_{u,t}\Vert _{L_4(\Omega )}&\leqslant \Vert I_1\Vert _{L_4(\Omega )}+ \Vert I_2\Vert _{L_4(\Omega )}\nonumber \\&\leqslant \bigl (\;\;{\mathbb {E}}\;[\;\;{\mathbb {E}}\;^s|I_1|^4]\bigr )^{\frac{1}{4}}+\Vert I_2\Vert _{L_4(\Omega )}\nonumber \\&\leqslant (N_3+N_4)\Vert f\Vert _{\mathcal {C}^\alpha }(u-s)^{\frac{1}{2}}. \end{aligned}$$

Thus, condition (3.14) holds.

In a similar manner we check (3.16). We have

$$\begin{aligned} \;\;{\mathbb {E}}\;^s[|\;\;{\mathbb {E}}\;^s\mathcal {A}_{u,t}-\;\;{\mathbb {E}}\;^u\mathcal {A}_{u,t}|^2]&\leqslant 2 \;\;{\mathbb {E}}\;^s|I_1|^2 +2 \;\;{\mathbb {E}}\;^s|I_2|^2\leqslant 2\bigl (\;\;{\mathbb {E}}\;^s|I_1|^4\bigr )^{1/2} +2 \;\;{\mathbb {E}}\;^s|I_2|^2\\&\leqslant 2(N_3^2+N_4^2)\Vert f\Vert _{\mathcal {C}^\alpha }^2(u-s)(t-u)^{2H\alpha +1}. \end{aligned}$$

Thus,

$$\begin{aligned} \bigl \Vert \;\;{\mathbb {E}}\;^s[|\;\;{\mathbb {E}}\;^s\mathcal {A}_{u,t}-\;\;{\mathbb {E}}\;^u\mathcal {A}_{u,t}|^2]\bigr \Vert _{L_{p/2}(\Omega )}\leqslant 2(N_3^2+N_4^2)\Vert f\Vert _{\mathcal {C}^\alpha }^2(u-s)(t-u)^{2H\alpha +1} \end{aligned}$$

and the constant $2(N_3^2+N_4^2)$ does not depend on *p*. Therefore condition (3.16) holds.

Thus all the conditions of Theorem 3.3 hold. The statement of the theorem follows now from (3.17). $\square$

To establish the regularization bound we need the following simple corollary of the above lemma.

#### Corollary 4.5

Let $H\in (0,1)$, $\delta \in (0,1]$, $\alpha -\delta \in (-1/(2H),0]$. Let $f\in \mathcal {C}^\infty$. Then there exists a constant $N=N(d,\alpha ,H,\delta )$ such that for any $p\geqslant 2$, $s,t\in [0,1]$, $x,y\in \mathbb {R}^d$ we have

4.17 $$\begin{aligned} \Bigl \Vert \int _s^t (f(B_r^H+x)-f(B_r^H+y))\,dr\Bigr \Vert _{L_p(\Omega )}\leqslant N\sqrt{p}\Vert f\Vert _{\mathcal {C}^\alpha } (t-s)^{H(\alpha -\delta )+1}|x-y|^\delta .\nonumber \\ \end{aligned}$$

#### Proof

Fix $x,y\in \mathbb {R}^d$. Consider a function $g(z):=f(z+x)-f(z+y)$, $z\in \mathbb {R}^d$. Then, by Lemma 4.3

$$\begin{aligned} \Bigl \Vert \int _s^t (f(B_r^H+x)-f(B_r^H+y))\,dr\Bigr \Vert _{L_p(\Omega )}&= \Bigl \Vert \int _s^t g(B_r^H)\,dr\Bigr \Vert _{L_p(\Omega )}\\&\leqslant N\sqrt{p}\Vert g\Vert _{\mathcal {C}^{\alpha -\delta }} (t-s)^{H(\alpha -\delta )+1}. \end{aligned}$$

The corollary follows now immediately from Proposition 3.8. $\square$

The next lemma provides a pathwise version of bound (4.17). It also allows to replace fractional Brownian motion by fractional Brownian motion with a drift.

#### Lemma 4.6

Let $H\in (0,1)$, $\alpha >1-1/(2H)$, $\alpha \in [0,1]$, $f\in \mathcal {C}^\infty$. Let $\psi :\Omega \times [0,1]\rightarrow \mathbb {R}^d$ be an $\mathbb {F}$–adapted process such that $\psi _0$ is deterministic and for some $R>0$

4.18 $$\begin{aligned} \Vert \psi \Vert _{\mathcal {C}^1([0,1],\mathbb {R}^d)}\leqslant R,\quad a.s. \end{aligned}$$

Suppose that for some $\rho >H+1/2$ we have for any $\lambda >0$

4.19 $$\begin{aligned} \;\;{\mathbb {E}}\;\exp \big (\lambda \Vert \psi \Vert ^2_{\mathcal {C}^{\rho }([0,1],\mathbb {R}^d)}\big )=:G(\lambda )<\infty . \end{aligned}$$

Then for any $M>0$, $\varepsilon >0$, $\varepsilon _1>0$ there exists a constant $N=N(d,\alpha ,H,\varepsilon ,\varepsilon _1,G,R,M)$ and a random variable $\xi$ finite almost everywhere such that for any $s,t\in [0,1]$, $x,y\in \mathbb {R}$, $|x|, |y|\leqslant M$ we have

4.20 $$\begin{aligned}&\Bigl |\int _s^t (f(B_r^H+\psi _r+x)-f(B_r^H+\psi _r+y))dr\Bigr |\nonumber \\&\quad \leqslant \xi \Vert f\Vert _{\mathcal {C}^\alpha } (t-s)^{H(\alpha -1)+1-\varepsilon }|x-y| \end{aligned}$$

and

4.21 $$\begin{aligned} \;\;{\mathbb {E}}\;\exp (\xi ^{2-\varepsilon _1})<N<\infty . \end{aligned}$$

#### Proof

First we consider the case $\psi \equiv 0$. Fix $\varepsilon ,\varepsilon _1>0$. By the fundamental theorem of calculus we observe that for any $x,y\in \mathbb {R}^d$, $0\leqslant s \leqslant t \leqslant 1$

4.22 $$\begin{aligned}&\int _s^t (f(B_r^H+x)-f(B_r^H+y))\,dr\nonumber \\&\quad =(x-y)\cdot \int _0^1\int _s^t \nabla f(B_r^H+\theta x+(1-\theta )y)\,dr\,d\theta . \end{aligned}$$

Consider the process

$$\begin{aligned} F(t,z):=\int _0^t \nabla f(B_r^H+z)\,dr. \end{aligned}$$

Take $\delta >0$ such that $\alpha -1-\delta >1/(2H)$. By Lemma 4.3 and Corollary 4.5, there exists $N_1=N_1(\alpha ,d,H,\delta )$ such that for any $p\geqslant 2$, $s,t\in [0,1]$, $x,y\in \mathbb {R}^d$ we have

$$\begin{aligned} \Vert F(t,x)-F(s,y)\Vert _{L_p(\Omega )}&\leqslant \Vert F(t,x)-F(s,x)\Vert _{L_p(\Omega )}+\Vert F(s,x)-F(s,y)\Vert _{L_p(\Omega )}\\&\leqslant N_1\sqrt{p}\Vert \nabla f\Vert _{\mathcal {C}^{\alpha -1}}((t-s)^{H(\alpha -1)+1}+|x-y|^\delta ). \end{aligned}$$

We stress that $N_1$ does not depend on *p*. Taking into account that the process *F* is continuous (because $f\in \mathcal {C}^\infty )$, we derive from the above bound and the Kolmogorov continuity theorem ( [26, Theorem 1.4.1]) that for any *p* large enough one has

4.23 $$\begin{aligned} \sup _{\begin{array}{c} x,y\in \mathbb {R}^d, |x|,|y|\leqslant M\\ s,t\in [0,1] \end{array}} \frac{|F(t,x)-F(s,y)|}{(t-s)^{H(\alpha -1)+1-\varepsilon }+|x-y|^{\delta /2}}=: \xi \Vert f\Vert _{\mathcal {C}^{\alpha }}<\infty \,\,a.s., \end{aligned}$$

and $\Vert \xi \Vert _{L_p(\Omega )}\leqslant NN_1\sqrt{p}$, where $N=N(\alpha ,d,H,\delta ,\varepsilon ,M)$. Since *N* and $N_1$ do not depend on *p*, we see that by the Stirling formula

4.24 $$\begin{aligned} \;\;{\mathbb {E}}\;\exp (\xi ^{2-\varepsilon _1})=\sum _{n=0}^\infty \frac{\;\;{\mathbb {E}}\;\xi ^{n(2-\varepsilon _1)}}{n!}\leqslant \sum _{n=0}^\infty \frac{(NN_1)^{n(2-\varepsilon _1)}n^{n(1-\varepsilon _1/2)}}{n!}<\infty \end{aligned}$$

Therefore we obtain from (4.22) that for any $x,y\in \mathbb {R}^d$, $|x|,|y|\leqslant M$ we have

4.25 $$\begin{aligned}&\Bigl |\int _s^t (f(B_r^H+x)-f(B_r^H+y))\,dr\Bigr | \nonumber \\&\qquad \qquad \leqslant |x-y|\int _0^1 |(F(t,\theta x+(1-\theta )y)-F(s,\theta x+(1-\theta )y))|\,d\theta \nonumber \\&\qquad \qquad \leqslant \xi \Vert f\Vert _{\mathcal {C}^{\alpha }} (t-s)^{H(\alpha -1)+1-\varepsilon }|x-y|. \end{aligned}$$

Now we consider the general case. Assume that the function $\psi$ satisfies (4.19). Then by Proposition 3.10, bound (3.30) and assumption (4.19) the process

$$\begin{aligned} {\widetilde{B}}_t:=B_t+\psi _t-\psi _0 \end{aligned}$$

is a fractional Brownian motion with Hurst parameter *H* under some probability measure ${\widetilde{\mathbb {P}}}$ equivalent to $\mathbb {P}$. This yields from (4.25) (we apply this bound with $M+|\psi _0|$ in place of *M*)

$$\begin{aligned}&\Bigl |\int _s^t (f(B_r^H+\psi _r+x)-f(B_r^H+\psi _r+y))\,dr\Bigr |\\&\quad =\Bigl |\int _s^t (f({\widetilde{B}}_r^H+x+\psi _0)-f({\widetilde{B}}_r^H+y+\psi _0))\,dr\Bigr |\\&\quad \leqslant \eta \Vert f\Vert _{\mathcal {C}^{\alpha }} |x-y| \end{aligned}$$

where $\eta$ is a random variable with $\;\;{\mathbb {E}}\;^{{\widetilde{\mathbb {P}}}} \exp (\eta ^{2-\varepsilon _1})<\infty$. Note that we have used here our assumption that $\psi _0$ is non-random. The latter implies that for any $\varepsilon _2>\varepsilon _1$

$$\begin{aligned} \;\;{\mathbb {E}}\;^{\mathbb {P}} \exp (\eta ^{2-\varepsilon _2})&=\;\;{\mathbb {E}}\;^{{\widetilde{\mathbb {P}}}}\Bigl [ \exp (\eta ^{2-\varepsilon _2}) \frac{d \mathbb {P}}{d {\widetilde{\mathbb {P}}}}\Bigr ]\\&\leqslant \Bigl (\;\;{\mathbb {E}}\;^{{\widetilde{\mathbb {P}}}} \exp (2\eta ^{2-\varepsilon _2})\Bigr )^{1/2} \Bigl (\;\;{\mathbb {E}}\;^{ \mathbb {P}}\frac{d\mathbb {P}}{d{\widetilde{\mathbb {P}}}} \Bigr )^{1/2}\\&\leqslant \Bigl (\;\;{\mathbb {E}}\;^{{\widetilde{\mathbb {P}}}} \exp (2\eta ^{2-\varepsilon _2})\Bigr )^{1/2} e^{NR}\;\;{\mathbb {E}}\;^{ \mathbb {P}} \exp (N \Vert \psi \Vert ^2_{\mathcal {C}^{\rho }([0,1],\mathbb {R}^d)}) \end{aligned}$$

where the last inequality follows from (3.28) and (3.30). This concludes the proof of the theorem. $\square$

Now we are ready to present the main result of this subsection, the regularization lemma.

#### Lemma 4.7

Let $H\in (0,1)$, $\alpha >1-1/(2H)$, $\alpha \in [0,1]$, $p\geqslant 2$, $f\in \mathcal {C}^\alpha$, $\varepsilon ,\varepsilon _1>0$. Let $\tau \in (H(1-\alpha ),1)$. Let $\varphi , \psi :\Omega \times [0,1]\rightarrow \mathbb {R}^d$ be $\mathbb {F}$–adapted processes satisfying condition (4.18). Assume that $\psi$ satisfies additionally (4.19) for some $\rho >H+1/2$, $\rho \in [0,1]$. Suppose that $\psi _0$ and $\varphi _0$ are deterministic.

Then there exists a constant $N=N(H,\alpha ,p,d,\tau ,G,R,\varepsilon ,\varepsilon _1)$ such that for any $L>0$, and any $s,t\in [0,1]$ we have

4.26 $$\begin{aligned}&\Bigl \Vert \int _s^t (f(B_r^H+\varphi _r)-f(B_r^H+\psi _r))\,dr\Bigr \Vert _{L_p(\Omega )}\nonumber \\&\quad \leqslant NL \Vert f\Vert _{\mathcal {C}^\alpha } (t-s)^{H(\alpha -1)+1-\varepsilon }\big (\Vert \varphi _s-\psi _s\Vert _{L_p(\Omega )}+ \Vert [\varphi -\psi ]_{C^{\tau }([s,t])}\Vert _{L_p(\Omega )}(t-s)^\tau \big )\nonumber \\&\qquad + N \Vert f\Vert _{\mathcal {C}^0}|t-s|\exp (-L^{2-\varepsilon _1}). \end{aligned}$$

#### Proof

We begin with assuming further that $f\in \mathcal {C}^\infty (\mathbb {R}^d,\mathbb {R}^d)$. Fix $S,T\in [0,1]_{\leqslant }$, $\varepsilon _1>0$. Choose any $\varepsilon >0$ small enough such that

4.27 $$\begin{aligned} H(\alpha -1)-\varepsilon +\tau >0. \end{aligned}$$

Let us apply the deterministic sewing lemma (Proposition 3.1) to the process

$$\begin{aligned} A_{s,t}:=\int _s^t (f(B_r^H+\psi _r+\varphi _s-\psi _s)-f(B_r^H+\psi _r))\,dr,\quad (s,t)\in [S,T]_{\leqslant }. \end{aligned}$$

Let us check that all the conditions of the above lemma are satisfied.

First, the process *A* is clearly continuous, since *f* is bounded. Then, using Lemma 4.6 with $M:=4R$, we derive that for any $S\leqslant s\leqslant u\leqslant T$ there exists a random variable $\xi$ with $\;\;{\mathbb {E}}\;\exp (\xi ^{2-\varepsilon _1})\leqslant N=N(d,\alpha ,H,\varepsilon ,\varepsilon _1,G,|\varphi _0|,|\psi _0|,R)<\infty$ such that

$$\begin{aligned} |\delta A_{s,u,t}|&=\Bigl |\int _u^t (f(B_r^H+\psi _r+\varphi _u-\psi _u)-f(B_r^H+\psi _r+\varphi _s-\psi _s))\,dr\Bigr |\\&\leqslant \xi \Vert f\Vert _{\mathcal {C}^\alpha }|(\psi _u-\varphi _u)-(\psi _s-\varphi _s)|(t-s)^{H(\alpha -1)+1-\varepsilon }\\&\leqslant \xi \Vert f\Vert _{\mathcal {C}^\alpha }[\psi -\varphi ]_{\mathcal {C}^\tau ([S,T])}(t-s)^{H(\alpha -1)+1-\varepsilon +\tau }. \end{aligned}$$

Since, by (4.27), $H(\alpha -1)+1-\varepsilon +\tau >1$, we see that condition (3.6) is satisfied with $C=\xi \Vert f\Vert _{\mathcal {C}^\alpha }[\psi -\varphi ]_{\mathcal {C}^\tau ([S,T])}$. Thus, all the conditions of Proposition 3.1 hold. By setting now

$$\begin{aligned} {\tilde{\mathcal {A}}}_t:=\int _s^t (f(B_r^H+\varphi _r)-f(B_r^H+\psi _r))\,dr, \end{aligned}$$

we see that for $S\leqslant s\leqslant t\leqslant T$

$$\begin{aligned} |{\tilde{\mathcal {A}}}_t-{\tilde{\mathcal {A}}}_s-A_{s,t}|&=\Bigl |\int _s^t (f(B_r^H+\varphi _r)-f(B_r^H+\psi _r+\varphi _s-\psi _s))\,dr\Bigr |\Bigr |\\&\leqslant \Vert f\Vert _{\mathcal {C}^1}[\psi -\varphi ]_{\mathcal {C}^\tau ([S,T])}|t-s|^{1+\tau } \\&\leqslant \Vert f\Vert _{\mathcal {C}^1}[\psi -\varphi ]_{\mathcal {C}^\tau ([S,T])}|t-s|^{H(\alpha -1)+1-\varepsilon +\tau }. \end{aligned}$$

Thus, the process ${\tilde{\mathcal {A}}}$ satisfies (3.7) and therefore coincides with $\mathcal {A}$. Proposition 3.1 implies now that for any $S\leqslant s\leqslant t\leqslant T$

$$\begin{aligned}&\Bigl |\int _s^t (f(B_r^H+\varphi _r)-f(B_r^H+\psi _r))\,dr\Bigr |\\&\quad \leqslant |A_{s,t}|+N \xi \Vert f\Vert _{\mathcal {C}^\alpha }[\psi -\varphi ]_{\mathcal {C}^\tau ([S,T])}(t-s)^{H(\alpha -1)+1-\varepsilon +\tau }\\&\quad \leqslant N\xi \Vert f\Vert _{\mathcal {C}^\alpha }(t-s)^{H(\alpha -1)+1-\varepsilon }\bigl (|\psi -\varphi |_{\mathcal {C}^0([S,T])}+ [\psi -\varphi ]_{\mathcal {C}^\tau ([S,T])}(t-s)^{\tau }\bigr ), \end{aligned}$$

where the bound on $|A_{s,t}|$ follows again from Lemma 4.6. By putting in the above bound $s=S$ and $t=T$ and using that $|\psi -\varphi |_{\mathcal {C}^0([S,T])}\leqslant |\psi _S-\varphi _S|+ [\psi -\varphi ]_{\mathcal {C}^\tau ([S,T])}(T-S)^{\tau }$, we obtain for $S,T\in [0,1]_{\leqslant }$

$$\begin{aligned}&\Bigl |\int _S^T (f(B_r^H+\varphi _r)-f(B_r^H+\psi _r))\,dr\Bigr |\\&\quad \leqslant N\xi \Vert f\Vert _{\mathcal {C}^\alpha }(T-S)^{H(\alpha -1)+1-\varepsilon }\bigl (|\psi _S-\varphi _S|+ [\psi -\varphi ]_{\mathcal {C}^\tau ([S,T])}(T-S)^{\tau }\bigr ). \end{aligned}$$

On the other hand, we have the following trivial bound.

$$\begin{aligned} \Bigl |\int _S^T (f(B_r^H+\varphi _r)-f(B_r^H+\psi _r))\,dr\Bigr |\leqslant 2\Vert f\Vert _{\mathcal {C}^0}|T-S|. \end{aligned}$$

Therefore,

$$\begin{aligned}&\Bigl \Vert \int _S^T (f(B_r^H+\varphi _r)-f(B_r^H+\psi _r))\,dr\Bigr \Vert _{L_p(\Omega )} \\&\quad \leqslant \Bigl \Vert \mathbf {1}_{\xi \leqslant L}\int _S^T (f(B_r^H+\varphi _r)-f(B_r^H+\psi _r))\,dr\Bigr \Vert _{L_p(\Omega )} \\&\qquad +\Bigl \Vert \mathbf {1}_{\xi \geqslant L}\int _S^T (f(B_r^H+\varphi _r)-f(B_r^H+\psi _r))\,dr\Bigr \Vert _{L_p(\Omega )} \\&\quad \leqslant LN \Vert f\Vert _{\mathcal {C}^\alpha }(T-S)^{H(\alpha -1)+1-\varepsilon }\bigl (\Vert \psi _S-\varphi _S\Vert _{L_p(\Omega )}+ \Vert [\psi -\varphi ]_{\mathcal {C}^\tau ([S,T])}\Vert _{L_p(\Omega )}(T-S)^{\tau }\bigr ) \\&\qquad +2\big (\mathbb {P}(\xi \geqslant L)\big )^{1/p}\Vert f\Vert _{\mathcal {C}^0}|T-S|. \end{aligned}$$

By Chebyshev inequality and (4.21), we finally get (4.26) for the case of smooth *f*.

Now we are ready to remove the extra assumption on the smoothness of *f*. Let us set $f_n= \mathcal {P}_{1/n}f \in \mathcal {C}^\infty$. By applying the statement of the lemma to $f_n$ and using that $\Vert f_n\Vert _{\mathcal {C}^ \beta } \leqslant \Vert f\Vert _{\mathcal {C}^\beta }$ for $\beta =\alpha , 0$ we get

4.28 $$\begin{aligned}&\Bigl \Vert \int _s^t ( f_n(B_r^H+\varphi _r)- f_n(B_r^H+\psi _r))\,dr\Bigr \Vert _{L_p(\Omega )}\nonumber \\&\quad \leqslant NL \Vert f\Vert _{\mathcal {C}^\alpha } (t-s)^{H(\alpha -1)+1-\varepsilon }(\Vert \varphi _s-\psi _s\Vert _{L_p(\Omega )}+ \Vert [\varphi -\psi ]_{C^{\tau }([s,t])}\Vert _{L_p(\Omega )}(t-s)^\tau )\nonumber \\&\qquad + N \Vert f\Vert _{\mathcal {C}^0}|t-s|\exp (-L^{2-\varepsilon _1}). \end{aligned}$$

If $\alpha >0$, then $f_n(x) \rightarrow f(x)$ for all $x \in \mathbb {R}^d$ and the claim follows by Fatou’s lemma. So we only have to consider the case $\alpha =0$. Clearly, it suffices to show that for each $r>0$, almost surely

$$\begin{aligned} ( f_n(B_r^H+\varphi _r)- f_n(B_r^H+\psi _r)) \rightarrow ( f(B_r^H+\varphi _r)- f(B_r^H+\psi _r)), \end{aligned}$$

as $n \rightarrow \infty$. Notice that almost surely $f_n(B^H_r) \rightarrow f(B^H_r)$ as $n \rightarrow \infty$, since the law of $B^H_r$ is absolutely continuous (for $r>0$). Moreover, since $\alpha =0$, we have by assumption that $H< 1/2$. By Proposition 3.10 (recall that $\varphi$ satisfies (4.18), therefore is Lipschitz) there exists a neasure equivalent to $\mathbb {P}$ under which $B^H+ \varphi$ is a fractional brownian motion. Consequently, for all $r >0$, almost surely

$$\begin{aligned} f_n(B_r^H+\varphi _r) \rightarrow f(B_r^H+\varphi _r), \end{aligned}$$

as $n \rightarrow \infty$. With the same reasoning we obtain that almost surely $f_n(B_r^H+\psi _r) \rightarrow f(B_r^H+\psi _r)$. The lemma is now proved. $\square$

### Proof of Theorem 2.1

#### Proof

Without loss of generality we assume $\alpha \ne 1$. Let us denote

$$\begin{aligned} \psi _t:=x_0+\int _0^t b(X_r)\,dr,\quad \psi ^n_t:=x^n_0+\int _0^t b(X^n_{\kappa _n(r)})\,dr,\quad t\in [0,1]. \end{aligned}$$

Fix $\varepsilon >0$ such that

4.29 $$\begin{aligned} \varepsilon <\frac{1}{2}+H(\alpha -1). \end{aligned}$$

By assumption (2.5) such $\varepsilon$ exists. Fix now large enough $p\geqslant 2$ such that

4.30 $$\begin{aligned} d/p<\varepsilon /2. \end{aligned}$$

Fix $0\leqslant S\leqslant T\leqslant 1$. Then, taking into account (4.7), for any $S\leqslant s\leqslant t\leqslant T$ we have

4.31 $$\begin{aligned}&\Vert (\psi _t- \psi _s)-(\psi ^n_t- \psi ^n_s)\Vert _{L_p(\Omega )}\nonumber \\&\quad =\Bigl \Vert \int _s^t (b(X_r)-b(X^n_{\kappa _n(r)}))\,dr\Bigr \Vert _{L_p(\Omega )}\nonumber \\&\quad \leqslant \Bigl \Vert \int _s^t (b(X_r)-b(X^n_r))\,dr\Bigr \Vert _{L_p(\Omega )}+N|t-s|^{1/2+\varepsilon } n^{-\gamma +\varepsilon }. \end{aligned}$$

Let $M\geqslant 1$ be a parameter to be fixed later. We wish to apply Lemma 4.7 with $\psi ^n$ in place of $\varphi$, $\frac{1}{2}+H(\alpha -1)-\varepsilon$ in place of $\varepsilon$, and $\tau :=1/2+\varepsilon /2$. Let us check that all the conditions of this lemma are satisfied. First, we note that by (4.29) we have $\frac{1}{2}+H(\alpha -1)-\varepsilon >0$, which is required by the assumptions of the lemma. Second, we note that $1/2+\varepsilon /2>H(1-\alpha )$ thanks to (2.5), thus this choice of $\tau$ is allowed. Next, it is clear that $\psi _0$ and $\psi ^n_0$ are deterministic. Further, since the function *b* is bounded, we see $\psi$ and $\psi ^n$ satisfy (4.18). Finally, let us verify that $\psi$ satisfies (4.19). If $H<1/2$, this condition holds automatically thanks to the boundedness of *b*. If $H\geqslant 1/2$ then pick $H'\in (0,H)$ such that

4.32 $$\begin{aligned} \alpha H'>H-\frac{1}{2}. \end{aligned}$$

Note that such $H'$ exists thanks to assumption (2.5). Then, by definition of $\psi$, we clearly have

$$\begin{aligned}{}[\psi ]_{\mathcal {C}^{1+\alpha H'}}\leqslant |x_0|+\Vert b\Vert _{\mathcal {C}^0}+[b(X_{\cdot })]_{\mathcal {C}^{\alpha H'}} \leqslant |x_0|+\Vert b\Vert _{\mathcal {C}^0}+\Vert b\Vert _{\mathcal {C}^0}^{\alpha }+[B^H]_{{\mathcal {C}^{ H'}}}^\alpha . \end{aligned}$$

Therefore for any $\lambda >0$ we have

$$\begin{aligned} \;\;{\mathbb {E}}\;e^{\lambda [\psi ]_{\mathcal {C}^{1+\alpha H'}}^2}\leqslant N(|x_0|,\Vert b\Vert _{\mathcal {C}^0})\;\;{\mathbb {E}}\;\exp ([B^H]_{{\mathcal {C}^{ H'}}}^{2\alpha })<\infty . \end{aligned}$$

By taking $\rho := 1+\alpha H'$ and recalling (4.32), we see that $\rho >H+1/2$ and thus condition (4.19) holds. Therefore all conditions of Lemma 4.7 are met. Applying this lemma, we get

$$\begin{aligned}&\Bigl \Vert \int _s^t (b(X_r)- b(X^n_r))\,dr\Bigr \Vert _{L_p(\Omega )}\\&\quad =\Bigl \Vert \int _s^t (b(B^H_r+\psi _r)-b(B^H_r+\psi ^n_r))\,dr\Bigr \Vert _{L_p(\Omega )} \\&\quad \leqslant M N|t-s|^{\frac{1}{2}+\varepsilon }\Vert \psi _S-\psi _S^n\Vert _{L_p(\Omega )} \\&\qquad + MN|t-s|^{1+3\varepsilon /2} \Vert [\psi -\psi ^n]_{\mathcal {C}^{1/2+\varepsilon /2}([s,t])}\Vert _{L_p(\Omega )}+ N \exp (-M^{2-\varepsilon _0})|t-s|\\&\quad \leqslant M N|t-s|^{\frac{1}{2}+\varepsilon }\Vert \psi _S-\psi _S^n\Vert _{L_p(\Omega )} \\&\qquad + MN|t-s|^{1+3\varepsilon /2} [] \psi -\psi ^n []_{\mathscr {C}^{1/2+\varepsilon }_p,[s,t]}+ N \exp (-M^{2-\varepsilon _0})|t-s|, \end{aligned}$$

where the last inequality follows from the Kolmogorov continuity theorem and (4.30). Using this in (4.31), dividing by $|t-s|^{1/2+\varepsilon }$ and taking supremum over $S\leqslant s\leqslant t\leqslant T$, we get for some $N_1\geqslant 1$ independent of *M*, *n*

4.33 $$\begin{aligned}&[] \psi -\psi ^n []_{\mathscr {C}^{1/2+\varepsilon }_p,[S,T]}\nonumber \\&\quad \leqslant MN_1 \Vert \psi _S-\psi ^n_S\Vert _{L_p(\Omega )} +MN_1|T-S|^{1/2+\varepsilon /2}[] \psi -\psi ^n []_{\mathscr {C}^{1/2+\varepsilon }_p,[S,T]}\nonumber \\&\quad + N_1 n^{-\gamma +\varepsilon }+N_1 \exp (-M^{2-\varepsilon _0}). \end{aligned}$$

Fix now *m* to be the smallest integer so that $N_1M m^{-1/2-\varepsilon /2}\leqslant 1/2$ (we stress that *m* does not depend on *n*). One gets from (4.33)

4.34 $$\begin{aligned}&[] \psi -\psi ^n []_{\mathscr {C}^{1/2+\varepsilon }_p,[S,S+1/m]} \leqslant 2M N_1 \Vert \psi _S-\psi ^n_S\Vert _{L_p(\Omega )} \nonumber \\&\quad + 2N_1 n^{-\gamma +\varepsilon }+2N_1 \exp (-M^{2-\varepsilon _0}), \end{aligned}$$

and thus

$$\begin{aligned}&\Vert \psi _{S+1/m}-\psi ^n_{S+1/m}\Vert _{L_p(\Omega )} \leqslant 2MN_1 \Vert \psi _S-\psi ^n_S\Vert _{L_p(\Omega )} \\&\quad + 2N_1 n^{-\gamma +\varepsilon }+2N_1 \exp (-M^{2-\varepsilon _0}). \end{aligned}$$

Starting from $S=0$ and applying the above bound *k* times, $k=1,\ldots ,m$, one can conclude

$$\begin{aligned} \Vert \psi _{k/m}-\psi ^n_{k/m}\Vert _{L_p(\Omega )}&\leqslant (2MN_1)^k \Bigl (\Vert \psi _0-\psi ^n_0\Vert _{L_p(\Omega )}\\&\quad + 2N_1 n^{-\gamma +\varepsilon }+ +2N_1 \exp (-M^{2-\varepsilon _0})\Bigr )\\&\leqslant (2MN_1)^m \Bigl (|x_0-x^n_0| \\&\quad + 2N_1 n^{-\gamma +\varepsilon } +2N_1 \exp (-M^{2-\varepsilon _0})\Bigr ). \end{aligned}$$

Substituting back into (4.34), we get

4.35 $$\begin{aligned}{}[] \psi -\psi ^{n} []_{\mathscr {C}^{1/2+\varepsilon }_p([0,1])}&\leqslant m \sup _{k=1,\dots ,m}[] \psi -\psi ^{n} []_{\mathscr {C}^{1/2+\varepsilon }_p([k/m,(k+1)/m])}\nonumber \\&\leqslant (2N_1M)^{m+5}\Bigl (|x_0-x_0^n|+N_1 n^{-\gamma +\varepsilon }+N_1 \exp (-M^{2-\varepsilon _0})\Bigr ). \end{aligned}$$

It follows from the definition of *m* that $m\leqslant 2N_1^2M^{2-\varepsilon }$. At this point we choose $\varepsilon _0=\varepsilon /2$ and note that for some universal constant $N_2$ one has

$$\begin{aligned} (2N_1M)^{m+5}=e^{(m+5)\log (2 N_1M)}\leqslant e^{(2N_1^2M^{2-\varepsilon }+5)\log (2 N_1M)}\leqslant N_2 e^{\frac{1}{2}M^{2-\varepsilon /2}}. \end{aligned}$$

Thus, we can continue (4.35) as follows.

4.36 $$\begin{aligned}&[] \psi -\psi ^{n} []_{\mathscr {C}^{1/2+\varepsilon }_p([0,1])} \leqslant e^{N_3M^{2-\varepsilon }\log M}\nonumber \\&\quad \Bigl (|x_0-x_0^n|+N_1 n^{-\gamma +\varepsilon }\Bigr )+N_1N_2 \exp (-\frac{1}{2}M^{2-\varepsilon /2}). \end{aligned}$$

Fix now $\delta >0$ and choose $N_4=N_4(\delta )$ such that for all $M>0$ one has

$$\begin{aligned} \exp (\frac{1}{2}M^{2-\varepsilon /2})\geqslant N_4 e^{\delta ^{-1}N_3M^{2-\varepsilon }\log M}. \end{aligned}$$

It remains to notice that by choosing $M>1$ such that

$$\begin{aligned} e^{N_3M^{2-\varepsilon }\log M}= n^{\delta }, \end{aligned}$$

one has

$$\begin{aligned} e^{-\frac{1}{2}M^{2-\varepsilon /2}}\leqslant N n^{-1}. \end{aligned}$$

Substituting back to (4.36) and since $X-X^n=\psi -\psi ^n$, we arrive to the required bound (2.6). $\square$

## Malliavin calculus for the Euler–Maruyama scheme

In the multiplicative standard Brownian case, we first consider Euler–Maruyama schemes without drift: for any $y\in \mathbb {R}^d$ define the process ${\bar{X}}^n(y)$ by

5.1 $$\begin{aligned} d{\bar{X}}^n_t(y)=\sigma ({\bar{X}}^n_{\kappa _n(t)}(y))\,dB_t,\quad {\bar{X}}^n_0=y. \end{aligned}$$

This process will play a similar role as $B^H$ in the previous section. Similarly to the proof of Lemma 4.1, we need sharp bounds on the conditional distribution of ${\bar{X}}^n_t$ given $\mathcal {F}_s$, which can be obtained from bounds of the density of ${\bar{X}}^n_t$. A trivial induction argument yields that for $t>0$, ${\bar{X}}^n_t$ indeed admits a density, but to our knowledge such inductive argument can not be used to obtain useful quantitative information.

### Remark 5.1

While the densities of Euler–Maruyama approximations have been studied in the literature, see e.g. [5, 6, 18], none of the available estimates suited well for our purposes. In [18], under less regularity assumption on $\sigma$, $L_p$ bounds of the density (but not its derivatives) are derived. In [5, 6], smoothness of the density is obtained even in a hypoelliptic setting, but without sharp control on the short time behaviour of the norms.

### Theorem 5.2

Let $\sigma$ satisfy (2.8), ${\bar{X}}^n$ be the solution of (5.1), and let $G\in \mathcal {C}^1$. Then for all $t=1/n,2/n,\ldots ,1$ and $k=1,\ldots ,d$ one has the bound

5.2 $$\begin{aligned} |\;\;{\mathbb {E}}\;\partial _k G({\bar{X}}^n_t)|\leqslant N \Vert G\Vert _{\mathcal {C}^0}t^{-1/2} + N\Vert G\Vert _{\mathcal {C}^1}e^{-cn} \end{aligned}$$

with some constant $N=N(d,\lambda ,\Vert \sigma \Vert _{\mathcal {C}^2})$ and $c=c(d,\Vert \sigma \Vert _{\mathcal {C}^2})>0$.

We will prove Theorem 5.2 via Malliavin calculus. In our discrete situation, of course this could be translated to finite dimensional standard calculus, but we find it more instructive to follow the basic terminology of [35], which we base on the lecture notes [21].

### Definitions

Define $H=\{h=(h_i)_{i=1,\ldots ,n}:\,h_i\in \mathbb {R}^d\}$, with the norm

$$\begin{aligned} \Vert h\Vert ^2_H=\frac{1}{n}\sum _{i=1}^n|h_i|^2=\frac{1}{n}\sum _{i=1}^n\sum _{k=1}^d|h_i^k|^2. \end{aligned}$$

One can obtain a scalar product from $\Vert \cdot \Vert _H$, which we denote by $\langle \cdot ,\cdot \rangle _H$. Let us also denote $\mathcal {I}=\{1,\ldots ,n\}\times \{1,\ldots ,d\}$. One can of course view *H* as a copy of $\mathbb {R}^\mathcal {I}$, with a rescaled version of the usual $\ell _2$ norm. We denote by $e_{(i,k)}$ the element of *H* whose elements are zero apart from the *i*-th one, which is the *k*-th unit vector of $\mathbb {R}^d$. Set $\Delta W_{(i,k)}:=W^{k}_{i/n}-W^k_{(i-1)/n}$. Then for any $\mathbb {R}$-valued random variable *X* of the form

$$\begin{aligned} X=F(\Delta W_{(i,k)}:\,(i,k)\in \mathcal {I}), \end{aligned}$$

where *F* is a differentiable function, with at most polynomially growing derivative, the Malliavin derivative of *X* is defined as the *H*-valued random variable

$$\begin{aligned} \mathscr {D}X := \sum _{(i,k)\in \mathcal {I}}(\mathscr {D}^k_i X)e_{(i,k)} :=\sum _{(i,k)\in \mathcal {I}}\partial _{(i,k)}F( \Delta W_{(j,\ell )}:\,(j,\ell )\in \mathcal {I})e_{(i,k)}. \end{aligned}$$

For multidimensional random variables we define $\mathscr {D}$ coordinatewise. In the sequel we also use the matrix norm on $\mathbb {R}^{d\times d}$ defined in the usual way $\Vert M\Vert :=\sup _{x\in \mathbb {R}^d, |x|=1}|Mx|$. Recall that if *M* is positive semidefinite, then one has $\Vert M\Vert =\sup _{x\in \mathbb {R}^d, |x|=1}x^*Mx$. It follows that $\Vert \cdot \Vert$ is monotone increasing with respect to the usual order $\preceq$ on the positive semidefinite matrices.

The following few properties are true in far larger generality, for the proofs we refer to [21]. One easily sees that the derivative $\mathscr {D}$ satisfies the chain rule: namely, for any differentiable $G:\mathbb {R}^d\rightarrow \mathbb {R}$, one has $\mathscr {D}G(X)=\nabla G(X)\cdot \mathscr {D}X$. The operator $\mathscr {D}$ is closable, and its closure will also be denoted by $\mathscr {D}$, whose domain we denote by $\mathcal {W}\subset L_2(\Omega )$. The adjoint of $\mathscr {D}$ is denoted by $\delta$. One then has that the domain of $\delta$ is included in $\mathcal {W}(H)$ and the following identity holds:

5.3 $$\begin{aligned} \;\;{\mathbb {E}}\;|\delta u|^2=\;\;{\mathbb {E}}\;\Vert u\Vert ^2_H+\;\;{\mathbb {E}}\;\frac{1}{n^2}\sum _{(i,k),(j,m)\in \mathcal {I}}(\mathscr {D}^k_i u^m_j)(\mathscr {D}^m_j u^k_i). \end{aligned}$$

### Stochastic difference equations

First let us remark that the Eq. (5.1) does not define an invertible stochastic flow: indeed, for any $t>0$, $y\rightarrow {\bar{X}}^n_t(y)$ may not even be one-to-one. Therefore in order to invoke arguments from the Malliavin calculus for diffusion processes, we consider a modified process equation that does define an invertible flow. Unfortunately, this new process will not have a density, but its singular part (as well as its difference from the original process) is exponentially small.

Take a smooth function $\varrho :\mathbb {R}\rightarrow \mathbb {R}$ such that $|\varrho (r)|\leqslant |r|$ for all $r\in \mathbb {R}$, $\varrho (r)=r$ for $|r|\leqslant (4\Vert \sigma \Vert _{\mathcal {C}^1} d^2)^{-1}$, $\varrho (r)=0$ for $|r|\geqslant (2\Vert \sigma \Vert _{\mathcal {C}^1} d^2)^{-1}$, and that satisfies $|\partial ^k\varrho |\leqslant N$ for $k=0,\ldots ,3$ with some $N=N(d,\Vert \sigma \Vert _{\mathcal {C}^1})$. Define the recursion, for $x\in \mathbb {R}^d$ and $j=1,\ldots , n$, $k=1,\ldots ,d$

5.4 $$\begin{aligned} \mathcal {X}_{j}^{k}(x)=\mathcal {X}_{j-1}^k(x)+\sum _{\ell =1}^d\sigma ^{k\ell }\big (\mathcal {X}_{j-1}(x)\big )\varrho (\Delta W_{(j,\ell )}),\qquad \mathcal {X}_{0}(x)=x. \end{aligned}$$

By our definition of $\varrho$, for any *j*, (5.4) defines a diffeomorphism from $\mathbb {R}^d$ to $\mathbb {R}^d$ by $x\rightarrow \mathcal {X}_{j}(x)$. It is easy to see that its Jacobian $J_{j}(x)=\big (J_{j}^{m,k}(x)\big )=\big (\partial _{x^m}\mathcal {X}^k_{j}(x)\big )_{k,m=1,\ldots ,d; \,j=1,\ldots ,n}$ satisfies the recursion

$$\begin{aligned} J_{j}^{m,k}(x)=J_{j-1}^{m,k}(x)+ \sum _{q=1}^d J_{j-1}^{m,q}(x) \Big [\sum _{\ell =1}^d\partial _{q}\sigma ^{k\ell }\big (\mathcal {X}_{j-1}(x)\big )\varrho (\Delta W_{(j,\ell )})\Big ],\qquad J_{0}(x)=\mathrm {id}. \end{aligned}$$

It is also clear that $\mathscr {D}_i^m\mathcal {X}^k_j=0$ for $j<i$, while for $j>i$ we have the recursion

$$\begin{aligned} \mathscr {D}_i^m\mathcal {X}^k_j(x)= & {} \mathscr {D}_i^m\mathcal {X}^k_{j-1}(x) + \sum _{q=1}^d\mathscr {D}_i^m \mathcal {X}^q_{j-1}(x) \Big [ \sum _{\ell =1}^d \partial _q\sigma ^{k\ell }\big (\mathcal {X}_{j-1}(x)\big ) \varrho (\Delta W_{(j,\ell )})\Big ], \\ \mathscr {D}^m_i\mathcal {X}^k_i= & {} \sigma ^{km}\big (\mathcal {X}_{i-1}(x)\big )\varrho '(\Delta W_{(i,m)}). \end{aligned}$$

From now on we will usually suppress the dependence on *x* in the notation. Save for the initial conditions, the two recursions coincide for the matrix-valued processes $J_\cdot$ and $\mathscr {D}_i \mathcal {X}_\cdot$. Since the recursion is furthermore linear, $j\mapsto J_j^{-1}\mathscr {D}_i \mathcal {X}_j$ is constant in time for $j\geqslant i\geqslant 1$. In particular,

$$\begin{aligned} J_{j}^{-1}\mathscr {D}_i \mathcal {X}_j=J_i^{-1}\big (\sigma ^{km}(\mathcal {X}_{i-1})\varrho '(\Delta W_{(i,m)})\big )_{k,m=1,\ldots ,d}\,, \end{aligned}$$

or, with the notation $J_{i,j}=J_jJ_i^{-1}$,

$$\begin{aligned} \mathscr {D}_i \mathcal {X}_j=J_{i,j}\big (\sigma ^{km}(\mathcal {X}_{i-1})\varrho '(\Delta W_{(i,m)})\big )_{k,m=1,\ldots ,d}\,. \end{aligned}$$

Let us now define the event ${\hat{\Omega }}\subset \Omega$ by

$$\begin{aligned} {\hat{\Omega }}=\{|\Delta W_{(i,k)}|\leqslant (4\Vert \sigma \Vert _{\mathcal {C}^1} d^2)^{-1}, \forall (i,k)\in \mathcal {I}\} \end{aligned}$$

as well as the (matrix-valued) random variables $\mathcal {D}_{i,j}$ by

5.5 $$\begin{aligned} \mathcal {D}_{i,j}=J_{i,j}\sigma (\mathcal {X}_{i-1}). \end{aligned}$$

Clearly, on ${\hat{\Omega }}$ one has $\mathcal {D}_{i,j}=\mathscr {D}_i \mathcal {X}_j$. Note that for fixed *j*, *m* one may view $\mathcal {D}_{\cdot ,j}^{\cdot ,m}$ as an element of *H*, while for fixed *i*, *j* one may view $\mathcal {D}_{i,j}$ as a $d\times d$ matrix. One furthermore has the following exponential bound on ${\hat{\Omega }}$.

#### Proposition 5.3

There exist *N* and $c>0$ depending only on *d* and $\Vert \sigma \Vert _{\mathcal {C}^1}$, one has $\mathbb {P}({\hat{\Omega }})\geqslant 1-Ne^{-cn}$.

#### Proof

For each $(i,k)\in \mathcal {I}$, since $\Delta W_{(i,k)}$ is zero mean Gaussian with variance $n^{-1}$, one has

$$\begin{aligned} \mathbb {P}\big (\varrho (\Delta W_{(i,k)})\ne \Delta W_{(i,k)}\big )\leqslant \mathbb {P}\big (|\Delta W_{(i,k)}|\geqslant (4\Vert \sigma \Vert _{\mathcal {C}^1} d^2)^{-1}\big )\leqslant N'e^{-c'n} \end{aligned}$$

with some $N'$ and $c'>0$ depending only on *d* and $\Vert \sigma \Vert _{\mathcal {C}^1}$, by the standard properties of the Gaussian distribution. Therefore, by the elementary inequality $(1-x)^\alpha \geqslant 1-\alpha x$, valid for all $x\in [0,1]$ and $\alpha \geqslant 1$, one has

$$\begin{aligned} \mathbb {P}({\hat{\Omega }})\geqslant \big (1-(N'e^{-c'n}\wedge 1)\big )^{nd}\geqslant 1-N'nde^{-c'n}\geqslant 1-Ne^{-(c'/2)n}. \end{aligned}$$

$$\square $$

We now fix $(j,k)\in \mathcal {I}$, $G\in \mathcal {C}^1$, and we aim to bound $|\;\;{\mathbb {E}}\;\partial _k G(X_j)|$ in terms of $t:=j/n$ and $\Vert G\Vert _0$, and some additional exponentially small error term. To this end, we define the Malliavin matrix $\mathscr {M}\in \mathbb {R}^{d\times d}$

$$\begin{aligned} \mathscr {M}^{m,q}=\langle \mathcal {D}_{\cdot ,j}^{\cdot ,m},\mathcal {D}_{\cdot ,j}^{\cdot ,q}\rangle _H=\frac{1}{n}\sum _{(i,v)\in \mathcal {I}}\mathcal {D}_{i,j}^{v,m}\mathcal {D}_{i,j}^{v,q}, \end{aligned}$$

with $m,q=1,\ldots ,d$. As we will momentarily see (see (5.21)), $\mathscr {M}$ is invertible. Define

$$\begin{aligned} Y=\sum _{m=1}^d(\mathcal {D}_{\cdot ,j}^{\cdot ,m})(\mathscr {M}^{-1})^{m,k}\in H. \end{aligned}$$

One then has by the chain rule that on ${\hat{\Omega }}$, $\partial _k G(\mathcal {X}_j)= \langle \mathscr {D}G(X_j),Y\rangle _H$. Therefore,

5.6 $$\begin{aligned} \;\;{\mathbb {E}}\;\partial _k G(\mathcal {X}_j)= & {} \;\;{\mathbb {E}}\;\langle \mathscr {D}G(X_j),Y\rangle _H+\;\;{\mathbb {E}}\;\partial _k G(\mathcal {X}_j)\mathbf {1}_{{\hat{\Omega ^c}}}-\;\;{\mathbb {E}}\;\langle \mathscr {D}G(\mathcal {X}_j),Y\rangle _H\mathbf {1}_{{\hat{\Omega ^c}}} \nonumber \\= & {} \;\;{\mathbb {E}}\;(G( X_j),\delta Y)+\;\;{\mathbb {E}}\;\partial _k G(\mathcal {X}_j)\mathbf {1}_{{\hat{\Omega ^c}}}-\;\;{\mathbb {E}}\;\langle \mathscr {D}G(\mathcal {X}_j),Y\rangle _H\mathbf {1}_{{\hat{\Omega ^c}}} \nonumber \\=: & {} \;\;{\mathbb {E}}\;(G( \mathcal {X}_j),\delta Y)+I_1+I_2. \end{aligned}$$

Recalling (5.3), one has

5.7 $$\begin{aligned} \;\;{\mathbb {E}}\;|\delta Y|^2\leqslant \;\;{\mathbb {E}}\;\Vert Y\Vert ^2_H+\;\;{\mathbb {E}}\;\frac{1}{n^2}\sum _{(i,q),(r,m)\in \mathcal {I}}(\mathscr {D}^q_i Y^m_r)(\mathscr {D}^m_rY^q_i). \end{aligned}$$

Theorem 5.2 will then follow easily once we have the appropriate moment bounds of the objects above. Recall the notation $t=j/n$.

#### Lemma 5.4

Assume the above notations and let $\sigma$ satisfy (2.8). Then for any $p>0$, one has the bounds

5.8 $$\begin{aligned} \;\;{\mathbb {E}}\;\sup _{i=1,\ldots ,j}\Vert J_{i,j}(x)\Vert ^p+\;\;{\mathbb {E}}\;\sup _{1\leqslant i\leqslant j}\Vert J_{i,j}^{-1}(x)\Vert ^p\leqslant & {} N, \end{aligned}$$

5.9 $$\begin{aligned} \;\;{\mathbb {E}}\;\sup _{i=1,\ldots ,j}\Vert \mathcal {D}_{i,j}(x)\Vert ^p\leqslant & {} N, \end{aligned}$$

5.10 $$\begin{aligned} \;\;{\mathbb {E}}\;\Vert \mathscr {M}^{-1}(x)\Vert ^p\leqslant & {} Nt^{-p}, \end{aligned}$$

5.11 $$\begin{aligned} \sup _{i =1,\ldots , j}\;\;{\mathbb {E}}\;\sup _{r=1,\ldots ,j}\Vert \mathscr {D}_i Y_r(x)\Vert ^p\leqslant & {} N t^{-p}. \end{aligned}$$

for all $x \in \mathbb {R}^d$, with some $N=N(p,d,\lambda ,\Vert \sigma \Vert _{\mathcal {C}^2})$.

#### Proof

As before, we omit the dependence on $x\in \mathbb {R}^d$ in order to ease the notation. We first bound the moments of $\sup _j\Vert J_j\Vert$. Recall that we have the recursion

5.12 $$\begin{aligned} J_j= J_{j-1}(I+ \Gamma _{j/n}), \end{aligned}$$

where the matrix $\Gamma _t=(\Gamma _t)_{q,k=1}^d$ is given by

5.13 $$\begin{aligned} \Gamma ^{q,k}_t = \sum _{\ell =1}^d \partial _q \sigma ^{k \ell } ( \mathcal {X}_{n \kappa _n(t)}) \varrho ( W^\ell _t -W^\ell _{\kappa _n(t)}), \end{aligned}$$

By Itô’s formula it follows that

$$\begin{aligned} \varrho ( W^\ell _t -W^\ell _{\kappa _n(t)})= \int _{{\kappa _n(t)}}^t \varrho '(W^\ell _s-W^\ell _{\kappa _n(t)}) \, dW^\ell _s + \frac{1}{2}\int _{{\kappa _n(t)}}^t\varrho ''(W^\ell _s-W^\ell _{\kappa _n(t)}) \, ds. \end{aligned}$$

Consequently, for $j=0, \ldots , n$ we have that $J_j= Z_{j/n}$, where the matrix-valued process $Z_t$ satisfies

5.14 $$\begin{aligned} dZ_t = \sum _{q=1}^d Z_{\kappa _n(t)}\mathcal {A}_t \, dt + \sum _{\ell =1}^d Z_{\kappa _n(t)} \mathcal {B}^{\ell }_t dW^\ell _t, \qquad Z_0= I, \end{aligned}$$

with matrices $\mathcal {A}_s=(\mathcal {A}^{q, k}_s)_{q,k=1,\ldots ,d}$ and $\mathcal {B}^\ell _s=(\mathcal {B}^{\ell ,q,k}_s)_{q,k=1,\ldots ,d}$ given by

$$\begin{aligned} \mathcal {A}^{q,k}_s= & {} \frac{1}{2} \sum _{\ell =1}^d \partial _q \sigma ^{ k \ell } (\mathcal {X}_{n \kappa _n(s)})\varrho ''(W^\ell _s-W^\ell _{\kappa _n(s)}) \\ \mathcal {B}^{\ell ,q,k}_s= & {} \partial _q \sigma ^{k \ell } (\mathcal {X}_{n \kappa _n})\varrho '(W^\ell _s-W^\ell _{\kappa _n(s)}). \end{aligned}$$

Notice that there exists a constant $N=N (\Vert \sigma \Vert _{\mathcal {C}^1}, \Vert \varrho \Vert _{\mathcal {C}^2})$ such that almost surely, for all $(t, x) \in [0,1] \times \mathbb {R}^d$

5.15 $$\begin{aligned} \Vert \mathcal {A}_t\Vert + \sum _{\ell =1}^d\Vert \mathcal {B}^{\ell }_t\Vert \leqslant N. \end{aligned}$$

This bound combined with the fact that $Z_t$ satisfies (5.14) imply the bounds

$$\begin{aligned} \;\;{\mathbb {E}}\;\sup _{t \leqslant 1} \Vert Z_t\Vert ^p \leqslant N \end{aligned}$$

for all $p>0$. Hence,

5.16 $$\begin{aligned} \;\;{\mathbb {E}}\;\sup _{j=1,..,n}\Vert J_j\Vert ^p \leqslant \;\;{\mathbb {E}}\;\sup _{t \leqslant 1} \Vert Z_t\Vert ^p \leqslant N. \end{aligned}$$

We now bound the moments of $\sup _j \Vert J^{-1}_j\Vert$. By (5.12) we get

5.17 $$\begin{aligned} J_j^{-1}=(I+ \Gamma _{j/n})^{-1} J_{j-1}^{-1} \end{aligned}$$

Recall that for $t \in [ (j-1)/n, j/n]$

$$\begin{aligned} \Gamma _t= \int _{(j-1)/n}^t \mathcal {A}_s \, ds + \sum _{\ell =1}^d \int _{(j-1)/n}^t \mathcal {B}^\ell _s \, dW^\ell _s, \end{aligned}$$

and that by the definition of $\varrho$ and (5.13), for all $t \in [0,T]$, the matrix $I+\Gamma _t$ is invertible. Hence, by Itô’s formula, we have for $t \in [ (j-1)/n, j/n]$

5.18 $$\begin{aligned} (I+\Gamma _t)^{-1}= I +\int _{(j-1)/n}^t {\tilde{{\mathcal {A}}}}_s \, ds + \sum _{\ell =1}^d \int _{(j-1)/n}^t {\tilde{{\mathcal {B}}}}^\ell _s \, d W^\ell _s, \end{aligned}$$

with

$$\begin{aligned} {\tilde{{\mathcal {A}}}}_s= & {} \sum _{\ell =1}^d (I+\Gamma _s)^{-1} \mathcal {B}_s^\ell (I+\Gamma _s)^{-1}\mathcal {B}_s^\ell (I+\Gamma _s)^{-1} -(I+\Gamma _s)^{-1} \mathcal {A}_s(I+\Gamma _s)^{-1}, \\ {\tilde{{\mathcal {B}}}}_s^\ell= & {} -(I+\Gamma _s)^{-1} \mathcal {B}^\ell _s(I+\Gamma _s)^{-1}. \end{aligned}$$

Moreover, by definition or $\varrho$, almost surely, for all $(t,x) \in [0,T] \times \mathbb {R}^d$ one has

5.19 $$\begin{aligned} \Vert {\tilde{{\mathcal {A} }}}_t\Vert +\sum _{\ell =1}^d \Vert {\tilde{{\mathcal {B}}}}^\ell _t \Vert \leqslant N. \end{aligned}$$

By (5.17) and (5.18), for $j=1,...,n$ we have that $J^{-1}_j= {\tilde{Z}}_{j/n}$, where the matrix valued process ${\tilde{Z}}_t$ is defined by

$$\begin{aligned} d{\tilde{Z}}_t= \tilde{\mathcal {A}}_t {\tilde{Z}}_{\kappa _n(t)} \, dt + \sum _{\ell =1}^d \tilde{\mathcal {B}}^\ell _t {\tilde{Z}}_{\kappa _n(t)} \, dW^\ell _s, \qquad {\tilde{Z}}_0 =I . \end{aligned}$$

By this and the bounds (5.19) we have the bounds

$$\begin{aligned} \;\;{\mathbb {E}}\;\sup _{t \leqslant 1} \Vert {\tilde{Z}}_t\Vert ^p \leqslant N \end{aligned}$$

for all $p>0$. Consequently,

5.20 $$\begin{aligned} \;\;{\mathbb {E}}\;\sup _{j=1,...,n} \Vert J^{-1}_j\Vert ^p \leqslant \;\;{\mathbb {E}}\;\sup _{t \leqslant 1} \Vert {\tilde{Z}}_t\Vert ^p \leqslant N. \end{aligned}$$

Finally, from (5.16) and (5.20) we obtain (5.8).

The bound (5.9) then immediately follows from (5.8), the definition (5.5), and the boundedness of $\sigma$.

Next, we show (5.10). On the set of positive definite matrices we have that on one hand, matrix inversion is a convex mapping, and on the other hand, the function $\Vert \cdot \Vert ^p$ is a convex increasing mapping for $p\geqslant 1$. It is also an elementary fact that if $B\succeq \lambda I$, then $\Vert (ABA^*)^{-1}\Vert \leqslant \lambda ^{-1}\Vert (AA^*)^{-1}\Vert$. One then writes

5.21 $$\begin{aligned} \Vert \mathscr {M}^{-1}\Vert ^p= & {} \Big (\frac{n}{j}\Big )^p\Big \Vert \Big (\frac{1}{j}\sum _{i=1}^j\big [J_{i,j}\sigma (\mathcal {X}_{i-1})\big ]\big [J_{i,j}\sigma (\mathcal {X}_{i-1})\big ]^*\Big )^{-1}\Big \Vert ^p \nonumber \\\leqslant & {} t^{-p}\frac{1}{j}\sum _{i=1}^j\Vert \big (\big [J_{i,j}\sigma (\mathcal {X}_{i-1})\big ]\big [J_{i,j}\sigma (\mathcal {X}_{i-1})\big ]^*\big )^{-1}\Vert ^p \nonumber \\\leqslant & {} \lambda ^{-p}t^{-p}\frac{1}{j}\sum _{i=1}^j\Vert J_{i,j}^{-1}\Vert ^{2p} \nonumber \\\leqslant & {} \lambda ^{-p}t^{-p}\sup _{i=1,\ldots ,j}\Vert J_{i,j}^{-1}\Vert ^{2p}. \end{aligned}$$

Therefore (5.10) follows from (5.8)

We now move to the proof of (5.11). First of all, notice that the above argument yields

5.22 $$\begin{aligned} \sup _{i = 1,...,n} \;\;{\mathbb {E}}\;\sup _{j=1,...,n} \Vert \mathscr {D}_i \mathcal {X}_j\Vert ^p \leqslant N. \end{aligned}$$

for all $p>0$. Indeed, the proof of this is identical to the proof of (5.16) since $(\mathscr {D}_i \mathcal {X}_j)_{j \geqslant i}$ has the same dynamics as $(J_j)_{j\geqslant 0}$ and initial condition $\mathscr {D}^k_i \mathcal {X}^m_i=\sigma ^{km} ( \mathcal {X}_{i-1}) \varrho '(\Delta W_{(i,m)})$ which is bounded. Recall that

$$\begin{aligned} Y_r = \sum _{m=1}^d ( \mathcal {D}^{\cdot , m}_{r,j}) (\mathscr {M}^{-1})^{m,k}. \end{aligned}$$

By Leibniz’s rule, for each $i, r \in \{0,..,n\}$, $\mathscr {D}_iY^r$ is a $\mathbb {R}^d \otimes \mathbb {R}^d$-valued random variable given by

5.23 $$\begin{aligned} \mathscr {D}_iY_r= \sum _{m=1}^d ( \mathscr {D}_i \mathcal {D}^{\cdot , m}_{r,j}) (\mathscr {M}^{-1})^{m,k}+ \sum _{m=1}^d \mathcal {D}^{\cdot , m}_{r,j} \otimes \mathscr {D}_i (\mathscr {M}^{-1})^{m,k} \end{aligned}$$

We start with a bound for $\sup _r \Vert \mathscr {D}_i \mathcal {D}_{r,j}\Vert$. By definition of $\mathcal {D}_{i,j}$ we have that

5.24 $$\begin{aligned} \mathscr {D}_i\mathcal {D}_{r,j} = (\mathscr {D}_iJ_j ) J^{-1}_r \sigma (\mathcal {X}_{r-1})+ J_j (\mathscr {D}_iJ^{-1}_r) \sigma (\mathcal {X}_{r-1})+ J_j J^{-1}_r (\mathscr {D}_i \sigma (\mathcal {X}_{r-1})),\nonumber \\ \end{aligned}$$

where for $A \in (\mathbb {R}^d)^{\otimes 2}$, $B \in (\mathbb {R}^d)^{\otimes 3}$, the product *AB* or *BA* is an element of $(\mathbb {R}^d)^{\otimes 3}$ that arises by considering *B* as a $d\times d$ matrix whose entries are elements of $\mathbb {R}^d$. We estimate the term $\mathscr {D} _i J_j$. As before, we have that $\mathscr {D}_i J_j = \mathscr {D}_i Z_{j/n}$, where *Z* is given by (5.14). We have that $\mathscr {D}_i Z_t=0$ for $t <i/n$ while for $t \geqslant i/n$ the process $\mathscr {D}_i Z_t=:\mathscr {Z}^i_t$ satisfies

5.25 $$\begin{aligned} \mathscr {Z}^i_t= & {} \left( \mathscr {Z}^i_{\kappa _n(t)} \mathcal {A}_t + Z_{\kappa _n(t)} \mathscr {D}_iA_t \right) \, dt+ \sum _{\ell =1}^d \left( \mathscr {Z}^i_{\kappa _n(t)} \mathcal {B}^\ell _t + Z_{\kappa _n(t)} \mathscr {D}_i \mathcal {B}^\ell _t \right) dW^\ell _t \nonumber \\ \mathscr {Z}^i_{i/n}= & {} Z_{i/n}\sum _{\ell =1}^d \mathcal {B}^\ell _{i/n} \end{aligned}$$

By the chain rule and (5.22) it follows that for $p>0$ there exists $N=N(\Vert \sigma \Vert _{\mathcal {C}^2}, \Vert \varrho \Vert _{\mathcal {C}^3}, d,p)$ such that

5.26 $$\begin{aligned} \sup _{i=1,...,n} \;\;{\mathbb {E}}\;\left( \sup _{t \leqslant 1} \Vert \mathscr {D}_i \mathcal {A}_t\Vert ^p + \sum _{\ell =1}^d\sup _{t \leqslant 1}\Vert \mathscr {D}_i \mathcal {B}^\ell _t \Vert ^p \right) \leqslant N \end{aligned}$$

This combined with (5.16) shows that for the ‘free terms’ of (5.25) we have

$$\begin{aligned} \sup _{i=1,...,n} \;\;{\mathbb {E}}\;\left( \sup _{t \leqslant 1} \Vert Z_{\kappa _n(t)}\mathscr {D}_i \mathcal {A}_t\Vert ^p + \sum _{\ell =1}^d\sup _{t \leqslant 1}\Vert Z_{\kappa _n(t)} \mathscr {D}_i \mathcal {B}^\ell _t \Vert ^p \right) \leqslant N. \end{aligned}$$

This, along with (5.15) and (5.16), implies that

5.27 $$\begin{aligned} \sup _{i=1,...,n} \;\;{\mathbb {E}}\;\sup _{j=1,...,n} \Vert \mathscr {D}_i J_j\Vert ^p \leqslant \sup _{i=1,...,n} \;\;{\mathbb {E}}\;\sup _{i/n \leqslant t \leqslant 1} \Vert \mathscr {Z}^i_t \Vert ^p \leqslant N. \end{aligned}$$

This in turn, combined with (5.20) and the boundedness of $\sigma$, implies that

$$\begin{aligned} \sup _{i=1,...,n} \;\;{\mathbb {E}}\;\sup _{r=1,...,n} \Vert (\mathscr {D}_iJ_j ) J^{-1}_r \sigma (\mathcal {X}_{r-1})\Vert ^p \leqslant N. \end{aligned}$$

Next, by the chain rule we have

$$\begin{aligned} \Vert J_j (\mathscr {D}_iJ^{-1}_r) \sigma (\mathcal {X}_{r-1})\Vert \leqslant \Vert J_j \Vert \Vert J_r^{-1}\Vert ^{2}\Vert \mathscr {D}_iJ_r\Vert \Vert \sigma (\mathcal {X}_{r-1})\Vert . \end{aligned}$$

By (5.16), (5.20), (5.27), and the boundedness of $\sigma$, we see that

$$\begin{aligned} \sup _{i=1,...,n} \;\;{\mathbb {E}}\;\sup _{r=1,...,n}\Vert J_j (\mathscr {D}_iJ^{-1}_r) \sigma (\mathcal {X}_{r-1})\Vert ^p \leqslant N. \end{aligned}$$

Finally, from (5.16), (5.20), the boundedness of $\nabla \sigma$, and (5.22) we get

$$\begin{aligned} \sup _{i=1,...,n} \;\;{\mathbb {E}}\;\sup _{r=1,...,n}\Vert J_j J^{-1}_r (\mathscr {D}_i \sigma (\mathcal {X}_{r-1})\Vert ^p \leqslant N. \end{aligned}$$

Recalling (5.24), we obtain

5.28 $$\begin{aligned} \sup _{i=1,...,n} \;\;{\mathbb {E}}\;\sup _{r=1,...,n}\Vert \mathscr {D}_i\mathcal {D}_{r,j}\Vert ^p \leqslant N, \end{aligned}$$

which combined with (5.10) gives

5.29 $$\begin{aligned} \sup _{i=1,...,n} \;\;{\mathbb {E}}\;\sup _{r=1,...,n}\Vert \sum _{m=1}^d ( \mathscr {D}_i \mathcal {D}^{\cdot , m}_{r,j}) (\mathscr {M}^{-1})^{m,k} \Vert ^p \leqslant N t^{-p}. \end{aligned}$$

We proceed by obtaining a similar bound for the second term at the right hand side of (5.23). First, let us derive a bound for $\mathscr {D}_i \mathscr {M}$. For each entry $\mathscr {M}^{m,q}$ of the matrix $\mathscr {M}$ we have

$$\begin{aligned} \mathscr {D}_i \mathscr {M}^{m,q} = \frac{1}{n} \sum _{\ell =1}^n \sum _{v=1}^d \left( \mathcal {D}_{\ell ,j}^{v,q}\mathscr {D}_i \mathcal {D}_{\ell ,j}^{v,m} + \mathcal {D}_{\ell ,j}^{v,m} \mathscr {D}_i\mathcal {D}_{\ell ,j}^{v,q}\right) . \end{aligned}$$

Then, notice that on $\hat{\Omega }$, for $\ell >j$ we have $\mathcal {D}_{\ell ,j}= \mathscr {D}_\ell \mathcal {X}_j=0$. Hence, by taking into account (5.9) and (5.28) we get

$$\begin{aligned} \sup _{i=1,...,n} \big (\;\;{\mathbb {E}}\;\Vert \mathscr {D}_i \mathscr {M}^{m,q}\Vert ^p \big ) ^{1/p} \leqslant N\big ( \frac{j}{n}+ n (\mathbb {P}(\hat{\Omega }^c))^{1/p}\big )\leqslant N \big ( \frac{j}{n}+ n e^{-cn/p}\big ) \leqslant N \frac{j}{n}=Nt . \end{aligned}$$

Summation over *m*, *q* gives

5.30 $$\begin{aligned} \sup _{i=1,...,n} \big (\;\;{\mathbb {E}}\;\Vert \mathscr {D}_i \mathscr {M}\Vert ^p \big ) ^{1/p} \leqslant N t . \end{aligned}$$

Therefore, we get

$$\begin{aligned} \Vert \sum _{m=1}^d \mathcal {D}^{\cdot , m}_{r,j} \otimes \mathscr {D}_i (\mathscr {M}^{-1})^{m,k}\Vert \leqslant N \Vert \mathcal {D}_{r,j}\Vert \Vert \mathscr {M}^{-1}\Vert ^2 \Vert \mathscr {D}_i\mathscr {M}\Vert , \end{aligned}$$

which by virtue of (5.9), (5.10), and (5.30) gives

$$\begin{aligned} \;\;{\mathbb {E}}\;\Vert \sum _{m=1}^d \mathcal {D}^{\cdot , m}_{r,j} \otimes \mathscr {D}_i (\mathscr {M}^{-1})^{m,k}\Vert ^p \leqslant N t^{-p}. \end{aligned}$$

This combined with (5.29), by virtue of (5.23), proves (5.11). This finishes the proof. $\square$

### Proof of Theorem 5.2

#### Proof

Recalling that $Y_i=0$ for $i>j$, we can write, using (5.9) and (5.10),

$$\begin{aligned} \;\;{\mathbb {E}}\;\Vert Y\Vert _H^2\leqslant \;\;{\mathbb {E}}\;\frac{1}{n}\sum _{i=1}^j(\sup _{i=1,\ldots ,j}\Vert \mathcal {D}_{i,j}\Vert \Vert \mathscr {M}^{-1}\Vert )^2\leqslant N(j/n)t^{-2}\leqslant Nt^{-1}. \end{aligned}$$

One also has

$$\begin{aligned} |\;\;{\mathbb {E}}\;\frac{1}{n^2}\sum _{(i,q),(r,m)\in \mathcal {I}}(\mathscr {D}^q_i Y^m_r)(\mathscr {D}^m_rY^q_i)| \leqslant t^2 \;\;{\mathbb {E}}\;\sup _{i,r=1,\ldots j}\Vert \mathscr {D}_i Y_r\Vert ^2\leqslant N. \end{aligned}$$

Therefore, by (5.7), we have the following bound on the main (first) term on the right-hand side of (5.6)

$$\begin{aligned} |\;\;{\mathbb {E}}\;(G(\mathcal {X}_j),\delta Y)|\leqslant \Vert G\Vert _{\mathcal {C}^0}(\;\;{\mathbb {E}}\;|\delta Y|^2)^{1/2}\leqslant N t^{-1/2}\Vert G\Vert _{\mathcal {C}^0}. \end{aligned}$$

As for the other two terms, Proposition 5.3 immediately yields

$$\begin{aligned} |I_1|\leqslant N\Vert G\Vert _{\mathcal {C}^1}e^{-cn}, \end{aligned}$$

while for $I_2$ we can write

$$\begin{aligned} |I_2|\leqslant & {} Ne^{-cn}\Big [\;\;{\mathbb {E}}\;\Big (\frac{1}{n}\sum _{i=1}^j(\mathscr {D}_iG(\mathcal {X}_j),Y_i)\Big )^2 \Big ]^{1/2} \\\leqslant & {} N e^{-cn} t\frac{1}{j}\sum _{i=1}^j \big (\;\;{\mathbb {E}}\;\sup _{i=1,\ldots ,j}|\mathscr {D}_i G(\mathcal {X}_j)|^6\big )^{1/6} \big (\;\;{\mathbb {E}}\;\sup _{i=1,\ldots ,j}\Vert \mathcal {D}_{i,j}\Vert ^6\big )^{1/6} \big (\;\;{\mathbb {E}}\;\Vert \mathscr {M}^{-1}\Vert ^6\big )^{1/6} \\\leqslant & {} N\Vert G\Vert _{\mathcal {C}^1}e^{-cn}. \end{aligned}$$

Therefore, by (5.6), we obtain

$$\begin{aligned} |\;\;{\mathbb {E}}\;\partial _k G(\mathcal {X}_j)\Vert \leqslant N \Vert G\Vert _{\mathcal {C}^0}t^{-1/2} + N\Vert G\Vert _{\mathcal {C}^1}e^{-cn}, \end{aligned}$$

and since on ${\hat{\Omega }}$, one has $\mathcal {X}_j={\bar{X}}^n_{j/n}={\bar{X}}^n_t$, the bound (5.2) follows. $\square$

## Multiplicative Brownian noise

### Quadrature estimates

#### Lemma 6.1

Let $y\in \mathbb {R}^d$, $\varepsilon _1\in (0,1/2)$, $\alpha \in (0,1)$, $p>0$. Suppose that $\sigma$ satisfies (2.8) and that ${\bar{X}}^n:={\bar{X}}^n(y)$ is the solution of (5.1). Then for all $f\in \mathcal {C}^\alpha$, $0\leqslant s\leqslant t\leqslant 1$, $n\in \mathbb {N}$, one has the bound

6.1 $$\begin{aligned} \big \Vert \int _s^t (f({\bar{X}}_r^n)-f({\bar{X}}_{\kappa _n(r)}^n))\, dr\big \Vert _{L_p(\Omega )} \leqslant N\Vert f\Vert _{\mathcal {C}^\alpha } n^{-1/2+2 \varepsilon _1}|t-s|^{1/2+\varepsilon _1} , \end{aligned}$$

with some $$N=N(\alpha , p, d,\varepsilon _1,\lambda ,\Vert \sigma \Vert _{\mathcal {C}^2})$$.

#### Proof

It clearly suffices to prove the bound for $p\geqslant 2$, and, as in [10], for $f\in \mathcal {C}^\infty$. We put for $0\leqslant s\leqslant t\leqslant T$

$$\begin{aligned} A_{s,t}:=\;\;{\mathbb {E}}\;^s \int _s^t (f({\bar{X}}_r^n)-f({\bar{X}}_{\kappa _n(r)}^n))\, dr. \end{aligned}$$

Then, clearly, for any $0\leqslant s\leqslant u\leqslant t\leqslant T$

$$\begin{aligned} \delta A_{s,u,t}:&=A_{s,t}-A_{s,u}-A_{u,t}\\&=\;\;{\mathbb {E}}\;^s \int _u^t (f({\bar{X}}_r^n)-f({\bar{X}}_{\kappa _n(r)}^n))\, dr-\;\;{\mathbb {E}}\;^u \int _u^t(f({\bar{X}}_r^n)-f({\bar{X}}_{\kappa _n(r)}^n))\, dr. \end{aligned}$$

Let us check that all the conditions (3.8)-(3.9) of the stochastic sewing lemma are satisfied. Note that

$$\begin{aligned} \;\;{\mathbb {E}}\;^s \delta A_{s,u,t}=0, \end{aligned}$$

and so condition (3.9) trivially holds, with $C_2=0$. As for (3.8), let $s \in [k/n, (k+1)/n)$ for some $k \in \mathbb {N}_0$. Suppose first that $t \in [(k+4)/n, 1]$. We write

$$\begin{aligned} |A_{s,t}|= | I_1+I_2|:= \Big |\Big (\int _s^{(k+4)/n} +\int _{(k+4)/n}^t\Big ) \;\;{\mathbb {E}}\;^s \big ( f({\bar{X}}^n_r)-f({\bar{X}}^n_{k_n(r)})\big )\, dr\Big |. \end{aligned}$$

For $I_2$ we write,

$$\begin{aligned} I_2 = \;\;{\mathbb {E}}\;^s \int _{(k+4)/n}^t \;\;{\mathbb {E}}\;^{(k+1)/n}\big (\;\;{\mathbb {E}}\;^{\kappa _n(r)} f({\bar{X}}^n_r)-f({\bar{X}}^n_{k_n(r)})\big ) \, dr. \end{aligned}$$

Next, denote by $p_{\Sigma }$ the density of a Gaussian vector in $\mathbb {R}^d$ with covariance matrix $\Sigma$ and let $\mathcal {P}_{\Sigma } f =p_{\Sigma }* f$ (recall that for $\theta \geqslant 0$, we denote $p_\theta := p _{\theta I}$, where *I* is the $d \times d$ identity matrix). With this notation, we have

$$\begin{aligned} \;\;{\mathbb {E}}\;^{k_n(r)} f\left( {\bar{X}}^n_{k_n(r)}+\sigma ({\bar{X}}^n_{k_n(r)}) (W_r-W_{k_n(r)})\right) =\mathcal {P}_{\sigma \sigma ^{\intercal }({\bar{X}}^n_{k_n(r)})(r-k_n(r))} f ({\bar{X}}^n_{k_n(r)}), \end{aligned}$$

so with

$$\begin{aligned} g(x):=g^n_r(x):=f(x)-\mathcal {P}_{\sigma \sigma ^{\intercal } (x)(r-\kappa _n(r))}f(x) \end{aligned}$$

we have

6.2 $$\begin{aligned} I_2=\;\;{\mathbb {E}}\;^s\int _{(k+4)/n}^t\;\;{\mathbb {E}}\;^{(k+1)/n}g^n_r({\bar{X}}^n_{\kappa _n(r)})\,dr. \end{aligned}$$

Moreover, notice that by (2.8) we have for a constant $N=(\Vert \sigma \Vert _{\mathcal {C}^1}, \alpha )$

6.3 $$\begin{aligned} \Vert g\Vert _{\mathcal {C}^{\alpha /2}} \leqslant N \Vert f\Vert _{\mathcal {C}^\alpha }. \end{aligned}$$

Let us use the shorthand $\delta =r-\kappa _n(r)\leqslant n^{-1}$. We can then write

6.4 $$\begin{aligned} \mathcal {P}_\varepsilon g (x) =&\int _{\mathbb {R}^d}\int _{\mathbb {R}^d} p_\varepsilon (z) p_{\sigma \sigma ^{\intercal } (x-z)\delta }( y) \big (f(x-z)- f(x-y-z) \big ) \, dy \,dz \nonumber \\ =&\int _{\mathbb {R}^d}\int _{\mathbb {R}^d} p_\varepsilon (z) p_{\sigma \sigma ^{\intercal } (x-z)\delta }( y) \int _0^1 y_i \partial _{z_i}f(x-z-\theta y) \,d\theta dy \,dz \nonumber \\ =&\int _{\mathbb {R}^d}\int _{\mathbb {R}^d} \partial _{z_i}\big ( p_\varepsilon (z) p_{\sigma \sigma ^{\intercal } (x-z)\delta }( y) \big ) \int _0^1 y_i f(x-z-\theta y) \,d\theta dy \,dz. \end{aligned}$$

with summation over *i* implied. It is well known that

6.5 $$\begin{aligned} | \partial _{z_i} p_\varepsilon (z)| \leqslant N |z|\varepsilon ^{-1} p_\varepsilon (z). \end{aligned}$$

Furthermore, with the notation $\Sigma (z):= \sigma \sigma ^{\intercal } (x-z)$, we have

6.6 $$\begin{aligned} |\partial _{z_i} p_{ \Sigma (z) \delta }( y)|= & {} \Big | \frac{ \partial _{z_i} ( y^{\intercal } \Sigma ^{-1}(z) y ) }{2\delta } + \frac{\partial _{z_i} \det \Sigma (z) }{ 2 \det \Sigma (z)} \Big | p_{\Sigma (z)\delta }( y) \nonumber \\\leqslant & {} N (\delta ^{-1}|y|^2+1) p_{\Sigma (z)\delta }( y), \end{aligned}$$

where for the last inequality we have used (2.8). Therefore, by (6.4), (6.5), and (6.6) we see that

$$\begin{aligned} \Vert \mathcal {P}_\varepsilon g\Vert _{\mathcal {C}^0}&\leqslant N\Vert f\Vert _{\mathcal {C}^0} \int _{\mathbb {R}^d}\int _{\mathbb {R}^d}\Big (\varepsilon ^{-1}|z|+\delta ^{-1}|y|^2+1\Big ) \Big ( |y| p_\varepsilon (z) p_{\sigma \sigma ^{\intercal } (x-z)\delta }( y)\Big )\,dy\,dz \\&\leqslant N|f\Vert _{\mathcal {C}^0}(\varepsilon ^{-1/2}\delta ^{1/2}+\delta ^{1/2}) \leqslant N\Vert f\Vert _{\mathcal {C}^0}\varepsilon ^{-1/2}n^{-1/2}. \end{aligned}$$

One also has the trivial estimate $\Vert \mathcal {P}_\varepsilon g\Vert _{\mathcal {C}^0}\leqslant 2 \Vert f\Vert _{\mathcal {C}^0}$, and combining these two bounds yields

6.7 $$\begin{aligned} \Vert g\Vert _{\mathcal {C}^\beta }\leqslant N\Vert f\Vert _{\mathcal {C}^0} n^{\beta /2}. \end{aligned}$$

for all $\beta \in [-1,0)$. Note that the restriction of ${\bar{X}}^n_t(\cdot )$ to the gridpoints $t=0,1/n,\ldots ,1$ is a Markov process with state space $\mathbb {R}^d$. Therefore we can write

6.8 $$\begin{aligned} |\;\;{\mathbb {E}}\;^{(k+1)/n}g\big ({\bar{X}}^n_{\kappa _n(r)}(y)\big )|&=|\;\;{\mathbb {E}}\;g\big ({\bar{X}}^n_{\kappa _n(r)-(k+1)/n}(x)\big )|\Big |_{x={\bar{X}}^n_{(k+1)/n}(y)} \nonumber \\&\leqslant \sup _{x\in \mathbb {R}^d} |\;\;{\mathbb {E}}\;g\big ({\bar{X}}^n_{\kappa _n(r)-(k+1)/n}(x)\big )|. \end{aligned}$$

Since $g \in \mathcal {C}^{\alpha /2}$ we have that $(I+\Delta )u= g$ where $u \in \mathcal {C}^{2+(\alpha /2)}$ and

6.9 $$\begin{aligned} \Vert u\Vert _{\mathcal {C}^{2+(\alpha /2)}} \leqslant N \Vert g\Vert _{\mathcal {C}^{\alpha /2}}, \qquad \Vert u\Vert _{\mathcal {C}^{{1+2\varepsilon _1}}} \leqslant N \Vert g\Vert _{\mathcal {C}{-1+2\varepsilon _1}}. \end{aligned}$$

Hence, by combining (6.8), (5.2), (6.9), (6.7), and (6.3), we get

$$\begin{aligned} |\;\;{\mathbb {E}}\;^{(k+1)/n}g\big ({\bar{X}}^n_{\kappa _n(r)}(y)\big )|\leqslant & {} \sup _{x\in \mathbb {R}^d} |\;\;{\mathbb {E}}\;(u+\Delta u) \big ({\bar{X}}^n_{\kappa _n(r)-(k+1)/n}(x)\big )| \\\leqslant & {} N \Vert u \Vert _{\mathcal {C}^1} |\kappa _n(r)-(k+1)/n|^{-1/2}+N \Vert u \Vert _{\mathcal {C}^2} e^{-cn} \\\leqslant & {} N \Vert u \Vert _{\mathcal {C}^{1+2\varepsilon _1}} |\kappa _n(r)-(k+1)/n|^{-1/2}+N \Vert u \Vert _{\mathcal {C}^2} e^{-cn} \\\leqslant & {} N \Vert g\Vert _{\mathcal {C}^{-1+2\varepsilon _1}} |\kappa _n(r)-(k+1)/n|^{-1/2}+N \Vert g \Vert _{\mathcal {C}^{\alpha /2}} e^{-cn} \\\leqslant & {} N \Vert f\Vert _{\mathcal {C}^\alpha } n^{-1/2+\varepsilon _1}|\kappa _n(r)-(k+1)/n|^{-1/2} \end{aligned}$$

Putting this back into (6.2) one obtains

$$\begin{aligned} \Vert I_2\Vert _{L_p(\Omega )}\leqslant & {} N\Vert f\Vert _{\mathcal {C}^0}n^{-1/2+\varepsilon _1}\int _{(k+4)/n}^t|\kappa _n(r)-(k+1)/n|^{-1/2}\,dr \\\leqslant & {} N\Vert f\Vert _{\mathcal {C}^\alpha }|t-s|^{1/2}n^{-1/2+\varepsilon _1} \\\leqslant & {} N\Vert f\Vert _{\mathcal {C}^\alpha }|t-s|^{1/2+\varepsilon _1}n^{-1/2+2\varepsilon _1}, \end{aligned}$$

where we have used that $n^{-1} \leqslant |t-s|$. The bound for $I_1$ is straightforward:

$$\begin{aligned} \Vert I_1\Vert _{L_p(\Omega )}\leqslant & {} \int _s^{(k+4)/n} \Vert f({\bar{X}}_r)-f({\bar{X}}_{k_n(r)}) \Vert _{L_p(\Omega )} \, dr \\\leqslant & {} N \Vert f\Vert _{\mathcal {C}^0}n^{-1} \leqslant N \Vert f\Vert _{\mathcal {C}^0} n^{-1/2+\varepsilon _1}|t-s|^{1/2+\varepsilon _1}. \end{aligned}$$

Therefore,

$$\begin{aligned} \Vert A_{s,t}\Vert _{L_p(\Omega )}\leqslant N \Vert f\Vert _{\mathcal {C}^\alpha } n^{-1/2+2\varepsilon _1}|t-s|^{1/2+\varepsilon _1}. \end{aligned}$$

It remains to show the same bound for $t \in (s, (k+4)/n]$. Similarly to the above we write

$$\begin{aligned} \Vert A_{s,t}\Vert _{L_p(\Omega )}\leqslant & {} \int _s^t \Vert f({\bar{X}}_r)-f({\bar{X}}_{k_n(r)}) \Vert _{L_p(\Omega )} \, dr \\\leqslant & {} N \Vert f\Vert _{\mathcal {C}^0} |t-s| \leqslant N \Vert f\Vert _{\mathcal {C}^0} n^{-1/2+\varepsilon _1}|t-s|^{1/2+\varepsilon _1}. \end{aligned}$$

using that $|t-s|\leqslant 4 n^{-1}$ and $\varepsilon _1<1/2$. Thus, (3.8) holds with $C_1=N \Vert f\Vert _{\mathcal {C}^\alpha } n^{-1/2+2\varepsilon _1}$. From here we conclude the bound (6.1) exactly as is Lemma 4.1. $\square$

#### Lemma 6.2

Let $\alpha \in [0,1]$, take $\varepsilon _1\in (0,1/2)$. Let $b\in \mathcal {C}^0$, $\sigma$ satisfy (2.8), and $X^n$ be the solution of (1.7). Then for all $f\in \mathcal {C}^\alpha$, $0\leqslant s\leqslant t\leqslant 1$, $n\in \mathbb {N}$, and $p>0$, one has the bound

6.10 $$\begin{aligned} \big \Vert \int _s^t (f(X_r^n)-f(X_{\kappa _n(r)}^n))\, dr\big \Vert _{L_p(\Omega )} \leqslant N\Vert f\Vert _{\mathcal {C}^\alpha } n^{-1/2+2\varepsilon _1}|t-s|^{1/2+\varepsilon _1} \end{aligned}$$

with some $$N=N(\Vert b\Vert _{\mathcal {C}^0},p, d,\alpha ,\varepsilon _1, \lambda ,\Vert \sigma \Vert _{\mathcal {C}^2})$$.

#### Proof

Let us set

$$\begin{aligned} \rho = \exp \left( -\int _0^1 (\sigma ^{-1}b)(X_{\kappa _n(r)}^n) \, dB_r - \frac{1}{2}\int _0^1 \big |(\sigma ^{-1}b)(X_{\kappa _n(r)}^n)\big |^2 \, dr \right) \end{aligned}$$

and define the measure ${\tilde{\mathbb {P}}}$ by $d {\tilde{\mathbb {P}}} = \rho d \mathbb {P}$. By Girsanov’s theorem, $X^n$ solves (5.1) with a ${\tilde{\mathbb {P}}}$-Wiener process ${{\tilde{B}}}$ in place of *B*. Since Lemma 6.1 only depends on the distribution of ${\bar{X}}^n$, we can apply it to $X^n$, to bound the desired moments with respect to the measure ${\tilde{\mathbb {P}}}$. Going back to the measure $\mathbb {P}$ can then be done precisely as in [10]: the only property needed is that $\rho$ has finite moments of any order, which follows easily from the boundedness of *b* and (2.8). $\square$

### A regularization lemma

The replacement for the heat kernel bounds from Proposition 3.7 is the following estimate on the transition kernel ${\bar{P}}$ of (1.6). Similarly to before, we denote ${\bar{P}}_t f(x)=\;\;{\mathbb {E}}\;f(X_t(x))$, where $X_t(x)$ is the solution of (1.6) with initial condition $X_0(x)=x$. The following bound then follows from [16, Theorem 9/4/2].

#### Proposition 6.3

Assume $b\in \mathcal {C}^\alpha$, $\alpha >0$ and $f\in \mathcal {C}^{\alpha '}$, $\alpha '\in [0,1]$. Then for all $0< t\leqslant 1$, $x,y\in \mathbb {R}^d$ one has the bounds

6.11 $$\begin{aligned} |{\bar{\mathcal {P}}}_tf(x)-{\bar{\mathcal {P}}}_tf(y)|\leqslant N\Vert f\Vert _{\mathcal {C}^{\alpha '}}|x-y| t^{-(1-\alpha ')/2} \end{aligned}$$

with some $$N=N(d,\alpha ,\lambda ,\Vert b\Vert _{\mathcal {C}^\alpha },\Vert \sigma \Vert _{\mathcal {C}^1})$$.

#### Lemma 6.4

Let $\alpha \in (0,1]$ and $\tau \in (0,1]$ satisfy

6.12 $$\begin{aligned} \tau +\alpha /2-1/2>0. \end{aligned}$$

Let $b\in \mathcal {C}^\alpha$, $\sigma$ satisfy (2.8), and *X* be the solution of (1.6). Let $\varphi$ be an adapted process. Then for all sufficiently small $\varepsilon _3,\varepsilon _4>0$, for all $f\in \mathcal {C}^\alpha$, $0\leqslant s\leqslant t\leqslant 1$, and $p>0$, one has the bound

6.13 $$\begin{aligned}&\big \Vert \int _s^t f(X_r) -f(X_r+\varphi _{r}) \,dr\big \Vert _{L_p(\Omega )} \leqslant N |t-s|^{1+\varepsilon _3} [] \varphi []_{\mathscr {C}^\tau _p,[s,t]}\nonumber \\&\quad + N|t-s|^{1/2+\varepsilon _4}[] \varphi []_{\mathscr {C}^0_p,[s,t]}. \end{aligned}$$

with some $$N=N(p,d,\alpha ,\tau ,\lambda ,\Vert \sigma \Vert _{\mathcal {C}^1}).$$

#### Proof

Set, for $$s\leqslant s'\leqslant t'\leqslant t$$,

$$\begin{aligned} A_{s',t'}=\;\;{\mathbb {E}}\;^{s'}\int _{s'}^{t'} f(X_r)-f(X_r+\varphi _{s'})\,dr. \end{aligned}$$

Let us check the conditions of the stochastic sewing lemma. We have

$$\begin{aligned} \delta A_{s',u,t'}=\;\;{\mathbb {E}}\;^{s'} \int _{u}^{t'} (f(X_r)-f(X_r+\varphi _{s'}))\, dr-\;\;{\mathbb {E}}\;^u \int _u^{t'}(f(X_r)-f(X_r+\varphi _u))\, dr, \end{aligned}$$

so $$\;\;{\mathbb {E}}\;^{s'}\delta A_{s',u,t'}=\;\;{\mathbb {E}}\;^{s'}\hat{\delta }A_{s',u,t'}$$, with

$$\begin{aligned} {\hat{\delta }} A_{s',u,t'}= & {} \;\;{\mathbb {E}}\;^u\int _u^{t'}\big (f(X_r)-f(X_r+\varphi _{s'})\big )-\big (f(X_r)+f(X_r+\varphi _u)\big )\,dr \\= & {} \int _u^{t'} {\bar{\mathcal {P}}}_{r-u}f(X_u+\varphi _{s'})-{\bar{\mathcal {P}}}_{r-u}f(X_u+\varphi _u)\,dr. \end{aligned}$$

Invoking (6.11), we can write

$$\begin{aligned} |{\hat{\delta }} A_{s',u,t'}|&\leqslant N \int _{u}^{t'}|\varphi _{s'}-\varphi _u||r-u|^{-(1-\alpha )/2}\,dr. \end{aligned}$$

Hence, using also Jensen’s inequality,

$$\begin{aligned} \Vert \;\;{\mathbb {E}}\;^{s'}\delta A_{s',u,t'}\Vert _{L_p(\Omega )} \leqslant \Vert {\hat{\delta }} A_{s',u,t'}\Vert _{L_p(\Omega )}&\leqslant N[] \varphi []_{\mathscr {C}^\tau _p,[s,t]}|t'-s'|^{1+\tau -(1-\alpha )/2} \end{aligned}$$

The condition (6.12) implies that for some $\varepsilon _3>0$, one has

$$\begin{aligned} \Vert \;\;{\mathbb {E}}\;^{s'}\delta A_{s',u,t'}\Vert _{L_p(\Omega )} \leqslant N |t'-s'|^{1+\varepsilon _3} [] \varphi []_{\mathscr {C}^\tau _p,[s,t]}. \end{aligned}$$

Therefore (3.9) is satisfied with $C_2=N[] \varphi []_{\mathscr {C}^\tau _p,[s,t]}$. Next, to bound $\Vert A_{s',t'}\Vert _{L_p(\Omega )}$, we write

$$\begin{aligned} |\;\;{\mathbb {E}}\;^s f(X_r)- \;\;{\mathbb {E}}\;^s f(X_r+\varphi _{s'})|= & {} |{\bar{\mathcal {P}}}_{r-s'}f(X_{s'})-{\bar{\mathcal {P}}}_{r-s'}f(X_{s'}+\varphi _{s'})| \\\leqslant & {} N |\varphi _{s'}||r-s'|^{-(1-\alpha )/2}. \end{aligned}$$

So after integration with respect to *r* and by Jensen’s inequality, we get the bound, for any sufficiently small $\varepsilon _4>0$,

$$\begin{aligned} \Vert A_{s',t'}\Vert _{L_p(\Omega )}\leqslant N|t'-s'|^{1/2+\varepsilon _4}[] \varphi []_{\mathscr {C}^0_p,[s,t]}. \end{aligned}$$

Therefore (3.8) is satisfied with $C_1=N[] \varphi []_{\mathscr {C}^0_p,[s,t]}$, and we can conclude the bound (6.1) as usual. $\square$

### Proof of Theorem 2.7

First let us recall the following simple fact: if *g* is a predictable process, then by the Burkholder-Gundy-Davis and Hölder inequalities one has

$$\begin{aligned} \;\;{\mathbb {E}}\;\big |\int _s^t g_r\,dB_r\big |^p\leqslant N\;\;{\mathbb {E}}\;\int _s^t|g_r|^p\,dr|t-s|^{(p-2)/2}\end{aligned}$$

with $N=N(p)$. This in particular implies

6.14 $$\begin{aligned}{}[] g []_{\mathscr {C}^{1/2-\varepsilon }_p,[s,t]}\leqslant N \Vert g\Vert _{L_p(\Omega \times [s,t])}. \end{aligned}$$

whenever $$p\geqslant 1/\varepsilon $$.

#### Proof

Without the loss of generality we will assume that *p* is sufficiently large and $\tau$ is sufficiently close to 1/2. Let us rewrite the equation for $X^n$ as

$$\begin{aligned} dX^n_t=b(X^n_{\kappa _n(t)})\,dt+\big [\sigma (X_t)+(\sigma (X^n_t)-\sigma (X_t))+R^n_r\big ]\,dB_t, \end{aligned}$$

where $R^n_t=\sigma (X^n_{\kappa _n(t)})-\sigma (X^n_t)$ is an adapted process such that one has

$$\begin{aligned} \Vert R^n_t\Vert _{L_p(\Omega )}\leqslant N n^{-1/2} \end{aligned}$$

for all $t\in [0,1]$. Let us denote

$$\begin{aligned} -\varphi ^n_t= & {} x_0-x^n_0+\int _0^t b(X_r)\,dr-\int _0^t b(X_{\kappa _n(r)}^n)\,dr, \\ \mathcal {Q}^n_t= & {} \int _0^t\sigma (X^n_r)-\sigma (X_r)\,dB_r, \\ \mathcal {R}^n_t= & {} \int _0^tR^n_r\,dB_r. \end{aligned}$$

Take some $0\leqslant S\leqslant T\leqslant 1$. Choose $\varepsilon _1\in (0,\varepsilon /2)$ so that $(1/2-2\varepsilon _1)\geqslant 1/2-\varepsilon$. Then, taking into account (6.10), for any $S\leqslant s< t\leqslant T$, we have

6.15 $$\begin{aligned} \Vert \varphi ^n_t-\varphi ^n_s\Vert _{L_p(\Omega )}= & {} \big \Vert \int _s^t (b(X_r)-b(X^n_{\kappa _n(r)}))\,dr\big \Vert _{L_p(\Omega )}\nonumber \\\leqslant & {} \big \Vert \int _s^t (b(X_r)-b(X^n_r))\,dr\big \Vert _{L_p(\Omega )}+N|t-s|^{1/2+\varepsilon _1} n^{-1/2+\varepsilon }.\nonumber \\ \end{aligned}$$

We wish to apply Lemma 6.4, with $\varphi =\varphi ^n+\mathcal {Q}^n+\mathcal {R}^n$. It is clear that for sufficiently small $\varepsilon _2>0$, $\tau =1/2-\varepsilon _2$ satisfies (6.12). Therefore,

$$\begin{aligned}&\big \Vert \int _s^t (b(X_r)-b(X^n_r))\,dr\big \Vert _{L_p(\Omega )} =\big \Vert \int _s^t (b(X_r)-b(X_r+\varphi _r))\,dr\big \Vert _{L_p(\Omega )} \\&\quad \leqslant N|t-s|^{1/2+\varepsilon _4\wedge (1/2+\varepsilon _3)}\big ([] \varphi ^n []_{\mathscr {C}^\tau _p,[s,t]} +[] \mathcal {Q}^n []_{\mathscr {C}^\tau _p,[s,t]} +[] \mathcal {R}^n []_{\mathscr {C}^\tau _p,[s,t]}\big ) \end{aligned}$$

By (6.14), for sufficiently large *p*, we have

$$\begin{aligned}{}[] \mathcal {Q}^n []_{\mathscr {C}^\tau _p,[s,t]}\leqslant & {} N\Vert X-X^n\Vert _{L_p(\Omega \times [0,T])}, \\ [] \mathcal {R}^n []_{\mathscr {C}^\tau _p,[s,t]}\leqslant & {} Nn^{-1/2}. \end{aligned}$$

Putting these in the above expression, and using $\tau <1/2$ repeatedly, one gets

$$\begin{aligned}&\big \Vert \int _s^t (b(X_{r})-b(X^n_{r}))\,dr\big \Vert _{L_p(\Omega )} \\&\quad \leqslant N |t-s|^{\tau }|T-S|^{\varepsilon _5} \big ([] \varphi ^n []_{\mathscr {C}^\tau _p,[S,T]}+\Vert X-X^n\Vert _{L_p(\Omega \times [0,T])}+n^{-1/2}\big ) \end{aligned}$$

with some $\varepsilon _5>0$. Combining with (6.15), dividing by $|t-s|^\tau$ and taking supremum over $s<t\in [S,T]$, we get

6.16 $$\begin{aligned}{}[] \varphi ^n []_{\mathscr {C}^\tau _p,[S,T]}&\leqslant N\Vert \varphi ^n_S\Vert _{L_p(\Omega )}+|T-S|^{\varepsilon _5}[] \varphi ^n []_{\mathscr {C}^\tau _p,[S,T]} \nonumber \\&\qquad +N\Vert X-X^n\Vert _{L_p(\Omega \times [0,T])}+Nn^{-1/2+\varepsilon }. \end{aligned}$$

Fix an $m\in \mathbb {N}$ (not depending on *n*) such that $Nm^{-\varepsilon _5}\leqslant 1/2$. Whenever $|S-T|\leqslant m^{-1}$, the second term on the right-hand side of (6.16) can be therefore discarded, and so one in particular gets

6.17 $$\begin{aligned}{}[] \varphi ^n []_{\mathscr {C}^\tau _p,[S,T]} \leqslant N\Vert \varphi ^n_S\Vert _{L_p(\Omega )}+N\Vert X-X^n\Vert _{L_p(\Omega \times [0,T])}+Nn^{-1/2+\varepsilon }, \end{aligned}$$

and thus also

$$\begin{aligned} \Vert \varphi ^n_{T}\Vert _{L_p(\Omega )} \leqslant N\Vert \varphi ^n_S\Vert _{L_p(\Omega )}+N\Vert X-X^n\Vert _{L_p(\Omega \times [0,T])}+Nn^{-1/2+\varepsilon }. \end{aligned}$$

Iterating this inequality at most *m* times, one therefore gets

6.18 $$\begin{aligned} \Vert \varphi ^n_T\Vert _{L_p(\Omega )} \leqslant N\Vert \varphi ^n_0\Vert _{L_p(\Omega )}+N\Vert X-X^n\Vert _{L_p(\Omega \times [0,T])}+Nn^{-1/2+\varepsilon }. \end{aligned}$$

We can then write, invoking again the usual estimates for the stochastic integrals $\mathcal {Q}^n$, $\mathcal {R}^n$

$$\begin{aligned} \sup _{t\in [0,T]}\big \Vert X_t-X_t^n\big \Vert _{L_p(\Omega )}^p\leqslant & {} N\sup _{t\in [0,T]}\big \Vert \varphi ^n_t\big \Vert _{L_p(\Omega )}^p \\&\quad +N\sup _{t\in [0,T]}\big \Vert \mathcal {Q}^n_t\big \Vert _{L_p(\Omega )}^p +N\sup _{t\in [0,T]}\big \Vert \mathcal {R}^n_t\big \Vert _{L_p(\Omega )}^p \\\leqslant & {} N\Vert \varphi ^n_0\Vert _{L_p(\Omega )}^p+N\int _0^T\Vert X_t-X^n_t\Vert _{L_p(\Omega )}^p\,dt+Nn^{-p(1/2-\varepsilon )}. \end{aligned}$$

Gronwall’s lemma then yields

6.19 $$\begin{aligned} \sup _{t\in [0,T]}\big \Vert X_t-X_t^n\big \Vert _{L_p(\Omega )}\leqslant N\Vert \varphi ^n_0\Vert _{L_p(\Omega )}+Nn^{-1/2+\varepsilon }. \end{aligned}$$

Putting (6.17)–(6.18)–(6.19) together, we obtain

6.20 $$\begin{aligned}{}[] \varphi ^n []_{\mathscr {C}^\tau _p,[0,1]} \leqslant N\Vert \varphi ^n_0\Vert _{L_p(\Omega )}+Nn^{-1/2+\varepsilon }. \end{aligned}$$

Therefore, recalling (6.14) again,

$$\begin{aligned}{}[] X-X^n []_{\mathscr {C}^\tau _p,[0,1]}\leqslant & {} [] \varphi ^n []_{\mathscr {C}^\tau _p,[0,1]} +[] \mathcal {Q}^n []_{\mathscr {C}^\tau _p,[0,1]} +[] \mathcal {R}^n []_{\mathscr {C}^\tau _p,[0,1]} \\\leqslant & {} N\Vert \varphi ^n_0\Vert _{L_p(\Omega )}+Nn^{-1/2+\varepsilon }+\sup _{t\in [0,1]}\big \Vert X_t-X_t^n\big \Vert _{L_p(\Omega )} \\\leqslant & {} N\Vert \varphi ^n_0\Vert _{L_p(\Omega )}+Nn^{-1/2+\varepsilon }, \end{aligned}$$

as desired. $$\square $$

## A: Proofs of the auxiliary bounds from Section 3.3

### Proof of Proposition 3.6

(i). Fix $0\leqslant s\leqslant t\leqslant 1$. It follows from the definition of $B^H$ that $B^H_t-B^H_s$ is a Gaussian vector consisting of *d* independent components, each of them having zero mean and variance

$$\begin{aligned} C(t,t)-2C(s,t)+C(s,s)=c_H(t-s)^{2H}, \end{aligned}$$

where the function *C* was defined in (2.2). This implies the statement of the proposition.

(ii). We have

$$\begin{aligned} \;\;{\mathbb {E}}\;^uB_t^{H,i}-\;\;{\mathbb {E}}\;^sB_t^{H,i}=\int _s^u (t-r)^{H-1/2} dW_r^i. \end{aligned}$$

Therefore, $\;\;{\mathbb {E}}\;^sB_t^{H,i}-\;\;{\mathbb {E}}\;^uB_t^{H,i}$ is a Gaussian random variable independent of $\mathcal {F}_s$. It is of mean 0 and variance $c^2(s,t)-c^2(u,t)$. This implies the statement of the lemma.

(iii). It suffices to notice that the random vector $B^H_t-\;\;{\mathbb {E}}\;^s B^H_t$ is Gaussian, independent of $\mathcal {F}_s$, consists of *d* independent components, and each of its components has zero mean and variance

$$\begin{aligned} \;\;{\mathbb {E}}\;\big (\int _s^t|t-r|^{H-1/2}\,dW_r\big )^2=c^2(s,t). \end{aligned}$$

(iv). One can simply write by the Newton-Leibniz formula

$$\begin{aligned} c^2(s,t)-c^2(s,u)\leqslant N \int _u^t |r-s|^{2H-1}\,dr\leqslant N |t-u||t-s|^{2H-1}, \end{aligned}$$

since by our assumption on *s*, *u*, *t*, for all $r\in [u,t]$ one has $r-s\leqslant t-s\leqslant 2(r-s)$.

(v). It follows from (2.1) that

$$\begin{aligned} \;\;{\mathbb {E}}\;^sB^H_t-\;\;{\mathbb {E}}\;^sB^H_u=\int _{-\infty }^s (|t-r|^{H-1/2}-|u-r|^{H-1/2})\,dW_r. \end{aligned}$$

Therefore, by the Burkholder–Davis–Gundy inequality one has

$$\begin{aligned} \Vert \;\;{\mathbb {E}}\;^sB^H_t-\;\;{\mathbb {E}}\;^sB^H_r\Vert _{L_p(\Omega )}^2\leqslant & {} N \int _{-\infty }^s \bigl (|t-r|^{H-1/2}-|u-r|^{H-1/2}\bigr )^2\,dr\\\leqslant & {} N\int _{-\infty }^s\Bigl (\int _u^t|v-r|^{H-3/2}\,dv\Bigr )^2\,dr \\\leqslant & {} N\int _{-\infty }^s|t-u|^2|u-r|^{2H-3}\,dr \\\leqslant & {} N(t-u)^2 (u-s)^{2H-2}\leqslant N(t-u)^2 (t-s)^{2H-2}, \end{aligned}$$

where the last inequality follows from the fact that by the assumption $u-s\geqslant (t-s)/2$. $\square$

### Proof of Proposition 3.7

(i). *Case* $\beta \leqslant \alpha$: There is nothing to prove since

$$\begin{aligned} \Vert \mathcal {P}_tf\Vert _{\mathcal {C}^\beta (\mathbb {R}^d)}\leqslant \Vert \mathcal {P}_tf\Vert _{\mathcal {C}^\alpha (\mathbb {R}^d)}\leqslant N\Vert f\Vert _{\mathcal {C}^\alpha (\mathbb {R}^d)}. \end{aligned}$$

*Case* $\beta =0$, $\alpha <0$: The bound follows immediately from the definition of the norm.

*Case* $\alpha =0$, $\beta \in (0, 1]$: By differentiating the Gaussian density we have

$$\begin{aligned} \Vert \nabla \mathcal {P}_t f\Vert _{\mathcal {C}^0} \leqslant N t^{-1/2} \Vert f\Vert _{\mathcal {C}^0}. \end{aligned}$$

Consequently,

$$\begin{aligned} | \mathcal {P}_tf(x)-\mathcal {P}_tf(y)|\leqslant & {} | \mathcal {P}_tf(x)-\mathcal {P}_tf(y)|^\beta \Vert f\Vert _{\mathcal {C}^0}^{(1-\beta )} \\\leqslant & {} N t^{-\beta /2} |x-y|^\beta \Vert f\Vert _{\mathcal {C}^0}, \end{aligned}$$

which implies that

$$\begin{aligned}{}[\mathcal {P}_t f]_{\mathcal {C}^\beta } \leqslant N t^{-\beta /2} \Vert f\Vert _{\mathcal {C}^0}. \end{aligned}$$

This, combined with the trivial estimate $\Vert \mathcal {P}_tf\Vert _{\mathcal {C}^0} \leqslant \Vert f\Vert _{L_\infty }$ give the desired estimate.

*Case* $0< \alpha< \beta <1$: We refer the reader to [17, Lemma A.7] where the estimate is proved in the Besov scale. The desired estimate then follows from the equivalence $\mathcal {B}^\gamma _{\infty , \infty } \sim \mathcal {C}^\gamma$ for $\gamma \in (0,1)$.

*Case* $\alpha \in (0,1)$, $\beta =1$: We have

$$\begin{aligned} \Vert \nabla \mathcal {P}_t f \Vert _{L\infty }= & {} \sup _{x \in \mathbb {R}^d} \Big | \int _{\mathbb {R}^d} \nabla p_t(x-y)f(y) \, dy \Big | \\= & {} \sup _{x \in \mathbb {R}^d} \Big | \int _{\mathbb {R}^d} \nabla p_t(x-y)\big (f(y)-f(x) \big ) \, dy \Big | \\\leqslant & {} N [f]_{\mathcal {C}^\alpha } \int _{\mathbb {R}^d} | \nabla p_t(y)| |y|^\alpha \, dy \\\leqslant & {} N [f]_{\mathcal {C}^\alpha } t^{(\alpha -1)/2}, \end{aligned}$$

which again combined with $\Vert \mathcal {P}_tf\Vert _{\mathcal {C}^0} \leqslant \Vert f \Vert _{\mathcal {C}^0}$ proves the claim.

*Case* $$\alpha < 0$$, $$\beta \in [0,1]$$:

$$\begin{aligned} \Vert \mathcal {P}_t f\Vert _{\mathcal {C}^\beta } = \Vert \mathcal {P}_{\frac{t}{2}+\frac{t}{2}} f\Vert _{\mathcal {C}^\beta } \leqslant N t^{-\beta /2} \Vert \mathcal {P}_{t/2}f\Vert _{\mathcal {C}^0}\leqslant & {} N t^{(\alpha -\beta )/2} \sup _{\varepsilon \in (0,1]} \varepsilon ^{-\alpha /2}\Vert \mathcal {P}_\varepsilon f\Vert _{\mathcal {C}^0} \\= & {} N t^{(\alpha -\beta )/2} \Vert f\Vert _{\mathcal {C}^\alpha }. \end{aligned}$$

(ii). Fix $\delta \in (0,1]$ such that $\delta \geqslant {\frac{\alpha }{2}-\frac{\beta }{2}}$. Then we have

$$\begin{aligned} \Vert \mathcal {P}_tf-\mathcal {P}_sf\Vert _{\mathcal {C}^{\beta }(\mathbb {R}^d)}&\leqslant \int _s^t \Bigl \Vert \frac{\partial }{\partial r}\mathcal {P}_rf\Bigr \Vert _{\mathcal {C}^{\beta }(\mathbb {R}^d)}\,dr\\&=\int _s^t \Bigl \Vert \mathcal {P}_r \Delta f\Bigr \Vert _{\mathcal {C}^{\beta }(\mathbb {R}^d)}\,dr\\&\leqslant N\int _s^t r^{\frac{\alpha -\beta -2}{2}} \Bigl \Vert \Delta f\Bigr \Vert _{\mathcal {C}^{\alpha -2}(\mathbb {R}^d)}\,dr\\&\leqslant N\Vert f\Vert _{\mathcal {C}^{\alpha }(\mathbb {R}^d)}\int _s^t r^{\frac{\alpha }{2}-\frac{\beta }{2}-\delta }r^{-1+\delta }\,dr\\&\leqslant N\Vert f\Vert _{\mathcal {C}^{\alpha }(\mathbb {R}^d)}s^{\frac{\alpha }{2}-\frac{\beta }{2}-\delta }(t-s)^{\delta }, \end{aligned}$$

where the last inequality follows from the facts that $r\geqslant s$ and $r\geqslant r-s$, and that both of the exponents in the penultimate inequality are nonpositive thanks to the conditions on $\delta$. This yields the statement of (ii).

(iii). First let us deal with the case $H\leqslant 1/2$. Then the bound follows easily by applying part (ii) of the proposition with $\delta =1/2$. Indeed, for any $0\leqslant s\leqslant u \leqslant t$ we have

$$\begin{aligned}&\Vert \mathcal {P}_{c^2(s,t)}f-\mathcal {P}_{c^2(u,t)}f\Vert _{\mathcal {C}^\beta }\leqslant N \Vert f\Vert _{\mathcal {C}^\alpha } c^{\alpha -\beta -1}(u,t) (c^2(s,t)-c^2(u,t))^{\frac{1}{2}}\\&\leqslant N\Vert f\Vert _{\mathcal {C}^\alpha }(t-u)^{H(\alpha -\beta -1)}(u-s)^{\frac{1}{2}} (t-u)^{H-\frac{1}{2}}\\&=N\Vert f\Vert _{\mathcal {C}^\alpha }(u-s)^{\frac{1}{2}} (t-u)^{H(\alpha -\beta )-\frac{1}{2}}, \end{aligned}$$

where we also used the fact that

A.1 $$\begin{aligned} c^2(s,t)-c^2(u,t)\leqslant N (u-s)(t-u)^{2H-1}. \end{aligned}$$

This establishes the desired bound.

Now let us consider the case $H>1/2$ (in this case $2H-1>0$ and thus bound (A.1) does not hold). Put for $0\leqslant s\leqslant u \leqslant t$

A.2 $$\begin{aligned} k(s,u,t):= & {} c^2(u,t)+(u-s)\partial _tc^2(u,t)\nonumber \\= & {} (2H)^{-1}(t-u)^{2H}+(u-s)(t-u)^{2H-1}. \end{aligned}$$

Note that by convexity of the function $z\mapsto z^{2H}$ one has for any $0\leqslant z_1\leqslant z_2$

$$\begin{aligned} z_1^{2H}+2H(z_2-z_1)z_1^{2H-1}\leqslant z_2^{2H} \leqslant z_1^{2H}+2H(z_2-z_1)z_1^{2H-1}+(z_2-z_1)^{2H}. \end{aligned}$$

Hence for $0\leqslant s\leqslant u \leqslant t$ we have

A.3 $$\begin{aligned} c^2(u,t)\leqslant k(s,u,t)\leqslant c^2(s,t) \leqslant k(s,u,t)+c^2(s,u) \end{aligned}$$

Now we are ready to obtain the desired bound. We have

A.4 $$\begin{aligned} \Vert \mathcal {P}_{c^2(s,t)}f-\mathcal {P}_{c^2(u,t)}f\Vert _{\mathcal {C}^\beta }&\leqslant \Vert \mathcal {P}_{c^2(s,t)}f-\mathcal {P}_{k(s,u,t)}f\Vert _{\mathcal {C}^\beta } +\Vert \mathcal {P}_{k(s,u,t)}f-\mathcal {P}_{c^2(u,t)}f\Vert _{\mathcal {C}^\beta }\nonumber \\&\leqslant I_1+I_2. \end{aligned}$$

We bound $I_1$ and $I_2$ using part (ii) of the proposition but with different $\delta$. First, we apply part (ii) with $\delta =\frac{1}{4H}\vee (\alpha /2-\beta /2)$. Recalling (A.3), we deduce

A.5 $$\begin{aligned} I_1\leqslant N\Vert f\Vert _{C^\alpha }k(s,u,t)^{\frac{\alpha }{2}-\frac{\beta }{2}-\delta }c^{2\delta }(s,u) \leqslant N\Vert f\Vert _{C^\alpha }(u-s)^{\frac{1}{2}}(t-u)^{(H(\alpha -\beta )-\frac{1}{2})\wedge 0}.\nonumber \\ \end{aligned}$$

Applying now part (ii) with $\delta =1/2$, we obtain

$$\begin{aligned} I_2\leqslant N \Vert f\Vert _{C^\alpha }c^{\alpha -\beta -1}(u,t)(u-s)^{\frac{1}{2}}(t-u)^{H-\frac{1}{2}}\leqslant N \Vert f\Vert _{C^\alpha } (u-s)^{\frac{1}{2}} (t-u)^{H(\alpha -\beta )-\frac{1}{2}}. \end{aligned}$$

This, combined with (A.4) and (A.5) implies the desired bound for the case $H>1/2$.

(iv). We begin with the case $H\leqslant 1/2$. Then, applying part (i) of the theorem with $\beta =1$, we deduce for $0\leqslant s \leqslant u \leqslant t\leqslant 1$

$$\begin{aligned} |\mathcal {P}_{c^2(u,t)}f(x)-\mathcal {P}_{c^2(u,t)}f(x+\xi )|\leqslant N\Vert f\Vert _{C^\alpha }(t-u)^{H(\alpha -1)}|\xi |. \end{aligned}$$

Hence for any $p\geqslant 2$ we have

$$\begin{aligned} \Vert \mathcal {P}_{c^2(u,t)}f(x)-\mathcal {P}_{c^2(u,t)}f(x+\xi )\Vert _{L_p(\Omega )}&\leqslant N\Vert f\Vert _{C^\alpha }(t-u)^{H(\alpha -1)}\Vert \xi \Vert _{L_p(\Omega )}\\&\leqslant N\Vert f\Vert _{C^\alpha }(u-s)^{\frac{1}{2}}(t-u)^{H\alpha -\frac{1}{2}}, \end{aligned}$$

where the last inequality follows from the bound (A.1) and the definition of the random variable $\xi$. This completes the proof for the case $H\leqslant 1/2$.

Now let us deal with the case $H\in (1/2,1)$. Fix $0\leqslant s \leqslant u \leqslant t\leqslant 1$. Let $\eta$ and $\rho$ be independent Gaussian random vectors consisting of *d* independent identically distributed components each. Suppose that for any $i=1,\ldots ,d$ we have $\;\;{\mathbb {E}}\;\eta ^i=\;\;{\mathbb {E}}\;\rho ^i=0$ and

$$\begin{aligned} \mathop {\mathrm{Var}}(\eta ^i)=(u-s)(t-u)^{2H-1};\quad \mathop {\mathrm{Var}}(\rho ^i)=v(s,u,t)-(u-s)(t-u)^{2H-1}. \end{aligned}$$

It is clear that

A.6 $$\begin{aligned}&\Vert \mathcal {P}_{c^2(u,t)}f(x)-\mathcal {P}_{c^2(u,t)}f(x+\xi )\Vert _{L_p(\Omega )}\nonumber \\&\quad = \Vert \mathcal {P}_{c^2(u,t)}f(x)-\mathcal {P}_{c^2(u,t)}f(x+\eta +\rho )\Vert _{L_p(\Omega )}\nonumber \\&\quad \leqslant \Vert \mathcal {P}_{c^2(u,t)}f(x)-\mathcal {P}_{c^2(u,t)}f(x+\eta )\Vert _{L_p(\Omega )}\nonumber \\&\qquad + \Vert \mathcal {P}_{c^2(u,t)}f(x+\eta )-\mathcal {P}_{c^2(u,t)}f(x+\eta +\rho )\Vert _{L_p(\Omega )}\nonumber \\&\quad =:I_1+I_2. \end{aligned}$$

Applying part (i) of the theorem with $\beta =1$, we get

A.7 $$\begin{aligned} I_1 \leqslant N\Vert f\Vert _{\mathcal {C}^\alpha }c^{\alpha -1}(u,t)\Vert \eta \Vert _{L_p(\Omega )}\leqslant N\Vert f\Vert _{\mathcal {C}^\alpha }(u-s)^{\frac{1}{2}}(t-u)^{\alpha H-\frac{1}{2}}. \end{aligned}$$

Similarly, using part (i) of the theorem with $\beta =\frac{1}{2H}\vee \alpha$ and recalling (A.3), we deduce

$$\begin{aligned} I_2 \leqslant N\Vert f\Vert _{\mathcal {C}^\alpha }c^{(\alpha -\frac{1}{2H})\wedge 0}(u,t) \,\Vert \,|\rho |^{\frac{1}{2H}\vee \alpha }\,\Vert _{L_p(\Omega )}\leqslant N\Vert f\Vert _{\mathcal {C}^\alpha }(u-s)^{\frac{1}{2}}(t-u)^{(\alpha H-\frac{1}{2})\wedge 0}. \end{aligned}$$

Combined with (A.6) and (A.7), this yields the required bound. $\square$

### Proof of Proposition 3.8

Obviously it suffices to show it for $k=1$.

*1. Case* $\alpha -\delta =0$: The statement follows directly by definition of the $\mathcal {C}^\alpha$ norm.

*2. Case* $\alpha -\delta \in (0,1]$: First, let us consider $\alpha \in (0,1]$. For all $\beta \in [0,1]$ we have

$$\begin{aligned} |f(y+x)-f(y)-f(z+x)-f(z)|&\leqslant (2|x|^\alpha [f]_{\mathcal {C}^\alpha })^ \beta (2|y-z|^{\alpha } [f]_{\mathcal {C}^\alpha })^{(1-\beta )} \end{aligned}$$

which upon dividing by $|y-z|^{\alpha -\delta }$, choosing $\beta = \delta / \alpha$, and taking suprema over $y \ne z$ gives

$$\begin{aligned}{}[f(\cdot +x)-f(\cdot )]_{\mathcal {C}^{\alpha -\delta }} \leqslant 4 |x|^\delta [f]_{\mathcal {C}^\alpha }. \end{aligned}$$

Similarly, we have

$$\begin{aligned} \Vert f(\cdot +x)-f(\cdot )\Vert _{\mathcal {C}^0} \leqslant |x|^\delta [f]_{\mathcal {C}^\alpha }^{\delta /\alpha } (2\Vert f\Vert _{\mathcal {C}^0})^{1-\delta /\alpha } \leqslant 2 |x|^\delta \Vert f\Vert _{\mathcal {C}^\alpha }, \end{aligned}$$

which combined with the inequality above gives

$$\begin{aligned} \Vert f(\cdot +x)-f(\cdot )\Vert _{\mathcal {C}^{\alpha -\delta }} \leqslant 6 |x|^\delta \Vert f\Vert _{\mathcal {C}^\alpha }. \end{aligned}$$

Now let us consider the case $\alpha \in (1,2]$. By the fundamental theorem of calculus we have for any $\beta \in [0,1]$

$$\begin{aligned}&\frac{|f(y+x)-f(y)-f(z+x)-f(z)|}{|y-z|^{\alpha -\delta }} \\&\quad = \frac{1}{|y-z|^{\alpha -\delta }} \Big |\int _0^1 x_i\big ( \partial _{x_i}f(y+\theta x)-\partial _{x_i}f(z+\theta x)\big ) \, d \theta \Big |^\beta \\&\qquad \times \Big |\int _0^1 (y_i-z_i)\big (\partial _{x_i}f(z+x + \theta (y-z))- \partial _{x_i}f(z + \theta (y-z)) \big ) \, d \theta \Big |^{(1-\beta )} \\&\quad \leqslant N \frac{( | x| [\nabla f]_{\mathcal {C}^{\alpha -1}}|y-z|^{\alpha -1} )^\beta (|y-z| [ \nabla f ]_{C^{\alpha -1}} |x|^{\alpha -1})^{1-\beta } }{|y-z|^{\alpha -\delta }} \\&\quad \leqslant N |x|^{\beta +(\alpha -1)(1-\beta )}\Vert f\Vert _{\mathcal {C}^\alpha } |y-z|^{(\alpha -1)\beta +1-\beta -\alpha +\delta }, \end{aligned}$$

which upon choosing $\beta =(\delta +1-\alpha )/2\alpha$ and taking suprema over $y \ne z$ gives

$$\begin{aligned}{}[f(x+\cdot )-f(\cdot )]_{\mathcal {C}^{\alpha -\delta }} \leqslant N |x|^\delta \Vert f\Vert _{\mathcal {C}^\alpha }. \end{aligned}$$

In addition, we have

$$\begin{aligned} \Vert f(\cdot +x)-f(\cdot )\Vert _{\mathcal {C}^0} \leqslant |x|^\delta [f]_{\mathcal {C}^\delta } \leqslant N |x|^\delta \Vert f\Vert _{C^\alpha }, \end{aligned}$$

which combined with the above proves the claim.

*3. Case* $\alpha -\delta \in (k, k+1]$ *for* $k \in \mathbb {N}$: The statement follows by proceeding as above, considering also derivatives of *f* up to sufficiently high order.

*4. Case* $\alpha - \delta <0$: We first consider the case $\alpha \in [0,1)$, for which we have by virtue of Proposition 3.7 (i)

$$\begin{aligned} \Vert f(x+\cdot )- f(\cdot )\Vert _{\mathcal {C}^{\alpha -\delta }}= & {} \sup _{\varepsilon \in (0,1]} \varepsilon ^{\frac{\delta -\alpha }{2}} \Vert \mathcal {P}_\varepsilon f(x+ \cdot )- \mathcal {P}_\varepsilon f(\cdot )\Vert _{\mathcal {C}^0} \\\leqslant & {} \sup _{\varepsilon \in (0,1]} \varepsilon ^{\frac{\delta -\alpha }{2}} |x|^ \delta \Vert \mathcal {P}_\varepsilon f \Vert _{\mathcal {C}^\delta } \\\leqslant & {} N \sup _{\varepsilon \in (0,1]} \varepsilon ^{\frac{\delta -\alpha }{2} }|x|^\delta \varepsilon ^{\frac{\alpha -\delta }{2}} \Vert f\Vert _{\mathcal {C}^\alpha }= N |x|^\delta \Vert f\Vert _{\mathcal {C}^\alpha }. \end{aligned}$$

We move to the case $\alpha <0$. We have

$$\begin{aligned} \Vert f(x+\cdot )- f(\cdot )\Vert _{\mathcal {C}^{\alpha -\delta }}= & {} \sup _{\varepsilon \in (0,1]} \varepsilon ^{\frac{\delta -\alpha }{2}} \Vert \mathcal {P}_\varepsilon f(x+ \cdot )- \mathcal {P}_\varepsilon f(\cdot )\Vert _{\mathcal {C}^0} \\\leqslant & {} \sup _{\varepsilon \in (0,1]} \varepsilon ^{\frac{\delta -\alpha }{2}} |x|^ \delta \Vert \mathcal {P}_\varepsilon f \Vert _{\mathcal {C}^\delta } \\= & {} \sup _{\varepsilon \in (0,1]} \varepsilon ^{\frac{\delta -\alpha }{2}} |x|^ \delta \Vert \mathcal {P}_{\frac{\varepsilon }{2}+\frac{\varepsilon }{2}} f \Vert _{\mathcal {C}^\delta } \\\leqslant & {} N \sup _{\varepsilon \in (0,1]} \varepsilon ^{\frac{\delta -\alpha }{2} }|x|^\delta \varepsilon ^{\frac{-\delta }{2}} \Vert \mathcal {P}_{\frac{\varepsilon }{2}}f\Vert _{\mathcal {C}^0} \leqslant N |x|^\delta \Vert f\Vert _{\mathcal {C}^\alpha }. \end{aligned}$$

The proposition is proved. $\square$

## B: Proofs of the results from Section 3.4 related to the Girsanov theorem

### Proof of Proposition 3.10

If $H=1/2$, then there is nothing to prove; the statement of the proposition follows from the standrad Girsanov theorem for Brownian motion. Otherwise, if $H\ne 1/2$, let us verify that all the conditions of the Girsanov theorem in the form of [32, Theorem 2] are satisfied. Note that even though this theorem is stated in [32] in the one–dimensional setting, its extension to the multidimensional setup is immediate.

First, let us check condition (i) of [32, Theorem 2]. If $H<1/2$, then $\int _0^1 u_s^2 ds\leqslant M^2<\infty$ and thus this condition is satisfied by the statement given at [32, last paragraph of Section 3.1]. If $H>1/2$, then

$$\begin{aligned} \bigl [D_{0+}^{H-1/2}u\bigr ](t)=Nu_tt^{-H+1/2}+N(H-1/2)\int _0^t\frac{u_t-u_s}{(t-s)^{H+1/2}}\,ds, \end{aligned}$$

where $D_{0+}^{\beta }$ denotes the left-sided Riemann–Liouville derivative of of order $\beta$ at 0, $\beta \in (0,1)$, see [32, formula (4)]. Therefore, taking into account that $H<1$ and assumption 3.27,

$$\begin{aligned} \int _0^1\Bigl |\bigl [D_{0+}^{H-1/2}u\bigr ](t)\Bigr |^2\,dt\leqslant NM^2+ N\int _0^1\Bigl (\int _0^t\frac{|u_t-u_s|}{(t-s)^{H+1/2}}\,ds\Bigr )^2\,dt<\infty \,\,\text {a.s.}. \end{aligned}$$

Thus, $D_{0+}^{H-1/2}u\in L_2([0,1])$ a.s. and hence condition (i) of [32, Theorem 2] is satisfied.

Now let us verify condition (ii) of [32, Theorem 2]. Consider the following kernel:

$$\begin{aligned} K_H(t,s):=(t-s)^{H-1/2}F(H-1/2,1/2-H,H+1/2,1-t/s),\quad 0\leqslant s\leqslant t\leqslant 1, \end{aligned}$$

where *F* is the Gauss hypergeometric function, see [12, equation (2)]. It follows from [12, Corollary 3.1], that there exists a constant $k_H>0$ and *d*–dimensional Brownian motion ${\widetilde{W}}$ such that

$$\begin{aligned} B^H(t)=k_H\int _0^t K_H(t,s)\, d{\widetilde{W}}_s,\quad 0\leqslant t\leqslant 1. \end{aligned}$$

Consider a random variable

$$\begin{aligned} \rho :=\exp \Bigl (-\int _0^1 v_s d{\widetilde{W}}_s-\frac{1}{2}\int _0^1 |v_s|^2 ds\Bigr ), \end{aligned}$$

where the vector *v* is defined in the following way. If $H<1/2$, then

B.1 $$\begin{aligned} v_t:=\frac{\sin (\pi (H+1/2))}{\pi k_H}t^{H-1/2}\int _0^t (t-s)^{-H-1/2}s^{1/2-H}u_s\, ds, \end{aligned}$$

and if $H>1/2$, then

B.2 $$\begin{aligned} v_t:=\frac{\sin (\pi (H-1/2))}{\pi k_H (H-1/2)}\Bigl (t^{1/2-H}u_t+(H-1/2)\int _0^t \frac{u_t-t^{H-1/2}s^{1/2-H}u_s}{{(t-s)}^{H+1/2}}\, ds \Bigr ).\nonumber \\ \end{aligned}$$

Taking into account [32, formulas (11) and (13)], we see that condition (ii) of [32, Theorem 2] is equivalent to the following one: $\;\;{\mathbb {E}}\;\rho =1$. We claim that actually

B.3 $$\begin{aligned} \;\;{\mathbb {E}}\;\exp (\lambda \int _0^1|v_t|^2 dt)\leqslant R(\lambda )<\infty \end{aligned}$$

where

$$\begin{aligned}&R(\lambda ):=\exp (\lambda N(H)M^2)\quad \text {if }H<1/2;\\&R(\lambda ):=\exp (\lambda N(H)M^2)\;\;{\mathbb {E}}\;\exp (\lambda N(H)\xi )\quad \text {if }H\in (1/2,1). \end{aligned}$$

By the Novikov theorem this, of course, implies that $\;\;{\mathbb {E}}\;\rho =1$.

Now let us verify (B.3). If $H<1/2$, then it follows from (B.1) and (3.24) that

$$\begin{aligned} |v_t|\leqslant N(H)Mt^{-H+1/2}, \end{aligned}$$

which immediately yields (B.3).

If $H>1/2$, then we make use of (B.2) and (3.25) to deduce

$$\begin{aligned} |v_t|\leqslant&N(H)Mt^{1/2-H}+N(H)\int _0^t\frac{|u_t| (t^{H-1/2}s^{1/2-H}-1)}{{(t-s)}^{H+1/2}}\, ds\\&+N(H)\int _0^t\frac{|u_t-u_s|t^{H-1/2}s^{1/2-H}}{{(t-s)}^{H+1/2}}\, ds\\ \leqslant&N(H)Mt^{1/2-H}+N(H)\int _0^t\frac{|u_t-u_s|t^{H-1/2}s^{1/2-H}}{{(t-s)}^{H+1/2}}\, ds. \end{aligned}$$

Taking into account assumption (3.27), we obtain (B.3). Thus, by above, condition (ii) of [32, Theorem 2] is satisfied.

Therefore all the conditions of [32, Theorem 2] are satisfied. Hence the process ${\widetilde{B}}^H$ is indeed a fractional Brownian motion with Hurst parameter *H* under ${\widetilde{\mathbb {P}}}$ defined by $d{\widetilde{\mathbb {P}}}/d\mathbb {P}=\rho$.

Finally, to show (3.28), we fix $\lambda >0$. Then, applying the Cauchy–Schwarz inequality, we get

$$\begin{aligned} \;\;{\mathbb {E}}\;\rho ^\lambda =&\;\;{\mathbb {E}}\;\exp \Bigl (-\lambda \int _0^1 v_s d{\widetilde{W}}_s-\frac{\lambda }{2}\int _0^1 |v_s|^2 ds\Bigr )\\ =&\;\;{\mathbb {E}}\;\exp \Bigl (-\lambda \int _0^1 v_s d{\widetilde{W}}_s-\lambda ^2\int _0^1 |v_s|^2 ds+(\lambda ^2-\lambda /2)\int _0^1 |v_s|^2 ds\Bigr )\\ \leqslant&\Bigl [\;\;{\mathbb {E}}\;\exp \Bigl (-2\lambda \int _0^1 v_s d{\widetilde{W}}_s-2\lambda ^2\int _0^1 |v_s|^2 ds\Bigr )\Bigr ]^{1/2} \Bigl [\;\;{\mathbb {E}}\;\exp \Bigl ((2\lambda ^2-\lambda )\int _0^1 |v_s|^2 ds\Bigr )\Bigr ]^{1/2}\\ =&\Bigl [\;\;{\mathbb {E}}\;\exp \Bigl ((2\lambda ^2-\lambda )\int _0^1 |v_s|^2 ds\Bigr )\Bigr ]^{1/2}\\ \leqslant&R(2\lambda ^2)^{1/2} <\infty , \end{aligned}$$

where the last inequality follows from (B.3). This completes the proof of the proposition. $\square$

### Proof of Lemma 3.11

We begin with establishing bound (3.29). Fix $n\in \mathbb {N}$ and let us split the inner integral in (3.29) into two parts: the integral over $[0,\kappa _n(t)-(2n)^{-1}]$ and $[\kappa _n(t)-(2n)^{-1},t]$. For the first part we have

B.4 $$\begin{aligned} I_1(t)&:=\int _0^{\kappa _n(t)-(2n)^{-1}}\frac{(t/s)^{H-1/2} |f_{\kappa _n(t)}-f_{\kappa _n(s)}|}{(t-s)^{H+1/2}}\,ds\nonumber \\&\leqslant [f]_{\mathcal {C}^\rho }t^{H-1/2} \int _0^{\kappa _n(t)-(2n)^{-1}} s^{1/2-H} |\kappa _n(t)-\kappa _n(s)|^\rho (t-s)^{-H-1/2}\,ds\nonumber \\&\leqslant N[f]_{\mathcal {C}^\rho }t^{H-1/2} \int _0^{\kappa _n(t)-(2n)^{-1}} s^{1/2-H} |t-s|^{\rho -H-1/2}\,ds\nonumber \\&\leqslant N[f]_{\mathcal {C}^\rho }t^{H-1/2} \int _0^{t} s^{1/2-H} |t-s|^{\rho -H-1/2}\,ds\nonumber \\&\leqslant N[f]_{\mathcal {C}^\rho }t^{\rho -H+1/2}, \end{aligned}$$

where we used bound (3.24), the assumption $\rho -H-1/2>-1$, and the fact that for $s\in [0,\kappa _n(t)-(2n)^{-1}]$ one has

$$\begin{aligned} \kappa _n(t)-\kappa _n(s)\leqslant t-s+1/n\leqslant 3(t-s). \end{aligned}$$

Now let us move on and estimate the second part of the inner integral in (3.29). If $t\geqslant 1/n$, then we have

B.5 $$\begin{aligned} I_2(t)&:=\int _{\kappa _n(t)-(2n)^{-1}}^t\frac{(t/s)^{H-1/2} |f_{\kappa _n(t)}-f_{\kappa _n(s)}|}{(t-s)^{H+1/2}}\,ds\nonumber \\&= t^{H-1/2}|f_{\kappa _n(t)}-f_{\kappa _n(t)-1/n}| \int _{\kappa _n(t)-(2n)^{-1}}^{\kappa _n(t)} s^{1/2-H} (t-s)^{-H-1/2}\,ds\nonumber \\&\leqslant N [f]_{\mathcal {C}^\rho }n^{-\rho }\frac{t^{H-1/2}}{(\kappa _n(t)-(2n)^{-1})^{H-1/2}} \int _{\kappa _n(t)-(2n)^{-1}}^{\kappa _n(t)} (t-s)^{-H-1/2}\,ds\nonumber \\&\leqslant N [f]_{\mathcal {C}^\rho }n^{-\rho }(t-\kappa _n(t))^{-H+1/2}, \end{aligned}$$

where in the last inequality we used that for $t\geqslant 1/n$ one has

$$\begin{aligned} t\leqslant \kappa _n(t)+\frac{1}{n}\leqslant 4\kappa _n(t)-\frac{2}{n}=4\Bigl (\kappa _n(t)-\frac{1}{2n}\Bigr ). \end{aligned}$$

Now, using (B.5) and (B.5), we can bound the left–hand side of (3.29). We deduce

$$\begin{aligned}&\int _0^1\Bigl (\int _0^t\frac{(t/s)^{H-1/2} |f_{\kappa _n(t)}-f_{\kappa _n(s)}|}{(t-s)^{H+1/2}}\,ds\Bigr )^2\,dt\\&\qquad \leqslant N\int _0^1 I_1(t)^2\,dt+ N\int _0^1 I_2(t)^2\,dt\\&\qquad \leqslant N[f]_{\mathcal {C}^\rho }^2+N[f]_{\mathcal {C}^\rho }^2n^{-2\rho } \sum _{i=1}^{n-1} \int _{\frac{i}{n}}^{\frac{i+1}{n}}|t-\kappa _n(t)|^{1-2H}\,dt\\&\qquad \leqslant N[f]_{\mathcal {C}^\rho }^2+N[f]_{\mathcal {C}^\rho }^2n^{-2\rho } \sum _{i=1}^{n-1} n^{-(2-2H)}\\&\qquad \leqslant N[f]_{\mathcal {C}^\rho }^2+N[f]_{\mathcal {C}^\rho }^2n^{2H-1-2\rho }\leqslant N[f]_{\mathcal {C}^\rho }^2, \end{aligned}$$

where the very last inequality follows from the assumption $\rho >H-1/2$. This establishes (3.29).

Not let us prove (3.30). Using the assumption $\rho >H-1/2$ and identity (3.24), we deduce

$$\begin{aligned} \int _0^1\Bigl (\int _0^t\frac{(t/s)^{H-1/2} |f_{t}-f_{s}|}{(t-s)^{H+1/2}}\,ds\Bigr )^2\,dt&\leqslant [f]_{\mathcal {C}^\rho }^2 \int _0^1\Bigl (\int _0^t s^{-H+1/2} (t-s)^{\rho -H-1/2}\,ds\Bigr )^2\,dt\\&\leqslant N[f]_{\mathcal {C}^\rho }^2. \end{aligned}$$

This proves (3.30). $$\square $$
